# Iterative Usage of Fixed and Random Effect Models for Powerful and Efficient Genome-Wide Association Studies

**DOI:** 10.1371/journal.pgen.1005767

**Published:** 2016-02-01

**Authors:** Xiaolei Liu, Meng Huang, Bin Fan, Edward S. Buckler, Zhiwu Zhang

**Affiliations:** 1 Key Laboratory of Agricultural Animal Genetics, Breeding and Reproduction, Ministry of Education, College of Animal Science and Technology, Huazhong Agricultural University, Wuhan, Hubei, China; 2 Institute for Genomic Diversity, Cornell University, Ithaca, New York, United States of America; 3 Department of Crop and Soil Sciences, Washington State University, Pullman, Washington, United States of America; 4 United States Department of Agriculture (USDA)–Agricultural Research Service (ARS), Ithaca, New York, United States of America; 5 Department of Animal Sciences, Northeast Agricultural University, Harbin, Heilongjiang, China; Microsoft Research, UNITED STATES

## Abstract

False positives in a Genome-Wide Association Study (GWAS) can be effectively controlled by a fixed effect and random effect Mixed Linear Model (MLM) that incorporates population structure and kinship among individuals to adjust association tests on markers; however, the adjustment also compromises true positives. The modified MLM method, Multiple Loci Linear Mixed Model (MLMM), incorporates multiple markers simultaneously as covariates in a stepwise MLM to partially remove the confounding between testing markers and kinship. To completely eliminate the confounding, we divided MLMM into two parts: Fixed Effect Model (FEM) and a Random Effect Model (REM) and use them iteratively. FEM contains testing markers, one at a time, and multiple associated markers as covariates to control false positives. To avoid model over-fitting problem in FEM, the associated markers are estimated in REM by using them to define kinship. The P values of testing markers and the associated markers are unified at each iteration. We named the new method as Fixed and random model Circulating Probability Unification (FarmCPU). Both real and simulated data analyses demonstrated that FarmCPU improves statistical power compared to current methods. Additional benefits include an efficient computing time that is linear to both number of individuals and number of markers. Now, a dataset with half million individuals and half million markers can be analyzed within three days.

## Author Summary

Genome-Wide Association Studies (GWAS) can reveal genetic-phenotypic relationships, but have limitations. To control false positives, population structure and kinship are incorporated in a fixed and random effect Mixed Linear Model (MLM). However, because of the confounding between population structure, kinship, and quantitative trait nucleotides (QTNs), MLM leads to false negatives, missing some potentially important discoveries. Here, we present a new method, Fixed and random model Circulating Probability Unification (FarmCPU). FarmCPU performs marker tests with associated markers as covariates in a fixed effect model and optimization on the associated covariate markers in a random effect model separately. This process enables efficient computation, removes the confounding, prevents model over-fitting, and controls false positives simultaneously. FarmCPU controls false positives as well as MLM with reductions in both false negatives and computing times. Researchers will not only be able to analyze big data, but will also have greater success with fewer mistakes when mapping genes of interest.

## Introduction

Genome-Wide Association Studies (GWAS) use direct statistical tests as opposed to direct genetic inferences carried out in linkage analyses. Associations between a genetic marker and a phenotype happen for many reasons in addition to the genetic linkage between the tested genetic markers and functional causal polymorphisms[1–4]. Population structure and kinship among individuals are two common indirect, non-causal associations that lead to false positives[5–7]. The most effective strategy to eliminate false positives is either 1) fitting population structure as covariates in a General Linear Model (GLM)[8], or 2) fitting both population structure and each individual’s total genetic effect as covariates in a Mixed Linear Model (MLM)[9] to make adjustments for testing markers.

Population structure is normally represented by proportions of individuals belonging to subpopulations, commonly known as the **Q** matrix[10,11], or by principal components (PCs)[8,12,13] derived from genetic markers covering the whole genome. Because subpopulations in the **Q** matrix are fitted as fixed effects, the statistical tests on genetic markers (**S**) can be performed with **GLM**, one marker at a time. The model can be conceptually presented as **y** = **Q**+**S**+**e**, where **y** and **e** are phenotype and residuals, respectively. This model is also known as the **Q** model.

Similarly, the entire set of genetic markers can be used to derive a kinship (**K**) matrix to define the relationship among individuals. Total genetic effects of individuals are fitted as random effects with variance and covariance structure defined by **K**. Conceptually, **MLM** with both **Q** and **K** can be written as **y** = **Q**+**K**+**S**+**e** and is also known as the **Q**+**K** model [9]. Previous studies demonstrated that both the **Q** and **Q**+**K** models control false positives better than naïve models such as the t-test, which only fits the testing markers[8,9]. In general, the **Q**+**K** model performs better than the **Q** model or the **K** model alone when they can not be inclusively represented each other[9,14].

Compared with GLM, MLM is much more computing intensive. Many algorithms have been developed to reduce the computational burden, including EMMA[15] (Efficient Mixed-Model Association), EMMAX[16] (EMMA eXpedited), P3D[17] (Population Parameters Previously Determined), GEMMA[18] (Genome-Wide Efficient Mixed-Model Association), FaST-LMM[19] (Factored Spectrally Transformed Linear Mixed Model), and GRAMMAR-Gamma[20] (fast variance components-based two-step method). However, the statistical power of these algorithms remains the same as the regular MLM.

Another problem with MLM is that its advantage disappears for complex traits when they are associated with population structure. The MLM method was compared with a naïve test (without control over population structure and kinship) in an association study on 107 traits from 199 *Arabidopsis thaliana* individuals genotyped at 250,000 Single Nucleotide Polymorphisms (SNPs)[21]. Both the MLM and naïve methods revealed the known genes without obvious inflation of P values in statistical tests on traits associated with disease resistance, development, and ionomics. However, for traits of flowering time, the naïve method encountered inflated P values; consequently, the signals of known flowering time genes were indistinguishable from the background noise. In contrast, the MLM method controlled inflation well, but the signals of known genes also faded into the background, similar to the naïve method. Thus, for complex traits associated with population structure such as flowering time, incorporating **Q** and **K** in a MLM controls P-value inflation well, but also weakens the real associations.

Two strategies have been developed to solve the confounding problem and improve statistical power for MLM methods. The first strategy, the Compressed MLM (CMLM), clusters individuals into groups and fits genetic values of groups—rather than genetic effects of individuals—as random effects. The CMLM method improves statistical power compared to regular MLM methods[17]. Furthermore, the Enriched CMLM (ECMLM), continually improves statistical power by optimizing the group kinship definition, rather than using the average kinship algorithms constantly[22].

The second strategy changes the definition of kinship among individuals. Only the associated genetic markers are used as pseudo Quantitative Trait Nucleotides (QTNs) to derive kinship instead of all, or a random sample of genetic markers. Pseudo QTNs are expected to closely track some of the causative QTNs, and are selectively used to derive kinship for a specific testing marker. Whenever a pseudo QTN is correlated with the testing marker, it is excluded from those used to derive kinship. In the FaST-LMM-Select method, a pseudo QTN is considered correlated if it is within a 2Mb interval on either side of the testing marker[23]. Instead of using a 2Mb interval, the Settlement of MLM Under Progressively Exclusive Relationship (SUPER) method applies a threshold on Linkage Disequilibrium (LD) between the pseudo QTNs and the testing marker. Selectively including and/or excluding pseudo QTNs to derive kinship for a specific testing marker improves statistical power compared to deriving a overall kinship from all, or a random sample of genetic markers[24].

Both above strategies conduct genetic marker tests one at a time. However, testing multiple markers simultaneously is more advantageous, and can be done by fitting pseudo QTNs in addition to the testing markers in a stepwise MLM, named Multi-Locus Mixed-Model (MLMM)[25]. The overall kinship derived from all available markers is used to define the variance and covariance structure of individuals' genetic effects. After the pseudo QTNs have converged in the final stage of the regression, the P values of pseudo QTN markers are calculated from the MLM with all pseudo QTNs as covariates. Then, genetic markers are tested one at a time with all pseudo QTNs included as covariates in a MLM. The MLMM method outperforms the regular MLM.

Our objective was to develop an improved statistical method that completely eliminates the confounding, and simultaneously improves statistical power and reduces computing time.

### Idea

**Fig 1:**
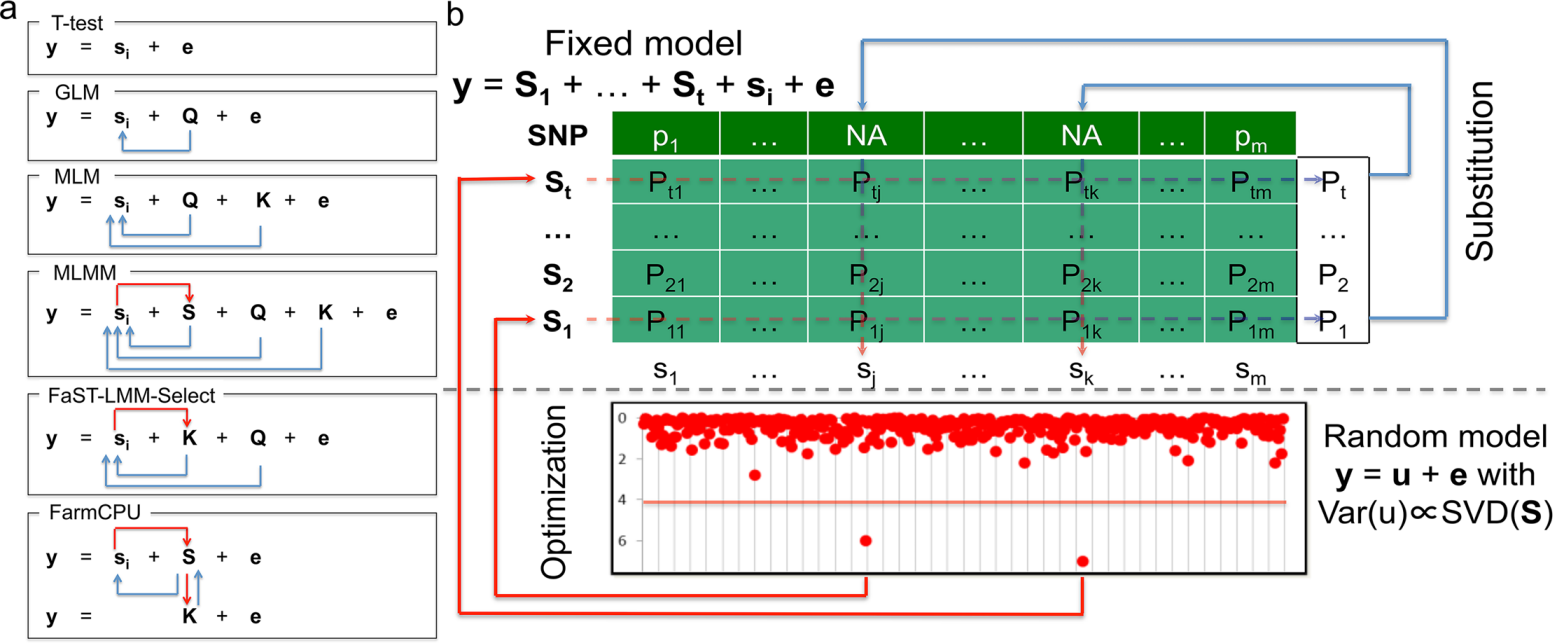
Conceptual development and procedure of FarmCPU. The proposed method, FarmCPU, was inspired by the method development demonstrated on the left panel **(a)**. These methods start with a naïve model (e.g. t-test) that tests marker effect, one at a time, i.e. i^th^ marker (s_i_), on the phenotype (**y**) with a residual effect (**e**). Next, GLM controls false positives by fitting population structure (**Q**) as covariates to adjust the test on genetic markers indicated by the blue arrows. MLM fits both **Q** and kinship (**K**) as covariates. However, both **Q** and **K** remain constant for testing all the markers. Neither **Q** nor **K** receives adjustment from association tests on markers. MLMM add pseudo QTNs as additional covariates (**S**). These pseudo QTNs are estimated through a stepwise regression procedure. Consequently, these pseudo QTNs receive adjustment from association tests on markers as indicated by the red arrow. However, both **Q** and **K** remain constant for testing all the markers. Although similar to MLM, FaST-LMM-Select controls false positives by fitting **Q** and **K** as covariates; the **K** of FaST-LMM-Select is incorporated with association tests on markers as indicated by the red arrow. However, **Q** remains constant. FarmCPU completely removes the confounding between the testing marker and both **K** and **Q** by combining MLMM and FaST-LMM-Select, but allowing a fixed effect model and a random effect model to perform separately. The fixed effect model contains the testing marker and pseudo QTNs to control false positives. The pseudo QTNs are selected from associated markers and evaluated by the random effect model, with **K** defined by the pseudo QTNs. The fixed effect model and random effect model are used iteratively until a stage of convergence is reached, that is, when no new pseudo QTNs are added. The right panel **(b)** displays the fixed effect model above the dashed line and the random effect models below the dashed line. The t pseudo QTNs (**S**_**1**_ to **S**_**t**_) are fitted as covariates to test markers one at a time, e.g., i^th^ marker (**s**_**i**_) in the fixed model. As the pseudo QTNs are fitted as covariates for each marker, Not Available (NA) is assigned as the test statistic for all markers that are also pseudo QTNs—as the genetic marker is completely co-linear to the pseudo QTN marker. However, each pseudo QTN has a test statistic corresponding to every marker, creating a matrix (lightly shaded) with elements of P_ij_, i = 1 to t and j = 1 to m. The most significant P value of each pseudo QTN (the vector on the right of shaded area) is used as the substitution for the NA of the corresponding marker. The pseudo QTNs are optimized by using the SUPER method in the random model to incorporate both test statistics from the fixed effect model and genetic map information in the genotype data. The random effects are the individuals’ genetic effects (**u**) with variance and covariance matrix, Var(**u**), defined by the Singular Value Decomposition (SVD) on the pseudo QTNs by using the FaST-LMM algorithm. The updated set of pseudo QTNs go back into the fixed model. The process continuously repeats until no more pseudo QTNs are added.

Herein, we present a new statistical method that was inspired by the ongoing developments in GWAS analyses, especially the modifications that have improved statistical power. With these developments, statistical methods have been advanced from the naïve method (e.g., t-test) to GLM[8], from MLM[9] to CMLM[17], from FaST-LMM-Select[23] to SUPER[24], and from single marker testing to multiple loci testing (MLMM)[25]. The improvements in statistical power reflect two types of adjustments for testing genetic markers. The first type of adjustment controls false positives and increases power by fitting covariates such as **Q**, **K**, and pseudo QTNs. The second type of adjustment reduces confounding issues by either refining how **K** is derived from all the markers, or selectively including or excluding pseudo QTNs based on their relationship with the testing markers (Fig 1A).

With the only exception on the naïve method, all the above methods incorporate the first type of adjustment. However, only a few methods incorporate the second type of adjustment. For example, CMLM replaces individuals’ genetic effects with groups’ genetic effects. MLMM adds pseudo QTNs as covariates, which are adjusted by using a step-wise regression procedure. The FaST-LMM-Select and SUPER methods selectively include pseudo QTNs to derive kinship for a specific testing marker. However, the confounding between testing markers and covariates still remains a problem. For example, MLMM retains the kinship un-adjusted. FaST-LMM-Select removes markers in kinship that are adjacent (within 2Mb) to testing markers[23]. Yet, a common biological phenomenon is that LD exists at further distances, even across chromosomes. SUPER takes LD into account across the whole genome. However, the exclusion of confounding is limited by the LD threshold[24].

To address the residual confounding problem, our idea was to divided MLMM into two parts: Fixed Effect Model (FEM) and a Random Effect Model (REM), and use them iteratively. FEM contains testing markers, one at a time, and multiple associated markers as covariates to control false positives. For the convenience of illustration, the associated markers were named as pseudo Quantitative Trait Nucleotides (QTNs). To avoid model over-fitting problem in FEM, pseudo QTNs were estimated by REM, where the pseudo QTNs are used to define kinship. FEM and REM are used iteratively until no change on pseudo QTNs. The P values of testing markers and pseudo QTNs are unified at each iteration. Simultaneously, our method completely controls false positives, eliminates confounding, and improves computational efficiency through the following four strategies:

1. Use a single marker test with a FEM to retain efficient computation and to completely remove the confounding between kinship and the testing marker.
2. Include pseudo QTNs as covariates in the FEM to control false positives.
3. Estimate pseudo QTNs by using a maximum likelihood method in a REM to incorporate a map of markers and avoid model over-fitting.
4. Unification of P values of pseudo QTNs in conjunction with tests on the other markers.

The first strategy gives the benefits of efficient computation and the elimination of confounding between kinship and testing markers. The second strategy applies the first type adjustment on the testing markers. The third strategy incorporates a marker map into the estimation of pseudo QTNs by using the SUPER method. The pseudo QTNs are derived through a maximum likelihood method in REM and then used to derive kinship among individuals. Regardless of the number of pseudo QTNs, genetic variance and residual variance are the only unknown parameters. The limited number of parameters avoids the problem of model over-fitting. The fourth strategy enhances the MLMM's algorithm for calculating the P values of pseudo QTNs. Because all pseudo QTNs are examined for each genetic marker tested, we identify and use only the most significant P value among all tests for each pseudo QTN.

Our proposed method requires that the FEM and REM proceed in an iterative fashion. The FEM tests markers, one at a time, and uses a set of pseudo QTNs as covariates. The model can be written as:

$${\text{y}}_{\text{i}}={\text{M}}_{\text{i}1}{\text{b}}_{1}+{\text{M}}_{\text{i}2}{\text{b}}_{2}+\ldots {+\text{M}}_{\text{it}}{\text{b}}_{\text{t}}{+\;\text{S}}_{\text{ij}}{\text{d}}_{\text{j}}{+\text{e}}_{\text{i}}$$ (1)

where y_i_ is the observation of the i^th^ individual; M_i1_, M_i2_,…, M_it_ are the genotypes of t pseudo QTNs, initiated as an empty set; b_1_, b_2_, …, b_j_ are the corresponding effects of the pseudo QTNs; S_ij_ is the genotype of the i^th^ individual and j^th^ genetic marker; d_j_ is the corresponding effect of the j^th^ genetic marker; and e_i_ is the residuals having a distribution with zero mean and variance of $\sigma_{e}^{2}$.

Each of the testing markers receives a P value except those designated as pseudo QTNs and used as covariates. Initially, these pseudo QTN markers are assigned “NA" (Not Available) for their P value. As each pseudo QTN is examined for each testing marker, the NA is replaced with the most significant P value for that pseudo QTN, which becomes the P value of its corresponding marker. We call this process substitution (Fig 1B).

After substitution, every marker has its own P value. The P values and the associated marker map are used to update the selection of pseudo QTNs by using the SUPER algorithm[24] in a REM as follow:

$${\text{y}}_{\text{i}}={\text{u}}_{\text{i}}+{\text{e}}_{\text{i}}$$ (2)

where y_i_ and e_i_ stay the same as in Eq (1) and u_i_ is the total genetic effect of the i^th^ individual. The expectations of the individuals’ total genetic effects are zeros. The variance and covariance matrix of the individuals’ total genetic effects is $G=2K\sigma_{a}^{2}$, where $\sigma_{a}^{2}$ is an unknown genetic variance and *K* is kinship derived from the pseudo QTNs.

The set of pseudo QTNs that maximizes the likelihood of the REM, Eq (2), is used to replace the pseudo QTNs in the FEM, Eq (1). The iteration stops when no change occurs in the estimated set of pseudo QTNs. We named this method Fixed and random model Circulating Probability Unification (FarmCPU). The FarmCPU procedure is further detailed in the online methods section.

In addition to its potential for increasing statistical power, FarmCPU has two other benefits. First, FarmCPU is computationally efficient. Marker testing is conducted by a FEM that has a computing time complexity linear to the number of markers and individuals. Second, P values for non-pseudo QTN markers are not inflated. All markers influential to phenotype are included in the model, either as pseudo QTNs or as markers associated with pseudo QTNs. Because association tests on all markers are performed with pseudo QTNs as covariates, significant P values are not expected for non-pseudo QTN markers.

By performing association tests on real and simulated data and comparing results to current methods, we demonstrated FarmCPU's improved statistical power, increased computational efficiency, and ability to control false positives, i.e. Type I error.

## Results

We analyzed real data to demonstrate new findings and overlaps with known associated loci by using FarmCPU. We simulated data to examine the null distribution and statistical power under different levels of Type I error and False Discovery Rate (FDR). Simulated data were also used to examine FarmCPU's computational efficiency in response to variations in number of markers and sample size.

### Enrichment on candidate genes in *Arabidopsis thaliana*

**Fig 2:**
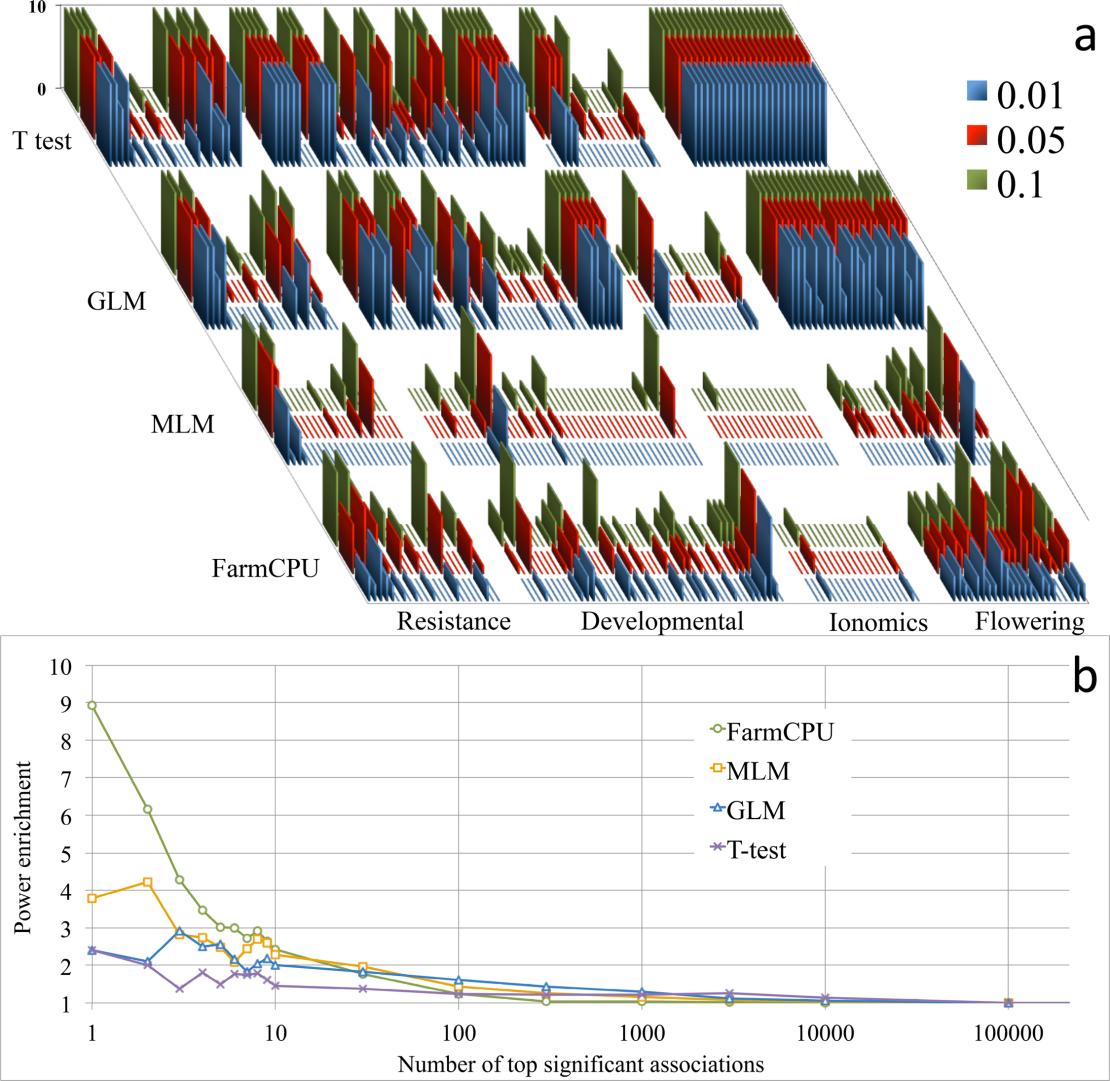
Reanalysis of 107 traits and power enrichment evaluation on 23 flowering time traits in *Arabidopsis thaliana*. Four methods were employed to reanalyze the 107 traits of 199 *Arabidopsis thaliana* samples genotyped at 250,000 SNPs **(a)**, including a naïve method (t-test), GLM, MLM, and FarmCPU. The first three PCs were included in the GLM and MLM to control population structure. FarmCPU did not use any PCs. The horizontal axis indicates the 107 traits grouped into four categories: resistance, developmental, ionomics, and flowering time. The vertical axis indicates the number of associated SNPs at three significance levels (0.01, 0.05 and 0.1) after Bonferroni multiple test corrections. The previous results were replicated by using the naïve and MLM methods. The naïve method, without any control on population structure and kinship, generates many associated SNPs. The associations due to genetic linkage to known genes are indistinguishable from the background noise. In contrast, the MLM method controls the inflation of P values well; however, the associations due to genetic linkage to known genes are also weakened and indistinguishable from the background. The GLM method generates results that are between the naïve method and the MLM method. Interestingly, for each flowering time trait, FarmCPU revealed multiple genetic loci. Enrichment analysis was performed to evaluate the four statistical methods **(b)** on the 23 flowering time traits by using flowering time genes. The random hits are expected to have an enrichment coefficient of 1. For the first hit, the enrichment coefficients are 2.4, 2.4, 3.8, and 8.9 for t-test, GLM, MLM, and FarmCPU, respectively. For the top ten hits, the enrichment coefficients are 1.7, 2.3, 2.8, and 4.0 for t-test, GLM, MLM, and FarmCPU, respectively.

We reanalyzed a published dataset and performed enrichment study on candidate genes to validate the associated loci. When we reanalyzed the 107 traits of 199 *Arabidopsis thaliana* samples genotyped at 250,000 SNPs[21] with FarmCPU and three other methods (naïve, GLM, and MLM), we were able to repeat the previous results by using the naïve and MLM methods (Fig 2A). FarmCPU not only controlled inflation of P values well, but also identified new loci and known associated loci, especially for flowering time (S1 File).

To validate the associated loci on flowering time, we extracted the known candidate genes and conducted an enrichment study. We divided the whole genome into small regions (10,000 base pairs) and categorized each region into either a gene region containing at least one candidate gene or a non-gene region containing no candidate genes. We calculated an enrichment coefficient as the ratio between the numbers of gene regions versus non-gene regions. An enrichment coefficient of 1 is expected for a random association. For the top association, the enrichment coefficient equaled 2.4 for the naïve and GLM methods, 3.8 for the MLM method, and 8.9 for the FarmCPU method (Fig 2B). For the top ten hits, the averaged enrichment coefficients were 1.7, 2.3, 2.8, and 4.0 for naïve, GLM, MLM, and FarmCPU, respectively.

### Overlaps with known loci in multiple species

**Fig 3:**
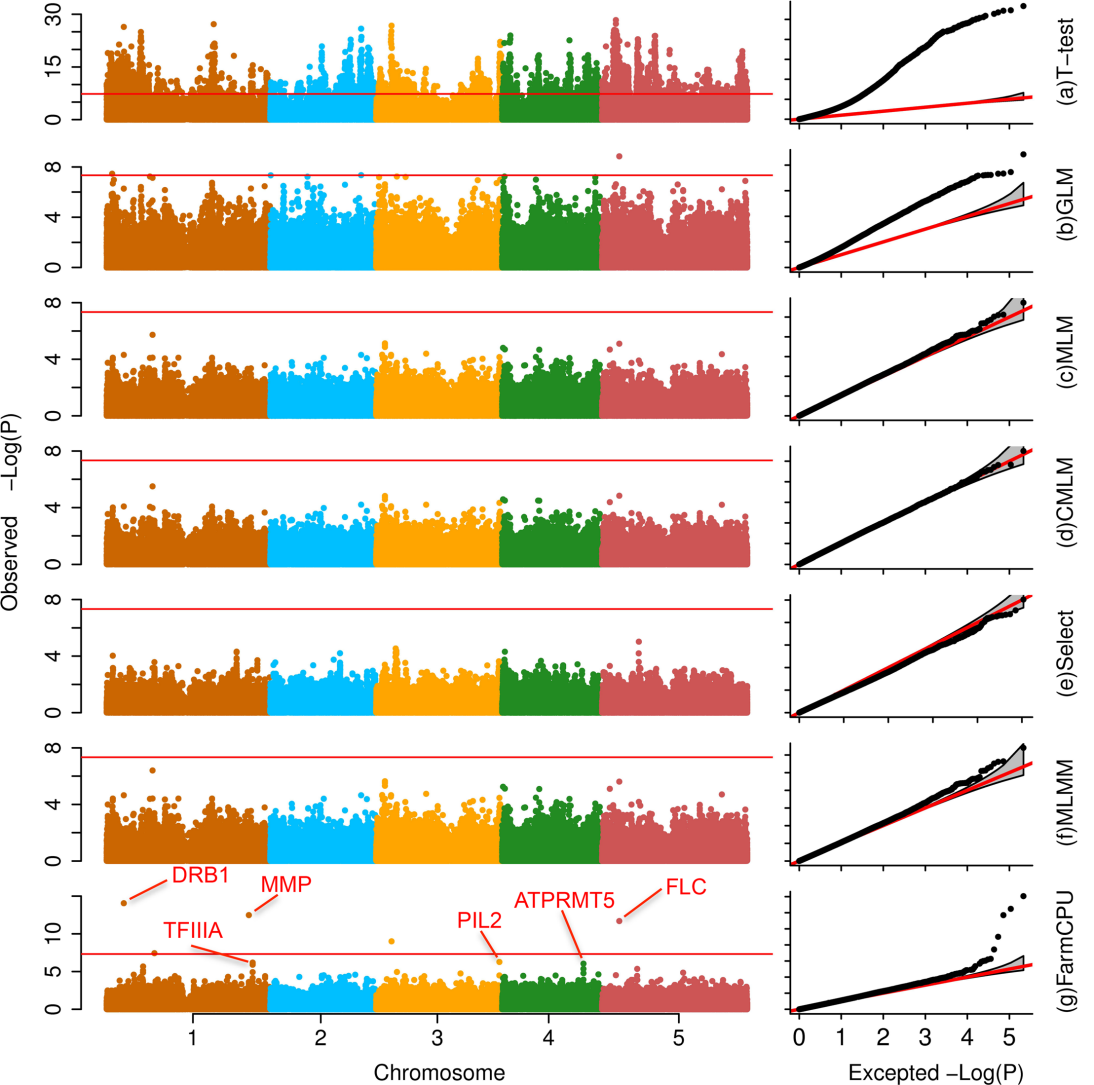
Association studies of flowering time in *Arabidopsis thaliana*. The flowering time at 16°C was measured on 199 *Arabidopsis thaliana* individuals genotyped with 250,000 SNPs. Seven statistical methods were employed to conduct the association studies: **(a)** t-test (naïve method), which tests the additive genetic effect of markers, one marker at a time, with the marker as the only explanatory variable; **(b)** GLM; **(c)** MLM; **(d)** CMLM; **(e)** FaST-LMM-Select; **(f)** MLMM; and **(g)** FarmCPU. All methods, except the t-test, MLMM and FarmCPU, included the first three PCs derived from the genetic markers as covariates. FarmCPU identified five associated SNPs after Bonferroni multiple test correction, including three within a distance of 50,000 base pairs to known genes such as FLC. MLMM identified two associated SNPs after Bonferroni multiple test correction, and overlapped with the five associated SNPs from FarmCPU results. With all other methods, these genes are indistinguishable from the background noise.

We compared FarmCPU with other six methods selected from different categories. These methods are: (1) naïve method (t-test); (2) GLM[8]; (3) MLM[9,26]; (4) CMLM[17]; (5) FaST-LMM-Select[23], and (6) MLMM[25]. Except FarmCPU and t-test, all the other methods included the first three PCs as covariates [14]. We examined datasets from multiple species, including *Arabidopsis thaliana*[21], human[27,28], maize[29], mouse[30], and pig[31]. The results are summarized in Fig 3 and S1–S4 Figs and S1–S6 Tables.

FarmCPU outperformed other methods with respect to controlling inflation of P values, identifying new associated markers, and overlapping with known loci. Taking flowering time at 16°C in *Arabidopsis thaliana* as example, the P values were overwhelmingly inflated under the naïve method (Fig 3). More than 4,000 markers associated with flowering time at a threshold of 1% after Bonferroni multiple test correction. One-half of the markers had P values that deviated from expectation. Thus, the naïve method was unable to distinguish the real signals from the background noise. GLM reduced the inflation, however, 10% of markers still had P values that deviated from expectation. The MLM, CMLM, and Fast-LMM-Select controlled inflation well, but identified no associated markers above the threshold of 1% after Bonferroni multiple test correction. MLMM not only controlled inflation well, but also identified two associated loci above a threshold of 1% after Bonferroni multiple test correction. Besides the two loci identified by MLMM, FarmCPU identified another three associated loci. The new identified loci included the known gene *FLOWERING LOCUS C* (FLC)[32] that controls flowering time in *Arabidopsis thaliana* (S1 Table).

### Null distribution

**Fig 4:**
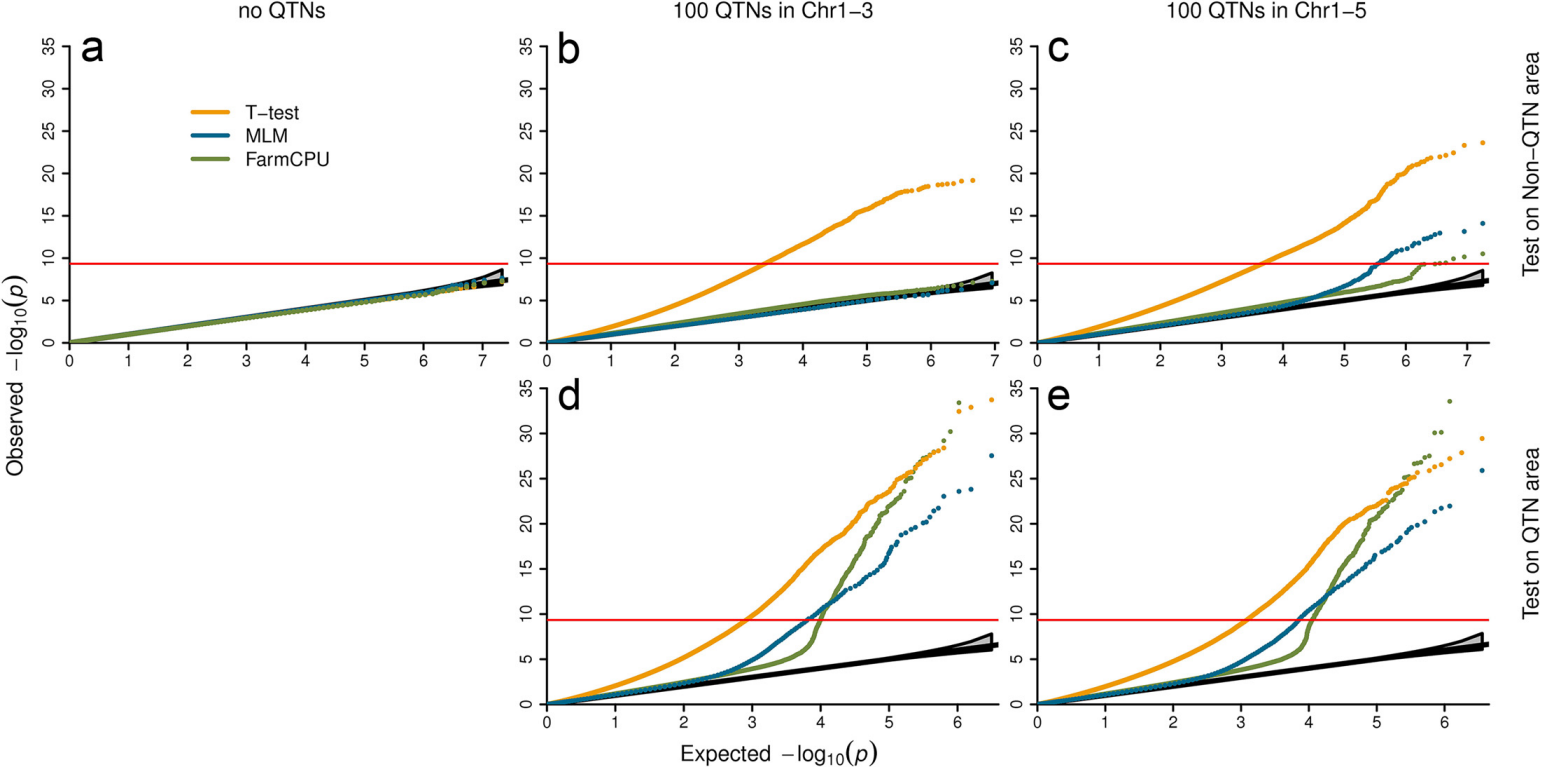
Null distribution of P values under different settings of confounding levels. The first setting contains no QTNs (**left panel**). The second setting restricts QTNs to first three chromosomes and left the rest two chromosomes as control (**middle panel**). The last setting spread QTNs on all five chromosomes (**right panel**). All the SNPs under first setting and all the SNPs on chromosome four and five under second settings were used to derive null distributions (**a** and **b**). The tests of SNPs on chromosomes one to three, including the ones used as QTNs are displayed in the middle panel at bottom (**d**). Under the third setting, SNPs are classified into the QTN areas and the non-QTN areas. A QTN area includes a QTN and its adjacent SNPs within 100,000 base pairs on each side. The rest are the non-QTN areas. The null distribution of non-QTN SNPs is displayed on the top right (**c**) and tests on the SNPs in QTN areas is displayed on the bottom right (**e**). Three statistical methods were examined: FarmCPU, Naïve (t-test) and MLM. The MLM included top six PCs, derived from 10% of SNPs sampled randomly, and used as covariates to control population structure. FarmCPU did not include PCs. The data is a structured *Arabidopsis thaliana* population that includes 1,178 individuals with 214,545 SNP markers. P values were from the association tests on a simulated trait controlled by 100 QTNs with heritability of 50% and QQ plots over 100 replicates are displayed.

We examined null distribution of FarmCPU compared with two other extreme methods. One is the naïve method, t-test, which is expected to exhibit inflation of P values. The other is the MLM method, which controls inflation well. Three datasets with different level of population stratification were used to examine null distribution. The first is *Arabidopsis thaliana* with connected subpopulations. The second is the East Asian lung cancer dataset with mild-isolated subpopulations. The third is the WTCCC1 controls dataset with distinct-isolated subpopulations. The plots of the first three PCs are displayed in S5 Fig. The null distributions are summarized in Fig 4, and S7–S9 Tables. Null distributions were investigated under three confounding level settings:

#### Setting I: Markers associated with no QTNs

We randomly shuffled simulated phenotypes to completely break the association between phenotypes and genotypes. There is no QTN controlling the shuffled phenotypes. No inflation of P values was expected, even with the naïve t-test (Fig 4A). As expected, the three methods behaved the same and have a uniform distribution between 0 and 1.

#### Setting II: Markers associated with QTNs on different chromosomes

We simulated phenotypes with QTNs restricted to partial chromosomes and used the SNPs on other chromosomes to derive the null distribution. For the *Arabidopsis thaliana* data, we put all QTNs on chromosomes 1–3. There were no QTNs on chromosome 4 and 5. The P values of markers on chromosomes 4 and 5 were expected to have uniform distribution. As expected, we observed the P values by using t-test were inflated. The inflation was due to the LD across chromosomes. The t-test was not able to correct the inflation. In contrast, MLM fall into the range of uniform distribution (Fig 4).

Interestingly, we observed that FarmCPU controlled inflation of P values as well as MLM (Fig 4). The pseudo QTNs absorbed the phenotypic variation. To further investigate how FarmCPU controls the inflation on human data, we put QTNs on chromosome 1–10 only. Then we used P values of markers on chromosomes 11–22 and X to examine number of false positives against expected. At different P-value thresholds, the numbers of false positives fall into the 95% confidence interval of expected uniform distribution (S8 and S9 Tables).

#### Setting III: Markers associated with QTNs on same chromosome

As the above two settings are hardly real in practice, we spread QTNs throughout all the chromosomes. For the *Arabidopsis thaliana* data, the simulated QTNs were spread over all the chromosomes (1 to 5). Then, we classified SNPs into QTN areas and non-QTN areas. A QTN area included a QTN and nearby SNPs within 100,000 base pairs on each side. All other SNPs were classified into non-QTN areas. We used SNPs located in non-QTN areas to derive the null distribution. Again, the t-test method exhibited the inflation problem. The MLM showed minor “inflation”, which was possibly due to linkage to the nearby real QTNs. FarmCPU had a lesser inflation than MLM with respect to being closer to the null distribution (Fig 4).

To compare FarmCPU with the other extreme method, t-test, at different resolution of defining false positives, we spread the simulated QTNs across all the chromosomes in human lung cancer data. A marker was considered belonging a non-QTN area is if there is no QTN on either side of the marker within a specific distance (10,000, 50,000, 100,000, 500,000, 1,000,000 base pairs (bps)). Numbers of false positives of FarmCPU and t-test under different P-value thresholds were recorded. The numbers of false positives of FarmCPU were lower in order of magnitude than the ones by using t-test for all combinations between resolutions and levels of P-value thresholds (S7 Table).

As the third setting mixed QTN-areas and non-QTN-areas, the real association signals were also mixed into the null distribution. False positives should be investigated in conjunction of statistical power under same FDR and Type I error. These are summarized in the following section.

### Statistical power under different levels of FDR and Type I error

Genetic markers were classified into the ones on QTN-area and non-QTN area to evaluate statistical power under different levels of FDR and Type I error. The markers on non-QTN areas were used to derive null distribution. For a specific level of Type I error, power was defined as the proportion of QTNs detected. For each level of power, the corresponding FDR was defined as the proportion of false positives (See Materials and Methods section for details). FarmCPU was compared with other common methods under different scenarios, including levels of non-genetic effect, complexity of genetic architecture, and variation of applications such as incorporating PCs.

#### Comparisons with common methods

**Fig 5:**
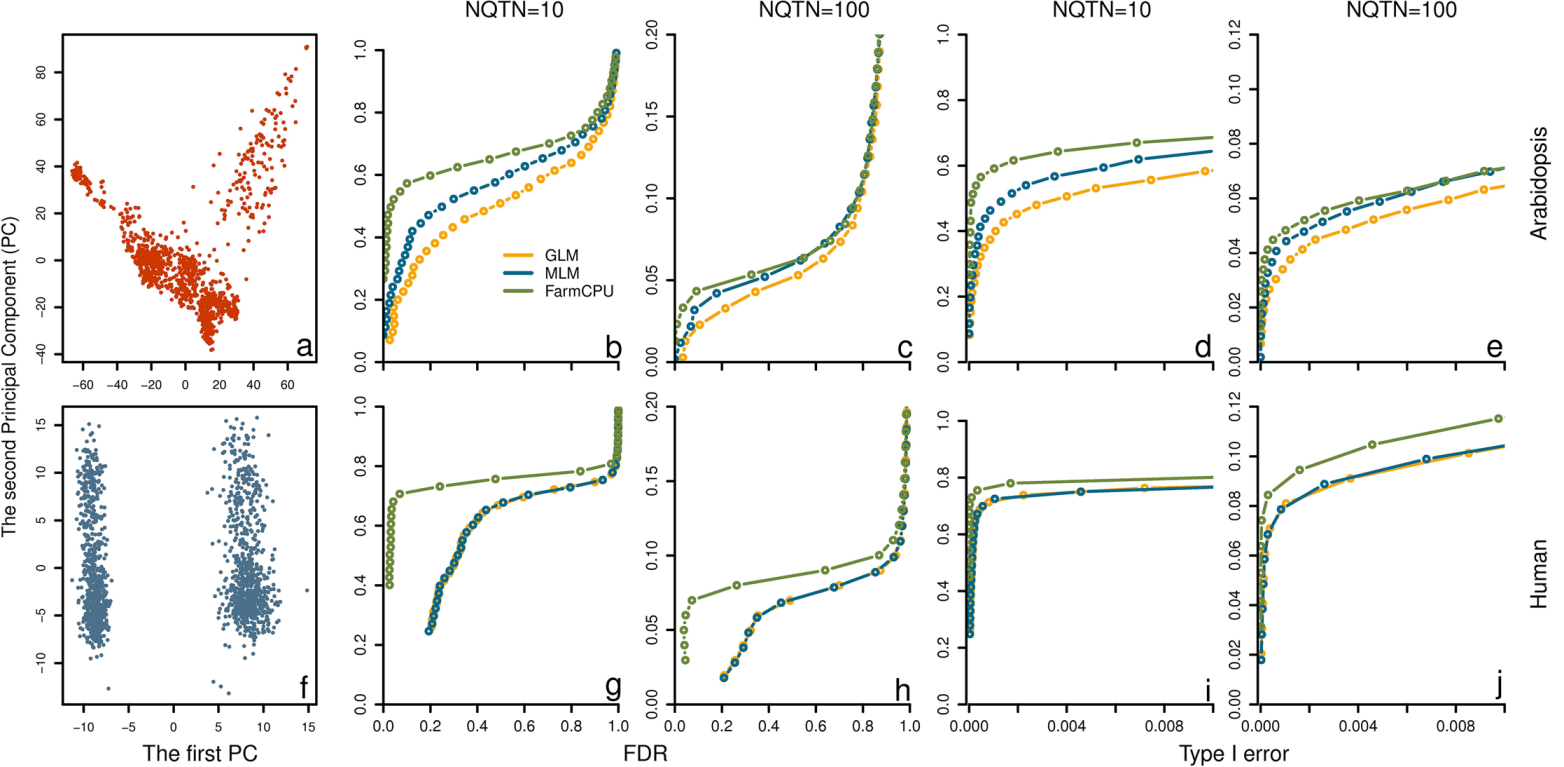
Power in structured populations at different levels. Three methods were employed to examine these populations, including GLM, MLM and FarmCPU. The top panel **(a** to **e)** and bottom panel **(f** to **j)** display the low and high levels of population structure, represented by *Arabidopsis* and human populations, respectively. The dataset from *Arabidopsis* population consists of 1,178 individuals genotyped with 250,000 SNPs. The dataset from human population consists of 1,500 individuals genotyped with 500,000 SNPs. The population structures are displayed by the scatter plot on the first two principal components derived from 10% of SNPs sampled randomly from *Arabidopsis thaliana* **(a)** and human **(f)**, respectively. Additive genetic effects were simulated with 10 and 100 QTNs. The QTNs were randomly sampled from all the SNPs in each dataset. Residuals with normal distribution were added to the genetic effect to form phenotypes with heritability of 0.5. Power was examined under different levels of FDR and Type I error. A positive SNP is considered a true positive if a QTN is within a distance of 50,000 base pairs on either side, otherwise is considered a false positive. Power under different levels of FDR is displayed in subfigures **b, c, g,** and **h**. Power under different levels of Type I error is displayed in subfigures **d, e, i**, and **j**.

We compared FarmCPU with a variety of common methods. First, we compared FarmCPU with three major types of methods: naïve (t-test), GLM and MLM. The comparisons were performed on simulated traits with heritability of 50% controlled by varied number of QTNs (2, 5, 10, and 100). FarmCPU was consistently superior to both GLM and MLM (Fig 5 and S6 and S7 Figs). As expected, we observed a positive correlation between number of true QTNs and number of pseudo QTNs identified in FarmCPU model (S8 Fig). Second, we compared FarmCPU with the three methods that also use information from selected pseudo QTNs: MLMM[25], PC Select[33], and FaST-LMM-Select[23]. The comparisons were performed on a simulated trait with heritability of 50% controlled by 100 QTNs. The FaST-LMM-Select and PC Select methods produced the same results when the same marker-based PCs were used. Therefore, we used FaST-LMM-Select with the first 5 PCs generated from Eigensoft[8,34] (which were the same five suggested by PC Select). The results also demonstrated that FarmCPU outperformed these three methods (S9 Fig). Third, the comparison studies were conducted with several modified MLM methods, such as FaST-LMM, MLMM, and BOLT-LMM[35,36]. Both Power versus FDR and Type I error results showed that FarmCPU outperformed the modified MLM methods (S10 Fig). Statistical power was displayed with different levels of magnification on type I error (S11 Fig). In all comparisons, we found that FarmCPU outperformed the existing methods.

#### Heritability levels

We simulated phenotypes controlled by 100 QTNs with varied levels of heritability, low (30%), moderate (50%), and high (70%). We found that FarmCPU was consistently superior to GLM and MLM. For example, for the trait with 50% heritability, we recorded the number of true and false positives among 100 replicates by using a threshold of 1% after Bonferroni multiple test correction. There were 639, 513, and 520 true QTNs that were detected by FarmCPU, MLM and GLM, respectively, at a cost of 32, 250, and 257 false positives (S12 Fig).

#### Marker density

It is common that QTNs are not part of testing genetic markers unless the markers are dense enough. We performed tests with QTNs included, or excluded, from the genotypic data. The phenotypes were simulated with a heritability of 50% and controlled by 100 QTNs. A QTN was considered detected when a SNP fell into the preset resolution of 10,000, 50,000, or 100,000 base pairs on either side of the QTN. Results indicated that FarmCPU outperforms both GLM and MLM (S13 Fig) in either case, including or excluding QTNs from the testing genetic markers.

#### Incorporation of population structure

Fitting population structure, such as Q matrix or PCs, as covariates is an effective way to capture phenotypic variation due to non-genetic effects, however, the incorporation also causes confounding with causal genes. Therefore, there is side effect for such incorporation. We examined the tolerance of FarmCPU when fitting PCs as covariates in both cases, with and without non-genetic effects. We simulated non-genetic effects by adding and subtracting 50% of phenotypic standard deviation in two ethnic groups (Korean and Japanese) and leaving the other group (Chinese) unchanged. Non-genetic effects cause about 25% of phenotype variation. Even without incorporating PCs, FarmCPU outperformed FaST-LMM that incorporated PCs (S14 and S15 Figs). As PCs can be the factors to capture non-genetic effect as discovered in previous studies[33,37], including PCs as covariates in FarmCPU can also have better power than without the incorporation (S16 Fig).

In situations without non-genetic effects, we also examined the effect of including PCs as covariates using a human population dataset[38]. PCs were modeled in two ways. First, we included PCs in the first iteration only. Second, we retained the PCs through all iterations. The results indicated that FarmCPU was tolerant to fitting PCs in both cases, unless the number of PCs was too big (e.g., 10) (S17 Fig). This result implies that using FarmCPU with PCs fitted as covariates can potentially eliminate false positives due to non-genetic effects that are associated with population structure.

#### Complexity of quantitative traits

First, we examined FarmCPU in cases where a quantitative trait was controlled by major genes and minor genes. We simulated 100 QTNs with effects following a geometric distribution, with the most important gene having an additive effect of parameter *a*. The effect of the i^*th*^ QTN was *a*^*i*^. Two levels of *a* were tested, 0.9 and 0.95. In both cases, FarmCPU outperformed MLM (S18 Fig).

Second, for cases of highly complex traits that are controlled by multi genes and each gene has equal effect. We simulated a trait with 75% heritability controlled by 500 QTNs. All QTNs were located in the first 10 chromosomes, leaving the other chromosomes as the null control. FarmCPU outperformed MLM and GLM (S19 Fig).

#### Effect of iterations

In general, FarmCPU's largest gain in statistical power occurs between the first and second iterations. As the number of iterations increases, the gains in statistical power become smaller (S20 Fig). When the number of iterations is set high enough, a stage is reached after which additional iterations will produce no new pseudo QTNs. We call this the converged stage of iteration. We found that the converged stage always gives the highest statistical power and is ideal when used for a final analysis.

#### Effect of substitution methods

Five substitution methods were examined: onsite, mean, minimum, median, and maximum. Onsite refers to the same method used by the MLMM, which reports P values of pseudo QTNs from the model that includes only the pseudo QTNs, without the testing markers. The other methods use the mean, minimum, median, and maximum P value of each pseudo QTN when examined together with all markers, one at a time. The results showed that the minimum substitution method outperformed other substitution methods (S21 Fig).

#### Effect of stepwise regression

We compared FarmCPU with stepwise GLM that simply removes the kinship from MLMM. The results showed that the simple stepwise regression increases statistical power compared with GLM (S22 Fig). In addition to stepwise regression, FarmCPU gain statistical power from substitution process and optimization of pseudo QTNs using bin method in random effect model.

#### Incorporation of prior knowledge

We compared FarmCPU with GLM in a scenario that included known true QTNs as covariates to provide additional explanation on how does FarmCPU work. When all true QTNs (100%) underlying a trait are known, each genetic marker can be tested by fitting only the true QTNs as covariates in GLM. When the proportion of known QTNs is reduced, the statistical power is reduced. Amazingly, FarmCPU, without any prior knowledge of true QTNs, has higher power than GLM that incorporated 50% known true QTNs as covariates (S23 Fig).

We expected that using prior knowledge in FarmCPU could further improve statistical power. To test this expectation, we examined simulated phenotypes by incorporating true QTNs. The results indicated that the statistical power of FarmCPU improved further with prior knowledge. Additionally, we found that the greater the proportion of true QTNs incorporated, the greater the improvement in statistical power (S24 Fig).

### Computational efficiency

In addition to improved statistical power, FarmCPU is also computationally efficient. We theoretically analyzed the computing time complexity and measured the actual performance for datasets with specific number of markers and sample size. The factors impacting computing time were investigated to further improve computational efficiency.

#### Theoretical complexity

The computing time of the FEM in FarmCPU is linear to number of markers (m) and sample size (n). For a simple trait, because the number of iterations and the number of pseudo QTNs (t) underlying a trait are constant, the computational complexity of the fixed effect model is *O*(nm). For a complex trait, because bigger sample sizes will be able to identify more pseudo QTNs, the computational complexity is linear to m and superlinear to n. When sample size further increases, as the total number of genes is fixed for a trait, the computational complexity is linear to both m and n. The FaST-LMM algorithm is used in REM with a computing time linear to n. Therefor REM in FarmCPU has computational complexity of *O*(n). REM does not involve tests on genetic markers, therefore, takes only a small proportion of computing time—especially when m is relative large. Overall, the computational complexity of FarmCPU is *O*(nm).

We compared the theoretical computational efficiency of FarmCPU with the following common statistical models: GLM, MLM, GRAMMAR-Gamma, FaST-LMM, FaST-LMM-Select, SUPER, and MLMM. Similar to previous comparisons[6,18,20], we decomposed the total computational time into three components: building the **K**, attaining optimization on variance components, and performing association tests. GRAMMAR-Gamma's (implemented in GenABEL) time for association tests is linear with n, but its times for building the **K** matrix and optimization on variance components are the square and the cube of n, respectively. Both FaST-LMM-Select and SUPER use an algorithm from FaST-LMM, which prevents formatting of the **K** matrix. Rather, optimization is directly performed from the singular value decomposition of the genetic markers used to define kinship among individuals. Because the number of such markers (M) is much less than the number individuals, these two models have a computing time complexity that is linear with n. For FarmCPU, times for both optimization on variance components and performing association tests are linear with n. Comparisons of FarmCPU and other common methods are summarized in S10 Table.

The largest memory of the REM in FarmCPU is to store the genotype table with n individuals and t pseudo QTNs. The memory footprint is linear to n and t. FarmCPU's FEM uses only a small proportion of the memory footprint compared with the FarmCPU’s REM. Therefore, FarmCPU's memory complexity is *O*(nt).

#### Actual performances

**Fig 6:**
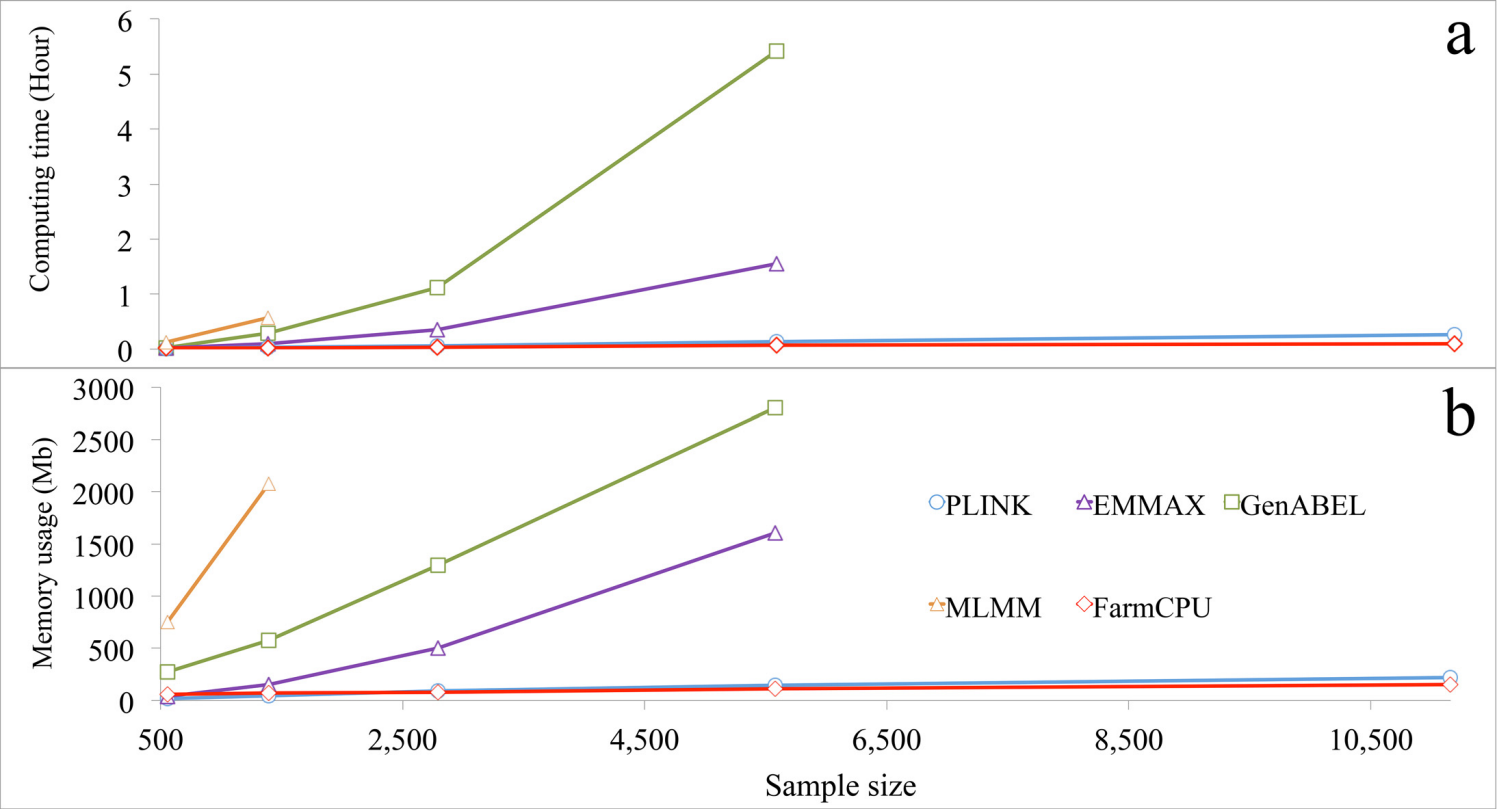
Computing time and memory usage of five software packages. Three statistical models were performed by the five packages: 1) GLM by PLINK; 2) MLMs by EMMAX, GenABEL, and MLMM; and 3) FarmCPU by FarmCPU. Computing time **(a)** and memory usage **(b)** in response to sample size are displayed. The analyses were performed on a laptop (Asus A53S) running a Linux system (Ubuntu 12.10, 64 bit) with a 4.0 Gb of Random-Access Memory (RAM) and an Inter duo Core i3-2310M processor at 2.1 GHz. One core was used for this test. All datasets had 60,000 markers, but response was measured as a function of sample size. The last data point indicates the maximum sample size each software package could process without freezing the computer, except for PLINK and FarmCPU. The limitations for these two software packages were not reached with the maximum sample size examined.

FarmCPU R package was compared with several other packages for actual computing time and memory usage. The other packages were selected to represent different categories. GLM was represented by PLINK[39]. MLMM was represented for MLMM R package. MLM was represented by two packages: EMMAX and GenABEL[40] that uses GRAMMAR-Gamma algorithm. The versions of these packages and parameter settings are summarized in S11 Table. When number of analyzed markers was larger than 30,000, all three MLM-related packages (EMMAX, GenABEL, and MLMM) froze the computer before sample size increased to 6,000. In contrast, both FarmCPU and PLINK completed the analyses even when sample sized was increased to 10,000 (Fig 6 and S25 Fig).

Most statistical methods were developed to solve big data with a focus on either marker size or sample size, but not on both simultaneously. For example, FaST-LMM and GeneABEL could be used for either larger samples, or more markers, but not for both. For a dataset with ten thousands individuals and one million markers, both FaST-LMM and GenABEL failed. FarmCPU completed the analysis on a laptop in less than four hours (S26 Fig). In addition, we tested several methods (BOLT-LMM v2.1, FaST-LMM v2.07, PLINK v1.07 and FarmCPU) on a big dataset including 500,000 individuals with 500,000 SNPs each. A simulated phenotype had a heritability of 30% and was controlled by 3,000 QTNs. All experiments were tested on a RedHat 7.1 operating system running in a server with a 2.2GHz AMD Opteron(tm) Processor 6376, 512GB RAM, 1TB SSD, and 9.4 TB HDD. FarmCPU and PLINK v1.07 completed the data analysis in 3 days, and BOLT-LMM v2.1 complete it in one week, FaST-LMM v2.07 was stopped by the out of memory issue (S27 Fig).

## Discussion

False positives can be reduced by fitting covariates to adjust the association tests on markers. The common covariates are population structure and kinship among individuals. However, the confounding between these covariates and testing markers also produce false negatives. The iterative usage of the fixed effect and random effect models in the FarmCPU method integrates both the markers and the covariates together by optimizing the covariates and using substitution. Testing markers in a fixed effect model makes FarmCPU computationally efficient. The optimization of pseudo QTNs in a random effect model involves only two parameters (genetic and residual variance components) in addition to the number and size of bins of the SUPER GWAS method. Therefore, the problem of model over-fitting is much less compared to including pseudo QTNs and testing markers in the same model. SUPER's bin method takes the map information into account, which effectively reduces redundancy among pseudo QTNs.

### Detection of non-heritable traits

The REM part of FarmCPU has converging problem for the optimization of genetic and residual variance components when one of the components is near zero. This issue is common for a trait with extremely low heritability or a permuted phenotype that has zero heritability. In this case, no pseudo QTNs are associated with the trait. Fortunately, this situation can be detected statistically under a threshold (e.g. 1%) after multiple test correction through the fourth step of the FarmCPU procedure (see online methods). The multiple test correction can be performed with Bonferroni method at lowest computing time. Permutation test costs more time with benefit of improved power (see details in S28 Fig).

### Non-genetic effects

Non-genetic effects cause false positives, especially when they are not correlated with population structure. In this case, it is hard to capture them unless indicators can be identified to capture the non-genetic effects. When the non-genetic effects are correlated with population structure, fitting population structure as covariates reduce false positives. We tested the performance of FarmCPU and MLM when environmental effects were added on phenotypes (using the Chinese, Japanese, and Korean datasets) to contribute an additional 25% of the phenotype variance. These added levels of environmental effects meant that the non-genetic effect was about 170 times greater than the QTN effect. Even in this situation, FarmCPU without fitting PCs as covariates still outperformed MLM that incorporated PCs as covariates (S14 and S15 Figs). Fitting PCs as covariates in FarmCPU further improved statistical power (S16 Fig).

### Unification of testing markers and covariates

Compared with MLMM, FarmCPU uses an improved version for calculating the P values of the pseudo QTNs. MLMM calculates P values using all pseudo QTNs as covariates in the model and excludes testing markers, an approach we named “onsite”. Instead, FarmCPU uses the most significant P value out of each pseudo QTN in conjunction with the tests on all markers. We named this process "substitution". As demonstrated, substitution dramatically improves statistical power (S21 Fig).

### Fresh look of Manhattan plots

FarmCPU's improved statistical power and control on false positives generate a fresh look to the Manhattan and QQ (“Q” stands for Quantile) plots (Fig 3). The skyscrapers over the Manhattan, New York skyline become helicopters above the Manhattan, Kansas skyline. The new appearance of the Manhattan plot better illustrates the trend of increasing marker density. When marker density is high enough, spikes are no longer surprising. Most interesting, and most relevant for data interpretation, is the strongest association in each region of the plot. The QQ plot from FarmCPU is shaped like a hockey stick with a long shaft that joins the observed and expected P values together for the majority of markers. The blade of the hockey stick indicates the associations for the markers with observed P values that deviated from the expectation.

### Data with both large amount of individuals and markers

High marker densities and increased sample sizes, driven by the reduction of genotyping cost, are producing big datasets for analysis[41]. Most statistical methods were developed to solve big data with a focus on either marker size or sample size, but not both. FarmCPU is computationally efficient for both sample size and marker density. Among MLM methods, the CMLM and FaST-LMM methods work best with large sample sizes; the P3D/EMMAX and GRAMMAR-Gamma (Software: GenABEL R package, v 1.8–0) methods work best with high marker densities. For example, a dataset with 10,000 individuals and 10,000 markers freezes a computer running GRAMMAR-Gamma, but only takes 3 minutes with FaST-LMM (Software: FaST-LMM v 2.07). In contrast, a dataset with 1,000 individuals and 1,000,000 markers freezes a computer running FaST-LMM, but only takes 10 minutes for GRAMMAR-Gamma. For a dataset with 10,000 individuals and 1,000,000 markers, computers freeze with both FaST-LMM and GRAMMAR-Gamma. However, FarmCPU solves this dataset in less than four hours (S26 Fig).

URL: The FarmCPU software package (source code, user manual, demo data, and tutorials) is available at http://www.ZZLab.net/FarmCPU.

## Materials and Methods

### FarmCPU method

Our proposed method uses the Fixed Effect Model (FEM) and the Random Effect Model (REM) iteratively. The FEM is employed to test *m* genetic markers, one at a time. Pseudo QTNs are included as covariates to control false positives. Specifically, the FEM can be written as follows:

$${\text{y}}_{\text{i}}={\text{M}}_{\text{i}1}{\text{b}}_{1}+{\text{M}}_{\text{i}2}{\text{b}}_{2}+\ldots {+\text{M}}_{\text{it}}{\text{b}}_{\text{t}}{+\;\text{S}}_{\text{ij}}{\text{d}}_{\text{j}}{+\text{e}}_{\text{i}}$$ (3)

where y_i_ is the observation on the i^th^ individual; M_i1_, M_i2_,…, M_it_ are the genotypes of t pseudo QTNs, initiated as an empty set; b_1_, b_2_, …, b_j_ are the corresponding effects of the pseudo QTNs; S_ij_ is the genotype of the i^th^ individual and j^th^ genetic marker; d_j_ is the corresponding effect of the j^th^ genetic marker; e_i_ is the residual having a distribution with zero mean and variance of $\sigma_{e}^{2}$.

The REM is employed to optimize the selection of pseudo QTNs from markers based on their testing statistics (i.e., P values) and positions by using the SUPER algorithm[24]. Mathematically, the REM can be written as follow:

$${\text{y}}_{\text{i}}={\text{u}}_{\text{i}}+{\text{e}}_{\text{i}}$$ (4)

where y_i_ and e_i_ stay the same as in Eq (3) and u_i_ is total genetic effect of the i^th^ individual. The expectations of the individuals’ total genetic effects are zeros. The variance and covariance matrix of the individuals’ total genetic effects is $G=2K\sigma_{a}^{2}$, where $\sigma_{a}^{2}$ is an unknown genetic variance and *K* is the kinship matrix defined by pseudo QTNs.

The iterative usage of the FEM (1) and the REM (2) is specifically described by the following steps:

Step 1: Set known candidate QTNs as pseudo QTNs, otherwise leave pseudo QTN set empty.
Step 2: Perform association tests by using the FEM with pseudo QTNs as covariates. Genetic markers are fitted as fixed effects, one at a time.
Step 3: Substitution: When the testing marker is one of the pseudo QTNs, the testing marker is not solvable. As each pseudo QTN is examined for every marker, we use the most significant P value for the corresponding marker. We call this process substitution.
Step 4: If no marker passes a threshold (e.g., 1%) after multiple test corrections, take the result from Step 3 as final; otherwise, go to Step 5.
Step 5: Whole genome is divided into bins, and the SNP with the most significant P value in each bin represents the bin. Bin size (e.g. 500; 5,000; and 50,000 kilo base pairs) and number of bins (e.g. from 10 to 100 with step of 10) are optimized in the REM. The combination of size and number with the minimum REML value will be used to select pseudo QTNs. In each iteration, the number of pseudo QTNs is bounded by a function of sample size (e.g., $\sqrt{\text{n}/{\text{log}}_{10}(n)}$) to reflect the ability to detect QTNs in the sample.
Step 6: Elimination linear dependence, or near linear dependence among pseudo QTNs. When two pseudo QTNs have Pearson correlation coefficients above a threshold (e.g., 0.7), remove the less significant pseudo QTN.
Step 7: If no new pseudo QTNs are added, or if the specified maximum number of iterations is reached, stop. Otherwise, go back to Step 2.

### Real data

We used previously published datasets from multiple species that included *Arabidopsis thaliana*, human, maize, mouse, and pig.

We used two datasets of *Arabidopsis thaliana*. The first dataset includes 199 samples, with 216,130 SNPs and 107 phenotypes[21]. When phenotypes had less than 100 records, a minor allele frequency (MAF) of 0.05 was set to filter the SNPs. The second dataset includes 1,179 samples with 214,545 SNPs. One sample was removed because one-half of the genotypic data is missing (URL: http://archive.gramene.org/db/diversity/diversity_view). The kinship matrix and principal components were calculated by GAPIT[42] using 10% of SNPs sampled randomly.

One human dataset, “WTCCC1 controls dataset”, ID # EGAD00000000002, was obtained from EMBL-EBI (The European Molecular Biology Laboratory–The European Bioinformatics Institute)[38]. Respecting the privacy of individual level data, the data is only available under the permission of MalariGen Data Access Committee. This dataset contains 1,500 samples. All samples were genotyped by the Affymetrix_500k SNP Chip and 495,473 markers were used in our simulation study (URL: https://www.ebi.ac.uk/ega/datasets/EGAD00000000002). The kinship matrix and principal components were calculated by GAPIT using 10% of SNPs sampled randomly.

The other human dataset, “East Asian lung cancer dataset”, ID # phs000716.v1.p1, was obtained from dbGaP[27]. Respecting the privacy and intentions of research participants, the data is only available under the permission of NIH (National Institute of Health) and Intramural NCI (National Cancer Institute). The authors applied and got the data through dbGaP Authorized Access. A total of 8,807 samples were used that contain 4,962 lung cancer cases and 3,845 controls. All samples were genotyped by the Illumina Human610_Quadv1_B and Human660W-Quad_v1_A platforms and each sample has 629,968 SNPs (URL: http://www.ncbi.nlm.nih.gov/projects/gap/cgi-bin/study.cgi?study_id=phs000716.v1.p1). The kinship matrix and principal components were calculated using 10% randomly sampled or total SNPs by GAPIT and PLINK, respectively.

The maize genotype dataset includes 2,279 inbred lines, each with 681,258 SNPs. The phenotype is flowering time measured as days to silk[29] (URL: http://www.panzea.org/!#genotypes/cctl). The kinship matrix and principal components were calculated by GAPIT using 10% of SNPs sampled randomly.

The mouse genotype dataset has 1,940 samples (1000 males and 940 females from a heterogeneous stock mice population owned by the Welcome Trust Centre for Human Genetics) with 12,226 SNPs. The phenotype is weight growth intercept[30]. The kinship matrix and principal components were calculated by GAPIT using all SNPs.

The pig genotype dataset has 820 samples (412 Large White and 408 crosses from Large White and Landrace) with 64,212 SNPs. The phenotype is last rib back-fat thickness[31]. All SNPs were used to build the kinship matrix and principal components.

### Simulated phenotypes

We used real genotype datasets from human and *Arabidopsis thaliana* to simulate genetic effects and generate phenotypes by adding residual effects. The QTNs underlying these phenotypes were randomly sampled from the real genotypes. The QTN effects followed a geometric distribution with an additive effect of parameter *a*. The effect of the i^th^ QTN was *a*^*i*^. The parameter *a* was set to 0.9, 0.95, and 1 as described in previous studies[9,17]. Phenotype was simulated as: y = additive effect + residual effect. Additive effect was calculated as: additive effect = QTN matrix * QTN effects. The residual effect, following a Gaussian distribution with mean of 0 and variance of $\sigma_{e}^{2}$, was calculated as: $\sigma_{e}^{2}=(1-h^{2})\sigma_{a}^{2}/h^{2}$, where $\sigma_{a}^{2}$ is the variance of additive effect and *h*^2^ is heritability. Simulations were performed using a variety of QTN numbers and heritability values, and with QTNs included and excluded from the genotypic data for association tests. For each combination of factors, simulations were repeated either 100 or 1,000 times, specified for each experiment.

### Power examination under different levels of Type I error and FDR

Statistical power, Type I error, and FDR were examined simultaneously in association tests on simulated phenotypes with known QTNs, using the method described by Segura et.al[25] and two methods from our previous studies—SUPER[24] and Enriched CMLM[22]. A QTN was considered identified if a positive marker was within a prescribed interval distance (e.g. 50 kb). Power was defined as the proportion of QTNs identified at a threshold of Type I error or FDR. Markers were used to derive the null distribution of negative control if no QTN was within the interval. The null distribution of Type I error was derived from the non-QTN markers. FDR was defined as the proportion of the non-QTN markers among the positive markers.

### Enrichment analysis

The flowering time candidate genes from the database reported by Atwell et. al, (2010, Nature) were used to evaluate the associated SNPs on 23 flowering time traits in *Arabidopsis thaliana*. The whole *Arabidopsis thaliana* genome was divided into gene regions and non-gene regions. The genes and their extensions, 10,000 base pairs on either side, were considered gene regions with a total length of 4,552,815 base pairs (3.9% of whole genome). The remaining areas were considered non-gene regions with total length of 114,616,742 base pairs (96.1% of whole genome). The average hit per base pair was defined by number of associated SNPs divided by total length. The ratio of average hit on gene regions to the average hit on non-gene regions was used as the enrichment coefficient. The random hits were expected to have an enrichment coefficient of 1.

## Supporting Information

S1 Fig Association studies of lung cancer in human. (DOCX)

S2 Fig Association studies of flowering time in maize. (DOCX)

S3 Fig Association studies of weight growth intercept in mouse. (DOCX)

S4 Fig Association studies of last rib backfat thickness in pig. (DOCX)

S5 Fig Population structure in the human and *Arabidopsis thaliana* populations. (DOCX)

S6 Fig Performances of FarmCPU and t-test using East Asian lung cancer data set. (DOCX)

S7 Fig Comparison of Power among different statistical methods used to analyze populations with different levels of population structure. (DOCX)

S8 Fig Relationship between number of true QTNs and number of pseudo QTNs selected by FarmCPU. (DOCX)

S9 Fig Comparison of Power among three related statistical methods. (DOCX)

S10 Fig Performances of Power versus FDR and Type I error. (DOCX)

S11 Fig Comparison of Power among different statistical methods with different levels of Type I error. (DOCX)

S12 Fig Performance of FarmCPU under different heritabilities. (DOCX)

S13 Fig Performance of FarmCPU on markers that are inclusive or exclusive of QTNs. (DOCX)

S14 Fig Power and FDR of MLM and FarmCPU in simulated phenotypes with non-genetic effects. (DOCX)

S15 Fig Power and Type I error of MLM and FarmCPU in simulated phenotypes with non-genetic effects. (DOCX)

S16 Fig Effect of PCs in FarmCPU using simulated phenotypes that include non-genetic effects. (DOCX)

S17 Fig Effect of including population structure as covariates in FarmCPU. (DOCX)

S18 Fig Performances of Power against False Discovery Rate and Type I error in different QTN effect size. (DOCX)

S19 Fig Performances of Power against False Discovery Rate and Type I error in complex traits. (DOCX)

S20 Fig The impact of iterations on FarmCPU. (DOCX)

S21 Fig Impact of substitution methods on P values of pseudo QTNs. (DOCX)

S22 Fig Power versus FDR and Type I error in three methods. (DOCX)

S23 Fig Comparison of FarmCPU and GLM with varying levels of prior knowledge. (DOCX)

S24 Fig Impact of prior knowledge on the Power of FarmCPU. (DOCX)

S25 Fig Comparison of computing time and memory usage among five software packages. (DOCX)

S26 Fig Computing times for analyzing datasets with large numbers of individuals and large numbers of markers. (DOCX)

S27 Fig Computing time on big data analysis. (DOCX)

S28 Fig Performances of different P-value thresholds for selecting pseudo QTNs in FarmCPU. (DOCX)

S1 Table Top 10 associated SNPs identified by FarmCPU on flowering time in *Arabidopsis thaliana*. (DOCX)

S2 Table Top 10 associated SNPs identified by FarmCPU on lung cancer in human. (DOCX)

S3 Table Top 10 associated SNPs identified by FarmCPU on flowering time in maize. (DOCX)

S4 Table Top 10 associated SNPs identified by FarmCPU on weight growth in mouse. (DOCX)

S5 Table Top 10 associated SNPs identified by FarmCPU on back-fat thickness in pig. (DOCX)

S6 Table Significant SNPs detected by FarmCPU are overlapped with previously published results for lung cancer. (DOCX)

S7 Table Observed number of false positives for FarmCPU (FC) versus t-test (T) at different P-value thresholds in simulation. (DOCX)

S8 Table Observed versus expected number of false positives for FarmCPU at different P-value thresholds in the null distribution. (DOCX)

S9 Table Observed versus expected number of false positives for FarmCPU at different P-value thresholds in a structured population. (DOCX)

S10 Table Computing time complexity among statistical methods for Genome-Wide Association Studies. (DOCX)

S11 Table Software versions and codes for building kinship matrices and association tests. (DOCX)

S1 File Association studies of 106 traits in *Arabidopsis thaliana*. (DOCX)
