# Correction: Iterative Usage of Fixed and Random Effect Models for Powerful and Efficient Genome-Wide Association Studies

**DOI:** 10.1371/journal.pgen.1005957

**Published:** 2016-03-18

**Authors:** The PLOS Genetics Staff

The following information is missing from the Funding section: ZZ was supported, in part, by an Emerging Research Issues Internal Competitive Grant from the Washington State University, College of Agricultural, Human, and Natural Resource Sciences, Agricultural Research Center project, the Endowment of Distinguished Professorship for Statistical Genomics, and a research project (No. 126593) from the Washington Grain Commission. The publisher apologizes for the error.
