# Supplementary material for: Iterative Usage of Fixed and Random Effect Models for Powerful and Efficient Genome-Wide Association Studies
Source: PLoS Genet. 2016 Feb 1;12(2):e1005767. doi: 10.1371/journal.pgen.1005767 (PMC4734661; doi:10.1371/journal.pgen.1005767)
Supplement: S1 Fig — (DOCX) [file pgen.1005767.s001.docx]

**
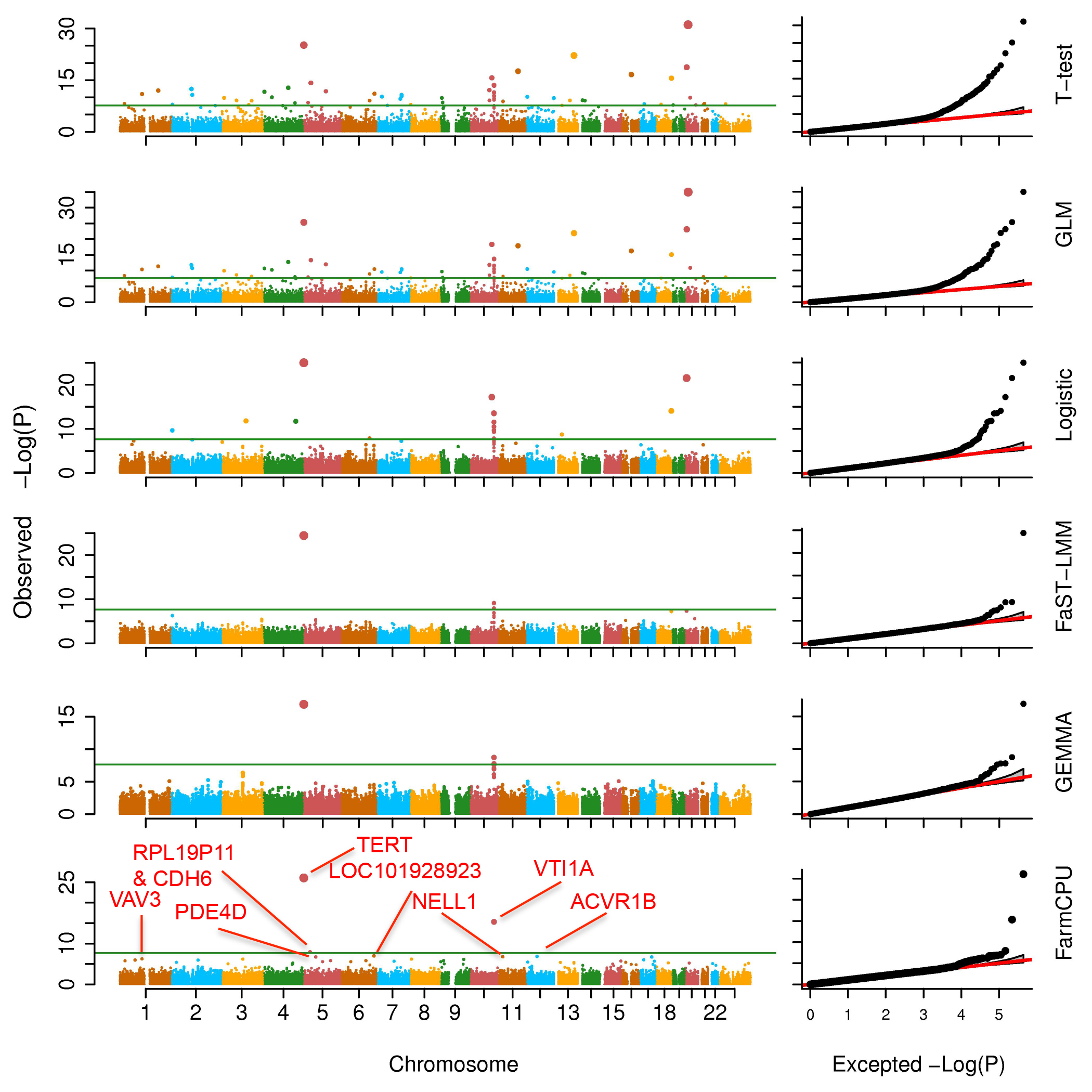
**

**S1 Fig. Association studies of lung cancer in human.** Five methods were used to perform GWAS, t-test, GLM (performed by PLINK), Logistic (Logistic Regression, performed by PLINK), MLM (performed by both FaST-LMM method and GEMMA method) and FarmCPU. The East Asian lung cancer population includes 8,807 samples and each sample was genotyped with 629,968 SNPs (filtered by Minor Allele Frequency > 0.05 and 444,758 SNPs left for association study). GLM and Logistic Regression included the first 5 PCs that derived from all SNPs as covariates to control population structure. GEMMA included the first 4 PCs that derived from 10% of SNPs sampled randomly as covariates. T-test, FaST-LMM, and FarmCPU did not use PCs. As the phenotype is a binary one and fits the assumption of Logistic Regression method, the number of inflation P values in Logistic Regression is ten times less than that in t-test and GLM. MLM controls population structure better than the fixed effects methods and 2 significant SNPs passed a threshold of 1% after a Bonferroni multiple test correction, FarmCPU controls the population structure as well as MLM and one more SNP passed the threshold. From QQ plots, it is hard to say the P values from t-test are inflated, we performed a simulation using the same genotype data to show t-test hits more false positives than FarmCPU in top significant SNPs.
