# Supplementary material for: Iterative Usage of Fixed and Random Effect Models for Powerful and Efficient Genome-Wide Association Studies
Source: PLoS Genet. 2016 Feb 1;12(2):e1005767. doi: 10.1371/journal.pgen.1005767 (PMC4734661; doi:10.1371/journal.pgen.1005767)
Supplement: S2 Fig — (DOCX) [file pgen.1005767.s002.docx]

**
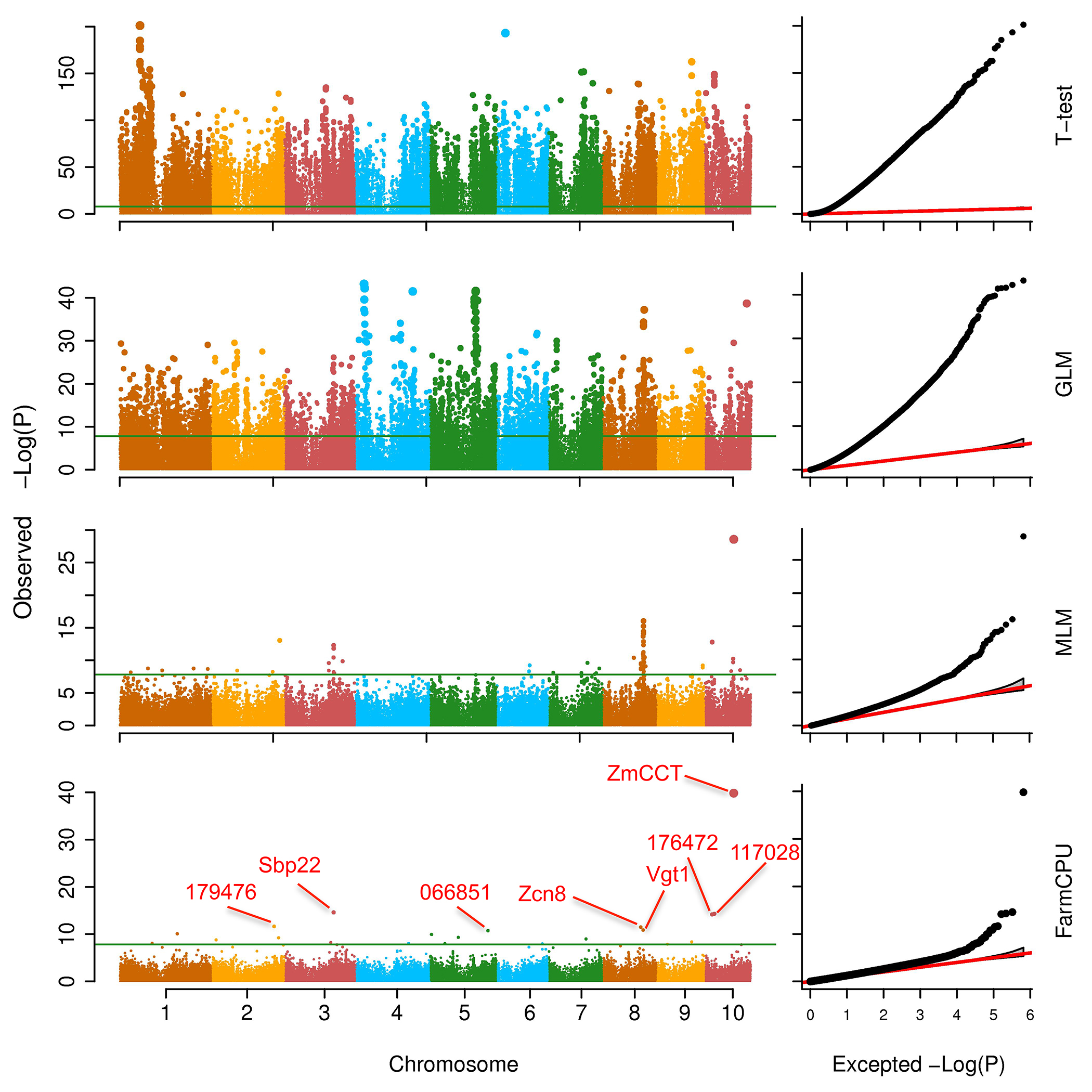
S2 Fig. Association studies of flowering time in maize.** Four methods were used to perform GWAS, t-test, GLM, MLM and FarmCPU. The population includes 2,648 samples and each sample was genotyped with 681,258 SNPs. Both GLM and MLM included the first five PCs, derived from 10% of SNPs sampled randomly, as covariates to control population structure. FarmCPU did not use PCs. From QQ plots on the right panel, results of t-test, GLM and MLM are inflated while FarmCPU not only controls the genetic background well but also hits several candidate genes, such as Zcn8 and Vgt1 that are always hard to distinguish from the other in a single study.
