# Supplementary material for: Iterative Usage of Fixed and Random Effect Models for Powerful and Efficient Genome-Wide Association Studies
Source: PLoS Genet. 2016 Feb 1;12(2):e1005767. doi: 10.1371/journal.pgen.1005767 (PMC4734661; doi:10.1371/journal.pgen.1005767)
Supplement: S3 Fig — (DOCX) [file pgen.1005767.s003.docx]

**
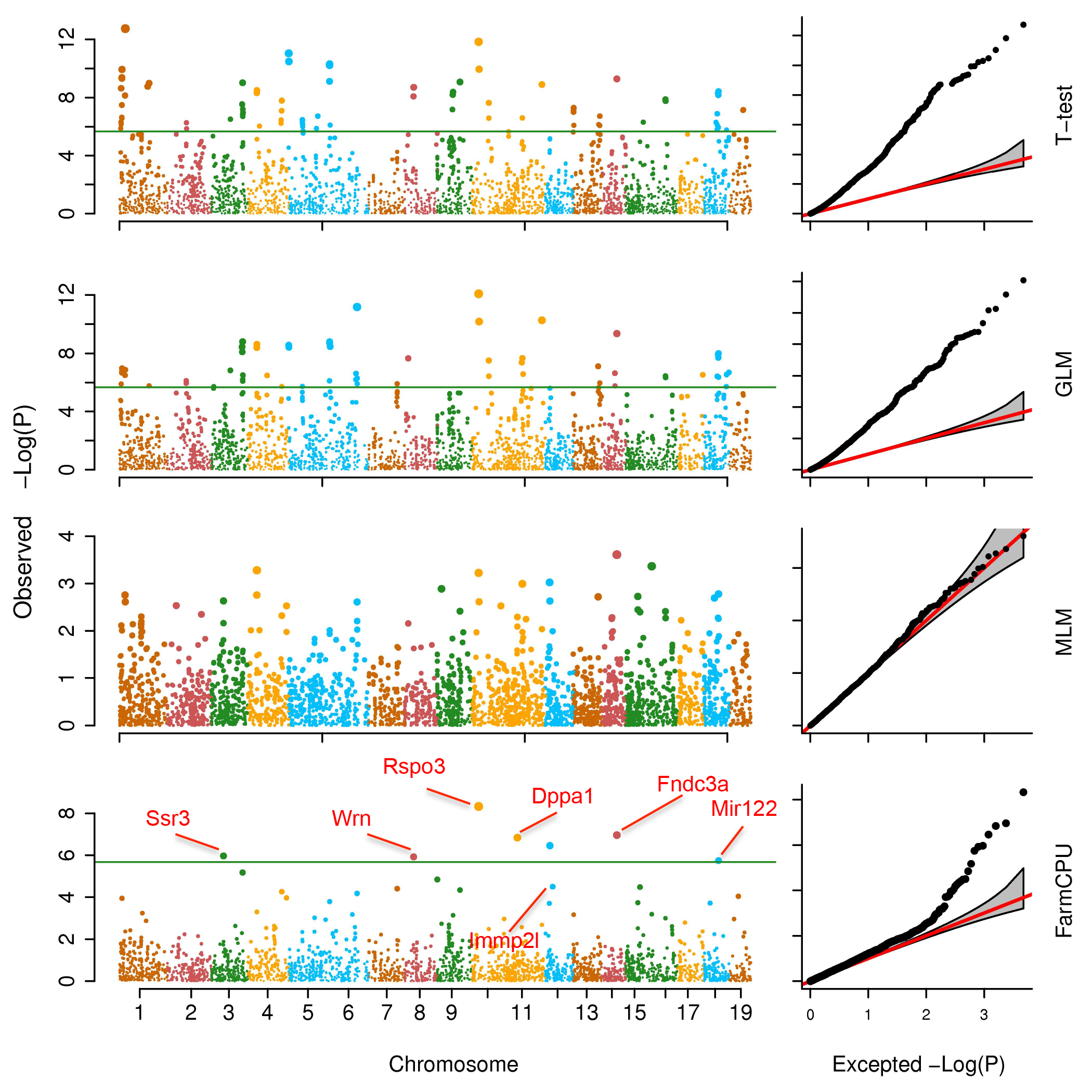
S3 Fig. Association studies of weight growth intercept in mouse.** Four methods were used to perform GWAS, t-test, GLM, MLM and FarmCPU. The population includes 1,940 samples and each sample was genotyped with 12,226 SNPs. Both GLM and MLM included the first five PCs as covariates to control population structure. FarmCPU did not use PCs. From QQ plots on right panel, results of t-test, GLM are inflated and no signals detected in MLM while FarmCPU not only controls the genetic background well but also hits several candidate genes.
