# Supplementary material for: Iterative Usage of Fixed and Random Effect Models for Powerful and Efficient Genome-Wide Association Studies
Source: PLoS Genet. 2016 Feb 1;12(2):e1005767. doi: 10.1371/journal.pgen.1005767 (PMC4734661; doi:10.1371/journal.pgen.1005767)
Supplement: S5 Fig — (DOCX) [file pgen.1005767.s005.docx]

**
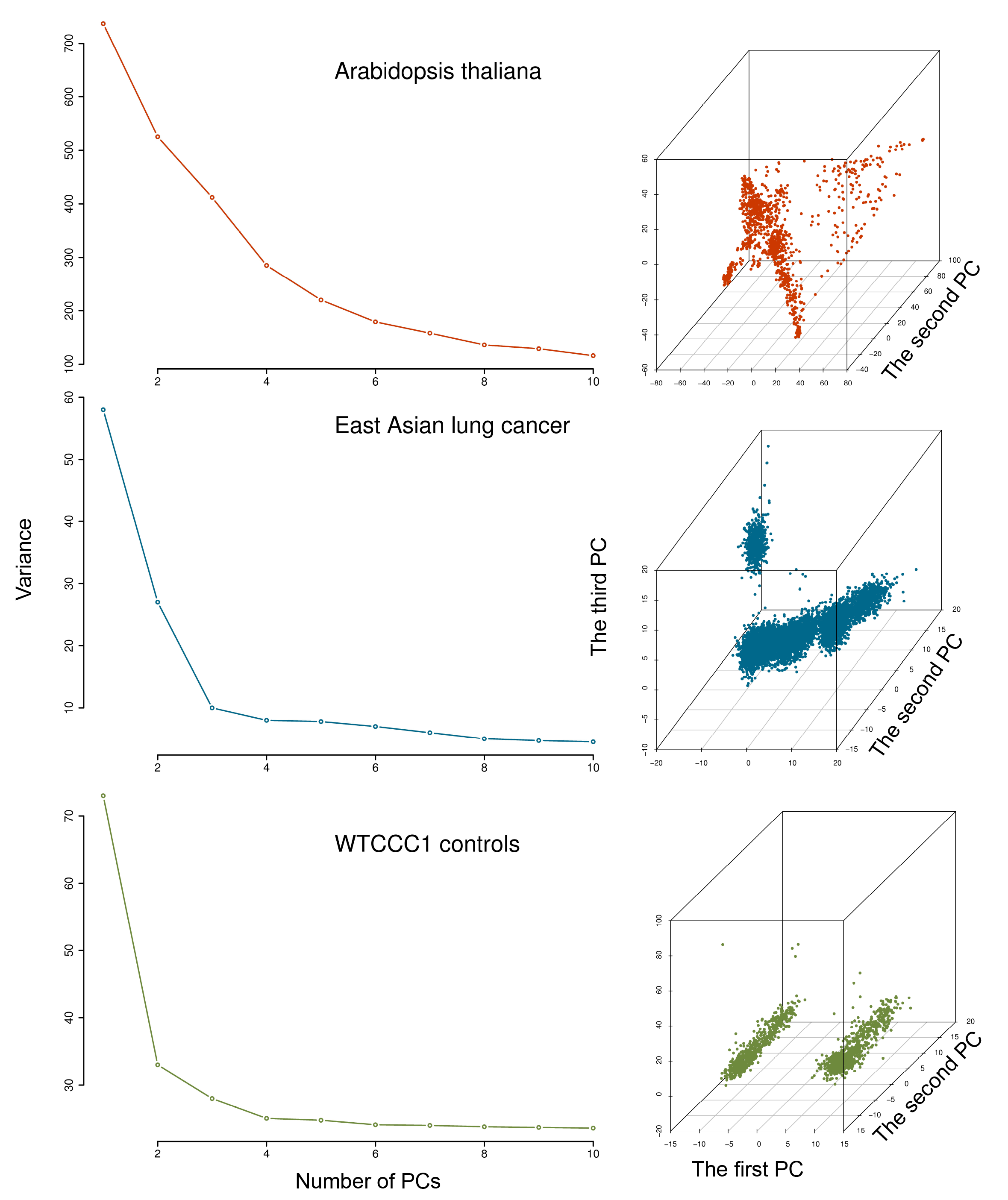
**

**S5 Fig. Population structure in the human and *Arabidopsis thaliana* populations.** The *Arabidopsis* *thaliana* population contains 1,178 *Arabidopsis* *thaliana* lines genotyped with 214,545 SNPs. The East Asian lung cancer dataset contains 8,807 individuals and each individual has 629,968 SNPs. The WTCCC1 controls human population consists of 1,500 individuals genotyped with 495,473 SNPs. The eigenvalues of the first ten PCs are displayed on the left panel; the eigenvalues of the first three PCs are illustrated on the right panel.
