# Supplementary material for: Iterative Usage of Fixed and Random Effect Models for Powerful and Efficient Genome-Wide Association Studies
Source: PLoS Genet. 2016 Feb 1;12(2):e1005767. doi: 10.1371/journal.pgen.1005767 (PMC4734661; doi:10.1371/journal.pgen.1005767)
Supplement: S6 Fig — (DOCX) [file pgen.1005767.s006.docx]

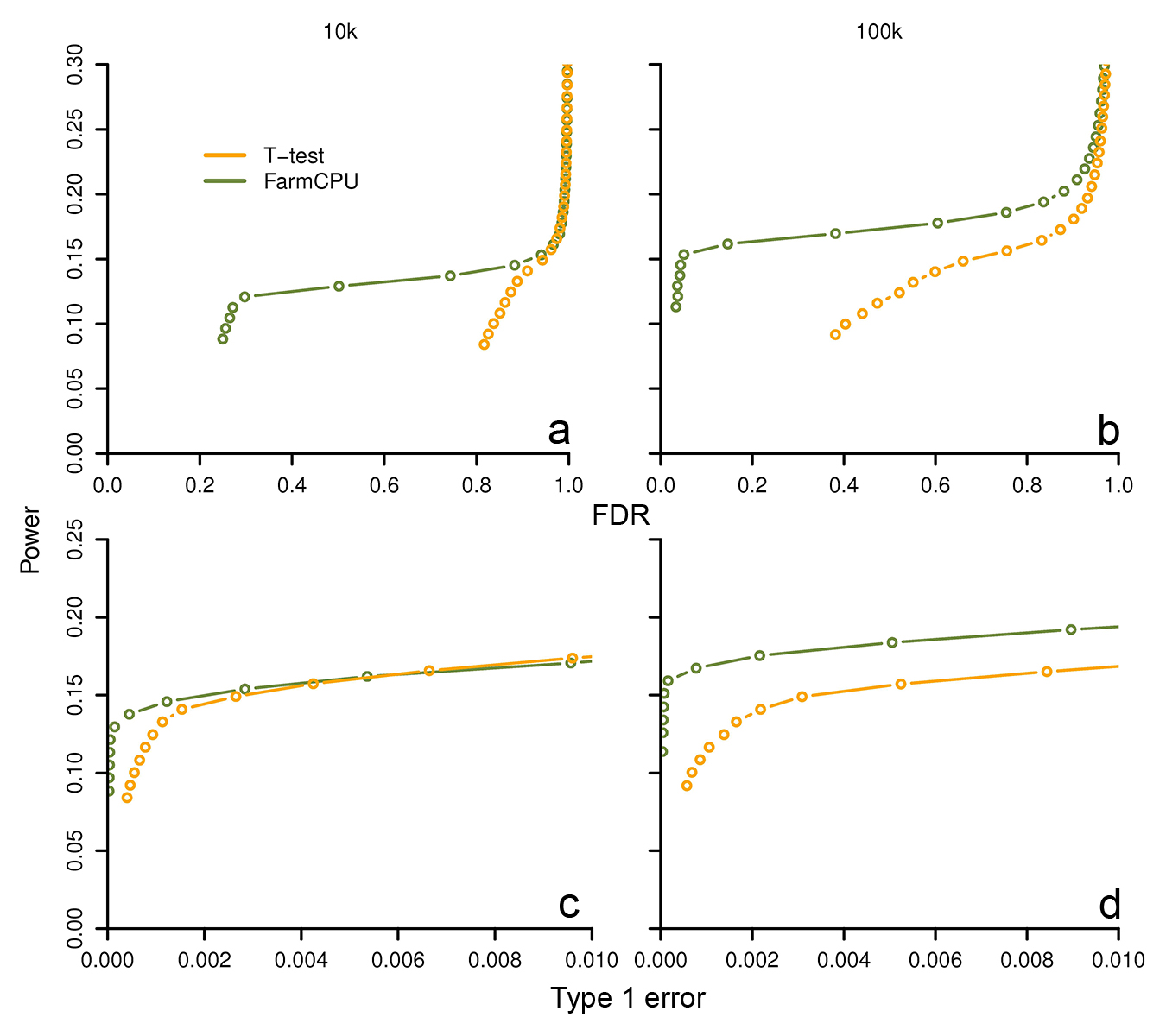


**S6 Fig. Performances of FarmCPU and t-test using East Asian lung cancer dataset.** FarmCPU and t-test were used to test power versus false discovery rate and Type I error using East Asian lung cancer dataset. For each simulated phenotype, additive genetic effects were simulated with 100 QTNs. The QTNs were randomly sampled from all the SNPs. Residuals with normal distribution were added to the genetic effect to form phenotypes with heritability of 0.5. Statistical power was examined under different levels of FDR (**a** and **b**) and Type I error (**c** and **d**). A positive SNP is considered a true positive if a QTN is within a distance of 10,000 and 100,000 base pairs on either side, otherwise is considered a false positive. Power versus FDR and Type I error indicate that compared with t-test, FarmCPU has more chance to hit QTNs in top significant SNPs.
