# Supplementary material for: Iterative Usage of Fixed and Random Effect Models for Powerful and Efficient Genome-Wide Association Studies
Source: PLoS Genet. 2016 Feb 1;12(2):e1005767. doi: 10.1371/journal.pgen.1005767 (PMC4734661; doi:10.1371/journal.pgen.1005767)
Supplement: S7 Fig — (DOCX) [file pgen.1005767.s007.docx]

**
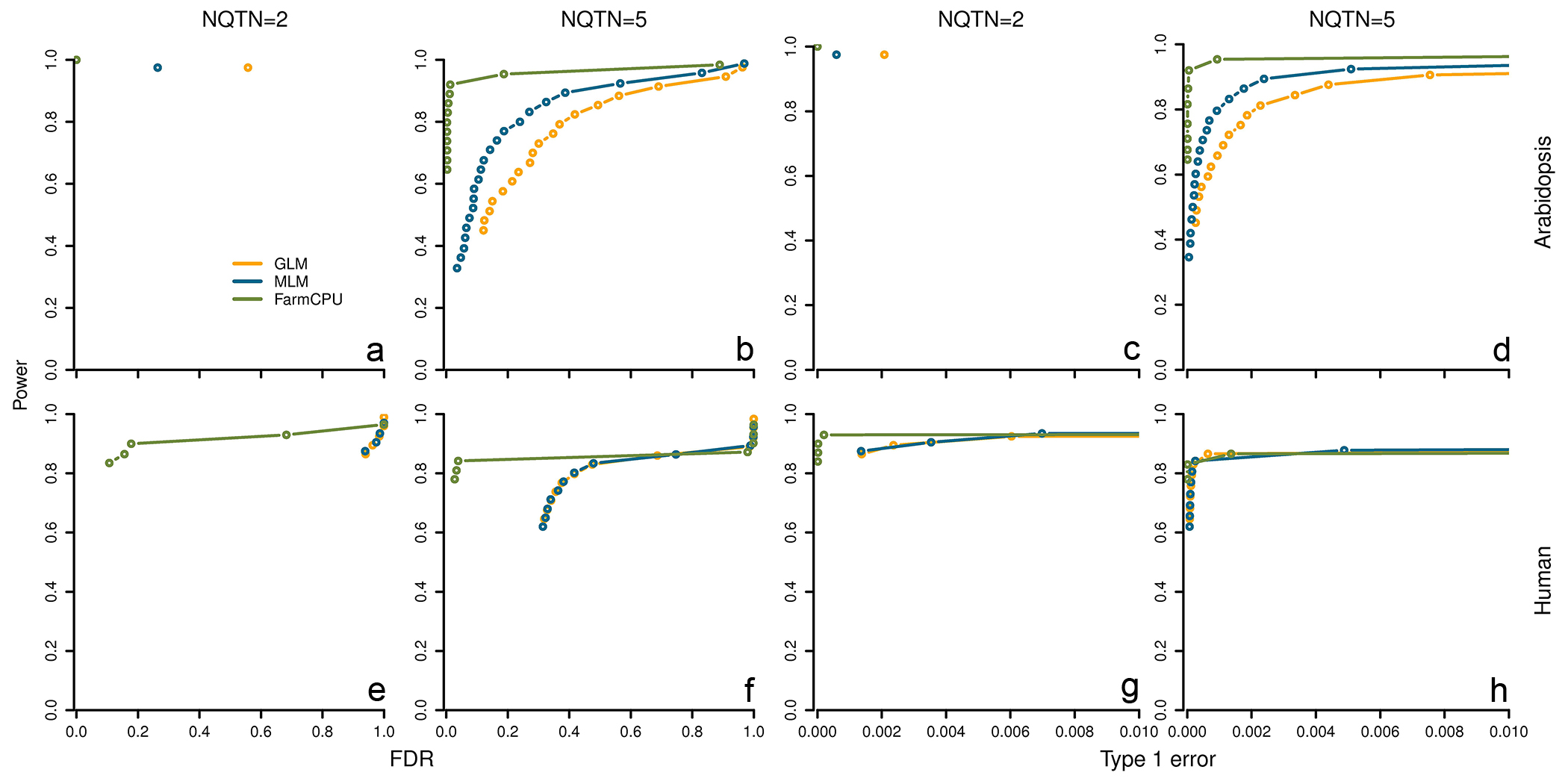
 S7 Fig. Comparison of Power among different statistical methods used to analyze populations with different levels of population structure.** Three methods were examined, GLM, MLM, and FarmCPU. The low and high levels of population structure are represented by *Arabidopsis thaliana* and WTCCC1 controls human populations, respectively. The dataset from the *Arabidopsis thaliana* population consists of 1,178 individuals genotyped with 214,545 SNPs. The dataset from the WTCCC1 controls population consists of 1,500 individuals genotyped with 495,473 SNPs. Additive genetic effects were simulated with 2 and 5 QTNs. The QTNs were randomly sampled from all the SNPs in each dataset. Residuals with normal distribution were added to the genetic effect to form phenotypes with heritability of 0.5. Power was examined under different levels of FDR and Type I error. All markers are sorted with the most significant one on top. A marker is claimed as false positive if no QTN is within a bilateral distance of 50,000 base pairs. For each threshold of FDR, Power is defined as the proportion of QTNs detected **(a, b, e** and **f)**. Similarly, markers without a QTN within a 50,000 base pairs distance are used to derive the empirical null distribution of Type I error. For each threshold of Type I error, Power is defined as the proportion of QTNs detected **(c, d, g** and **h)**.
