# Supplementary material for: Iterative Usage of Fixed and Random Effect Models for Powerful and Efficient Genome-Wide Association Studies
Source: PLoS Genet. 2016 Feb 1;12(2):e1005767. doi: 10.1371/journal.pgen.1005767 (PMC4734661; doi:10.1371/journal.pgen.1005767)
Supplement: S8 Fig — (DOCX) [file pgen.1005767.s008.docx]

**
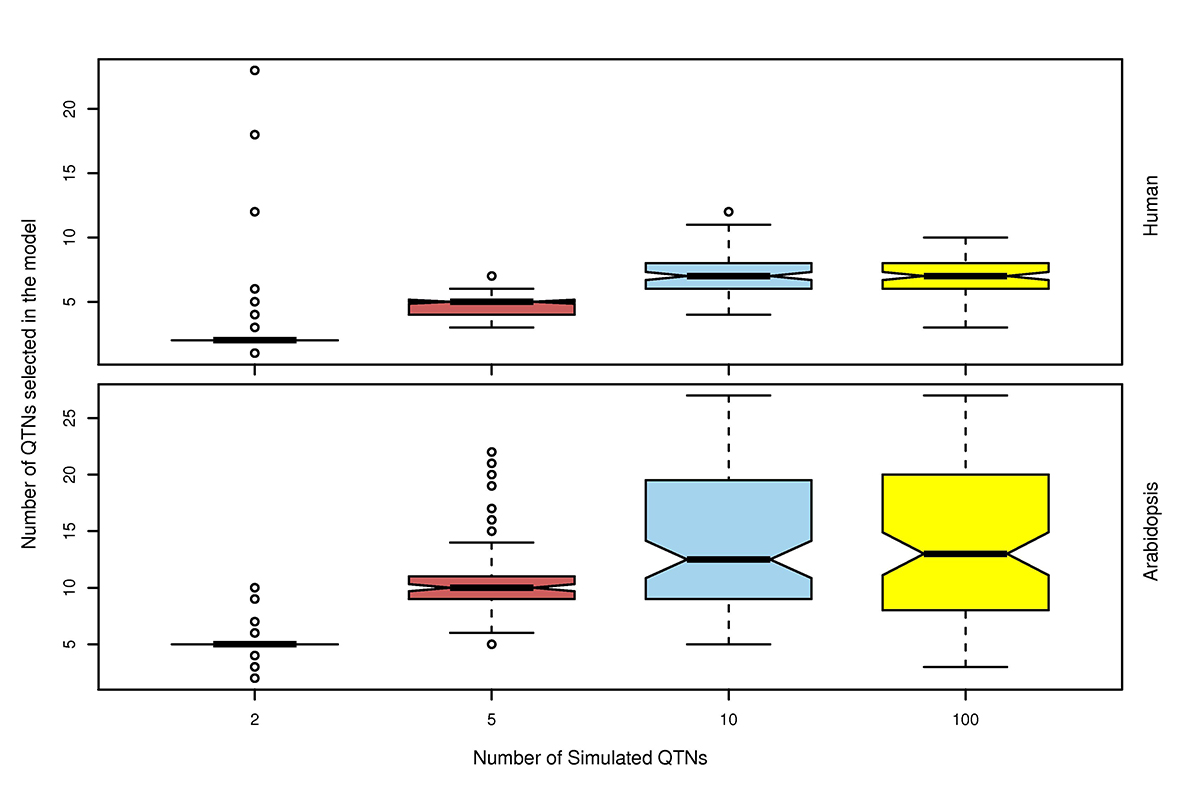
S8 Fig. Relationship between number of true QTNs and number of pseudo QTNs selected by FarmCPU.** The study was conducted on both WTCCC1 controls human population and *Arabidopsis thaliana* population. The WTCCC1 controls population contains 1,500 individuals genotyped with 495,473 SNPs. The *Arabidopsis thaliana* population contains 1,178 individuals genotyped with 214,545 SNP markers. Different numbers of QTNs (2, 5, 10, and 100) were sampled from all the available SNPs in each population and phenotypes were simulated with additive effects of these QTNs at heritability of 0.5. Numbers of pseudo QTNs were recorded to study the correlation between number of true QTNs and number of pseudo QTNs used by FarmCPU. The experiments were replicated 100 times. The numbers of pseudo QTNs are displayed by the boxplots corresponding to different numbers of true QTNs. Results show a strong, positive correlation between number of pseudo QTNs and number of true QTNs.
