# Supplementary material for: Iterative Usage of Fixed and Random Effect Models for Powerful and Efficient Genome-Wide Association Studies
Source: PLoS Genet. 2016 Feb 1;12(2):e1005767. doi: 10.1371/journal.pgen.1005767 (PMC4734661; doi:10.1371/journal.pgen.1005767)
Supplement: S9 Fig — (DOCX) [file pgen.1005767.s009.docx]

**
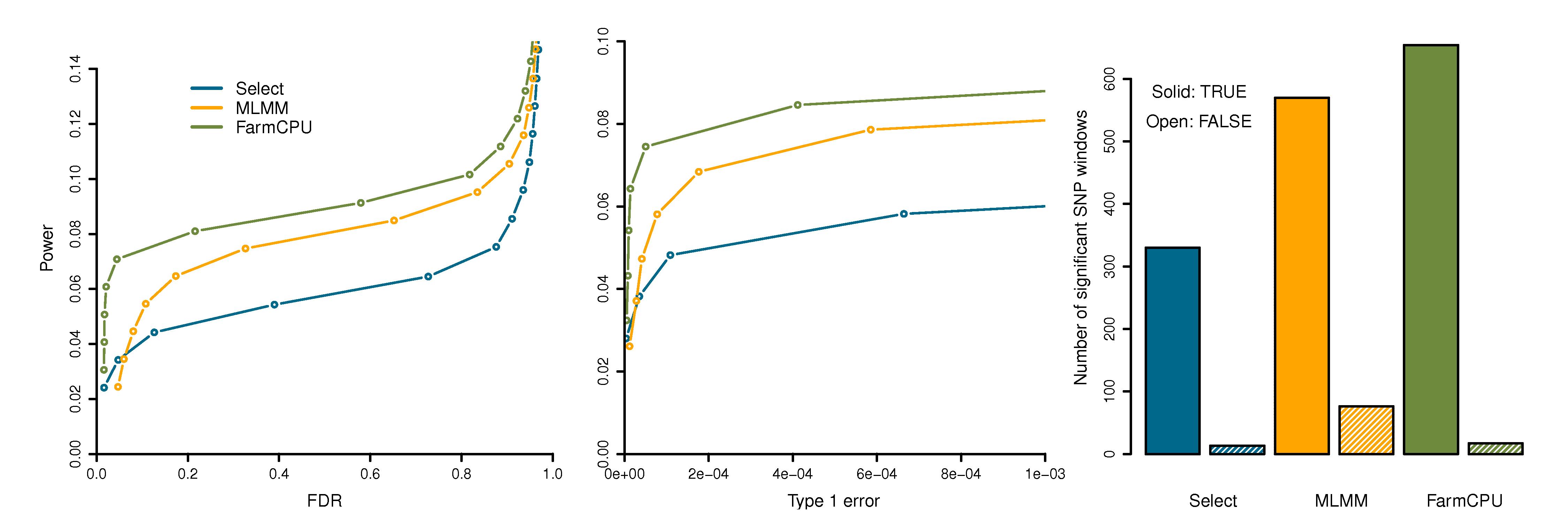
**

**S9 Fig. Comparison of Power among three related statistical methods.** All methods take information from selected pseudo QTNs**.** The three methods are: 1) FarmCPU; 2) MLMM; and 3) Select (PC-Select, Principal Components Select and FaST-LMM-Select, Factored Spectrally Transformed Linear Mixed Model Select). Performance is measured by Power and number of significant SNPs classified into true positives (QTNs) and false positives (non-QTNs). Statistical tests were performed on a trait simulated from WTCCC1 controls population. Additive genetic effects were simulated with 100 QTNs. The QTNs were randomly sampled from all the SNPs. Residuals with normal distribution were added to the genetic effect to form phenotypes with heritability of 0.5. FaST-LMM-Select included the first five PCs generated from Eigensoft by using total markers. Both MLMM and FarmCPU did not use PCs. The simulations were replicated 100 times. Power was examined under different levels of FDR and Type I error. All markers are sorted with the most significant one on top. A marker is claimed as false positive if no QTN is within a bilateral distance of 100,000 base pairs. For each threshold of FDR, power is defined as the proportion of QTNs detected (**left panel**). Similarly, markers without a QTN within 100,000 base pairs distance are used to derive the empirical null distribution of Type I error. For each threshold of Type I error, power is defined as the proportion of QTNs detected (**middle panel**). The **right panel** displays the counts of positive SNPs that passed a threshold of 1% after a Bonferroni multiple test correction. These positive SNPs are categorized into true positive and false positive. A positive SNP is true positive (solid filled) if a QTN is within a bilateral distance of 100,000 base pairs; otherwise, it is false positive (open shaded). In all three panels, PC/FaST-LMM-Select, MLMM, and FarmCPU are represented by blue, orange, and green colors, respectively.
