# Supplementary material for: Iterative Usage of Fixed and Random Effect Models for Powerful and Efficient Genome-Wide Association Studies
Source: PLoS Genet. 2016 Feb 1;12(2):e1005767. doi: 10.1371/journal.pgen.1005767 (PMC4734661; doi:10.1371/journal.pgen.1005767)
Supplement: S10 Fig — (DOCX) [file pgen.1005767.s010.docx]

**
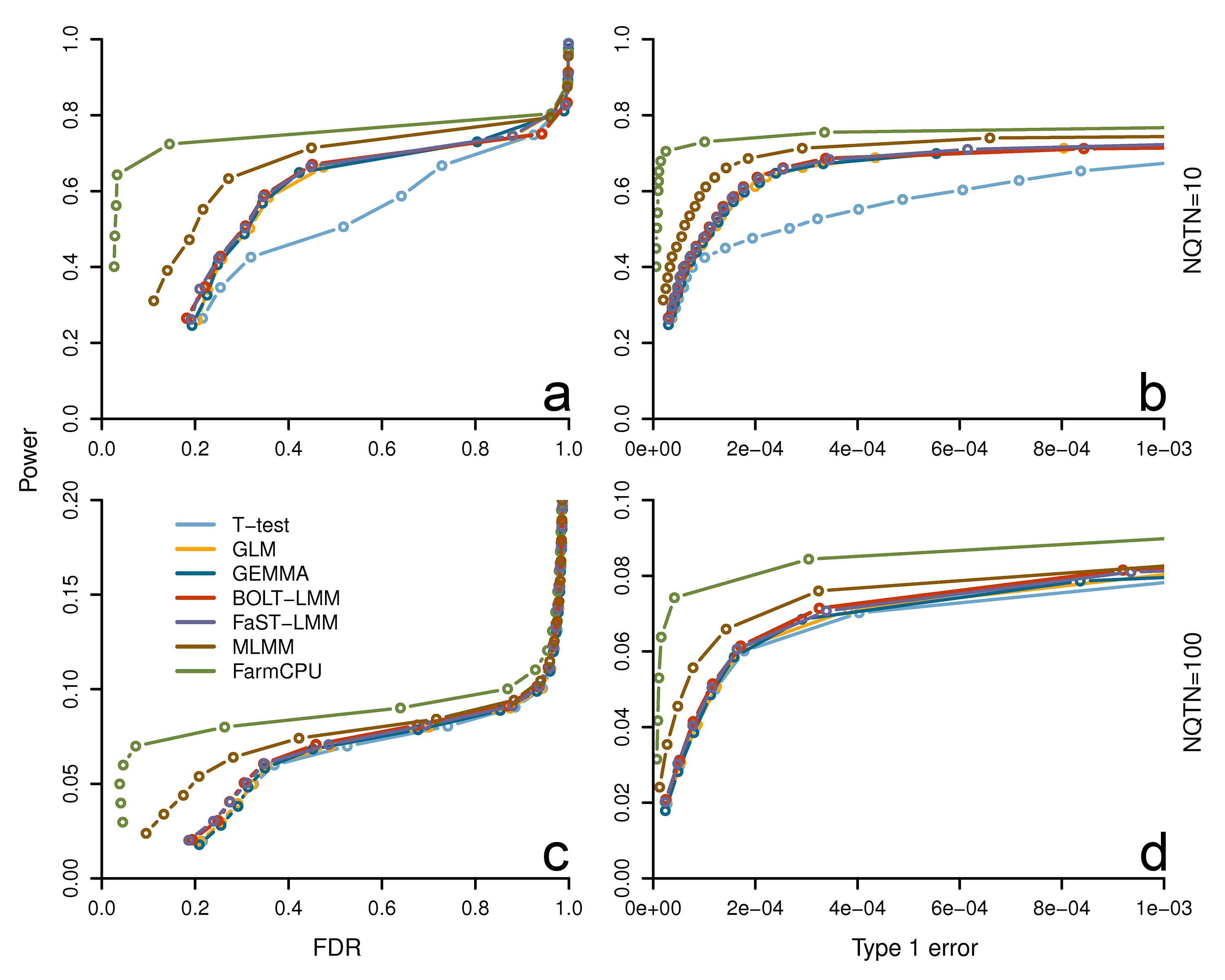
S10 Fig. Performances of Power versus FDR and Type I error.** Seven methods were performed to test Power/FDR and Power/Type I error in both simple trait and complex trait, including t-test, GLM, GEMMA, BOLT-LMM, FaST-LMM, MLMM and FarmCPU. The top panels **(a** and **b)** and bottom panels **(c** and **d)** display the performance in simple trait (controlled by 10 QTNs) and complex trait (controlled by 100 QTNs), respectively. The dataset is from a human population consists of 1,500 individuals genotyped with 495,473 SNPs. Additive genetic effects were simulated with 10 and 100 QTNs. The QTNs were randomly sampled from all the SNPs in each dataset. Residuals with normal distribution were added to the genetic effect to form phenotypes with heritability of 0.5. Power was examined under different levels of FDR and Type I error. A positive SNP is considered a true positive if a QTN is within a distance of 50,000 base pairs on either side, otherwise is considered a false positive. Power under different levels of FDR is displayed in subfigures **a** and **c**. Power under different levels of Type I error is displayed in subfigures **b** and **d**.
