# Supplementary material for: Iterative Usage of Fixed and Random Effect Models for Powerful and Efficient Genome-Wide Association Studies
Source: PLoS Genet. 2016 Feb 1;12(2):e1005767. doi: 10.1371/journal.pgen.1005767 (PMC4734661; doi:10.1371/journal.pgen.1005767)
Supplement: S12 Fig — (DOCX) [file pgen.1005767.s012.docx]

**
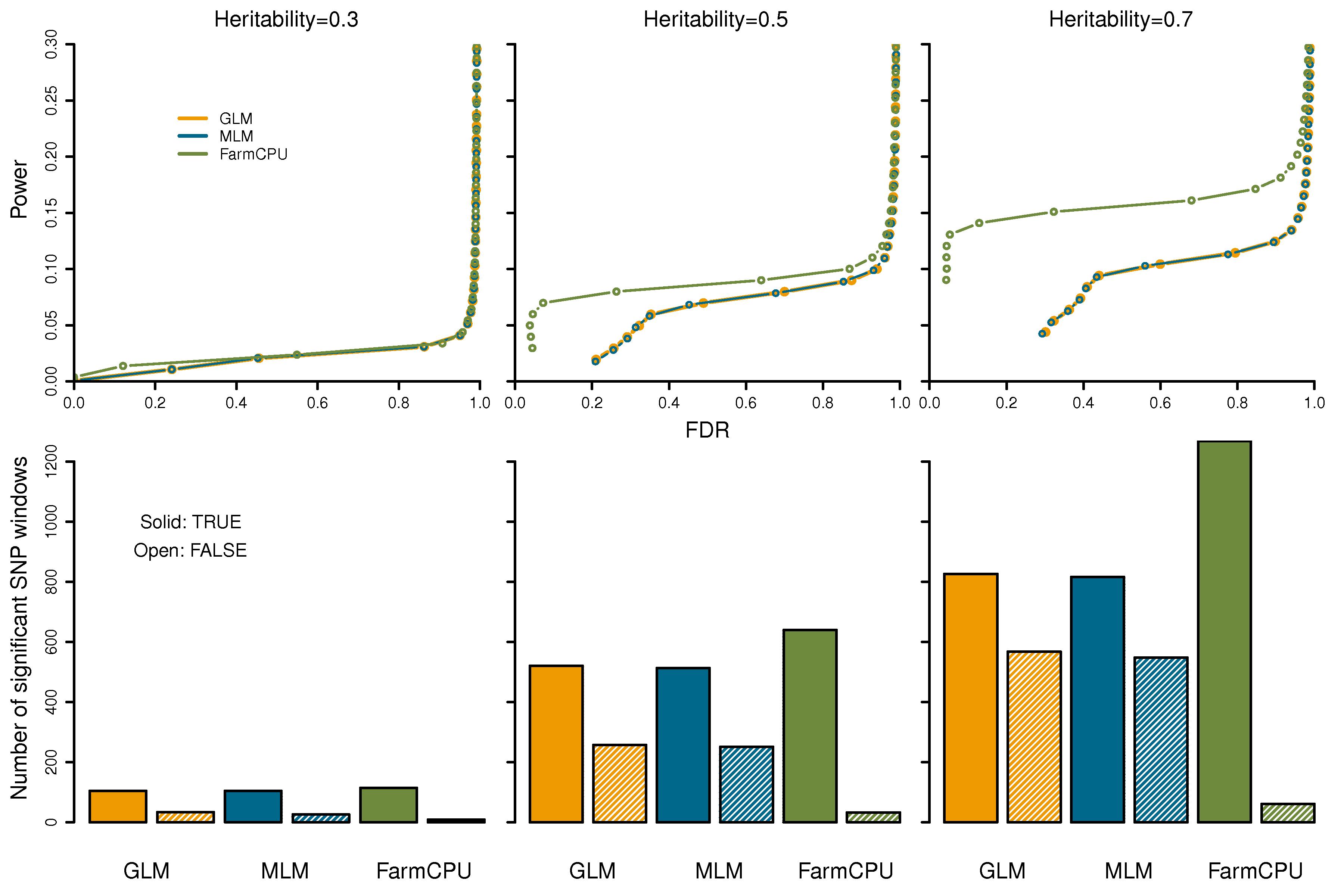
**

**S12 Fig. Performance of FarmCPU under different heritabilities.** FarmCPU is compared with both GLM and MLM. The comparisons were conducted for a simulated trait on WTCCC1 controls population. Additive genetic effects were simulated with 100 QTNs. The QTNs were randomly sampled from all the SNPs. Residuals with normal distribution were added to the genetic effect to form phenotypes with heritability of 0.3, 0.5, and 0.7. Both GLM and MLM included the first four PCs, derived from 10% of SNPs sampled randomly, as covariates to control population structure. FarmCPU did not use PCs. The simulations were replicated 100 times. The performances of the three methods are displayed in two ways. The top panels illustrate Power for a given FDR. A QTN was defined as detected when a SNP, within a bilateral distance of 50,000 base pairs, passed the corresponding threshold of FDR. Non-QTN SNPs were used to derive the empirical null distribution of FDR. The bottom panel displays the numbers of positive SNPs that passed a threshold of 1% after a Bonferroni multiple test correction. These positive SNPs are categorized into true positive and false positive SNPs. A positive SNP is a true positive (solid filled) if a QTN is within a 50,000 base pairs distance; otherwise, it is a false positive (open shaded). In both panels, GLM, MLM, and FarmCPU are represented by orange, blue, and green colors, respectively.
