# Supplementary material for: Iterative Usage of Fixed and Random Effect Models for Powerful and Efficient Genome-Wide Association Studies
Source: PLoS Genet. 2016 Feb 1;12(2):e1005767. doi: 10.1371/journal.pgen.1005767 (PMC4734661; doi:10.1371/journal.pgen.1005767)
Supplement: S13 Fig — (DOCX) [file pgen.1005767.s013.docx]

**
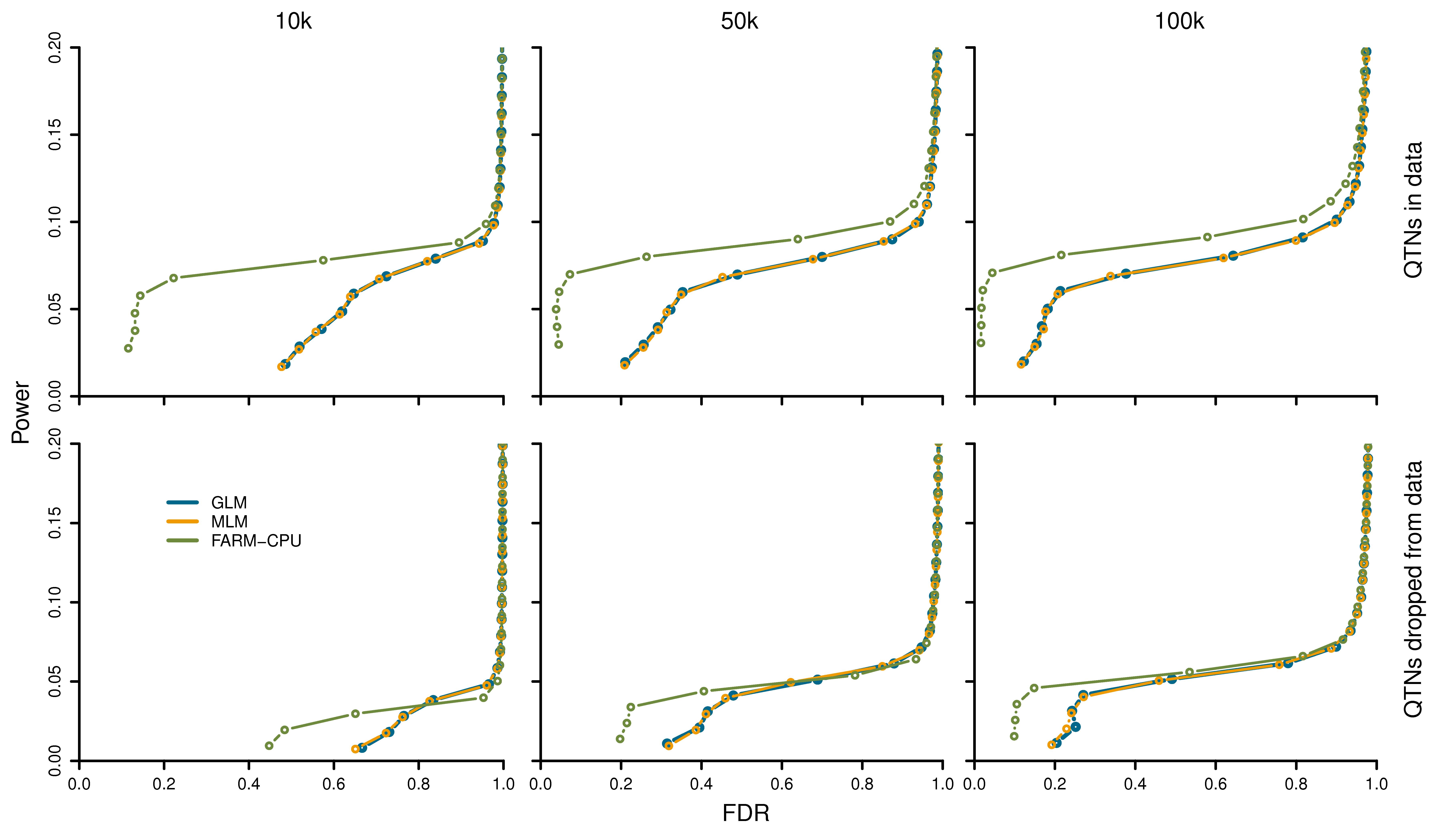
**

**S13 Fig. Performance of FarmCPU on markers that are inclusive or exclusive of QTNs.** FarmCPU is compared with both GLM and MLM. The comparisons were conducted for a simulated trait on WTCCC1 controls population. Additive genetic effects were simulated with 100 QTNs. The QTNs were randomly sampled from all the SNPs. Residuals with normal distribution were added to the genetic effect to form phenotypes with heritability of 0.5. Both GLM and MLM included the first four PCs, derived from 10% of SNPs sampled randomly, as covariates to control population structure. FarmCPU did not use PCs. The association tests between the simulated phenotypes and genetic markers were conducted in two ways. First, the QTNs were included in the testing markers (top panel). Second, the QTNs were excluded from the testing markers (bottom panel). The simulations were replicated 100 times. Power for a given FDR was compared with the three methods. A QTN was defined as detected when a SNP, within a bilateral distance of 10,000, 50,000, or 100,000 base pairs, passed the corresponding threshold of FDR. Non-QTN SNPs were used to derive the empirical null distribution of FDR. GLM, MLM, and FarmCPU are represented by orange, blue and green colors, respectively.
