# Supplementary material for: Iterative Usage of Fixed and Random Effect Models for Powerful and Efficient Genome-Wide Association Studies
Source: PLoS Genet. 2016 Feb 1;12(2):e1005767. doi: 10.1371/journal.pgen.1005767 (PMC4734661; doi:10.1371/journal.pgen.1005767)
Supplement: S14 Fig — (DOCX) [file pgen.1005767.s014.docx]

**
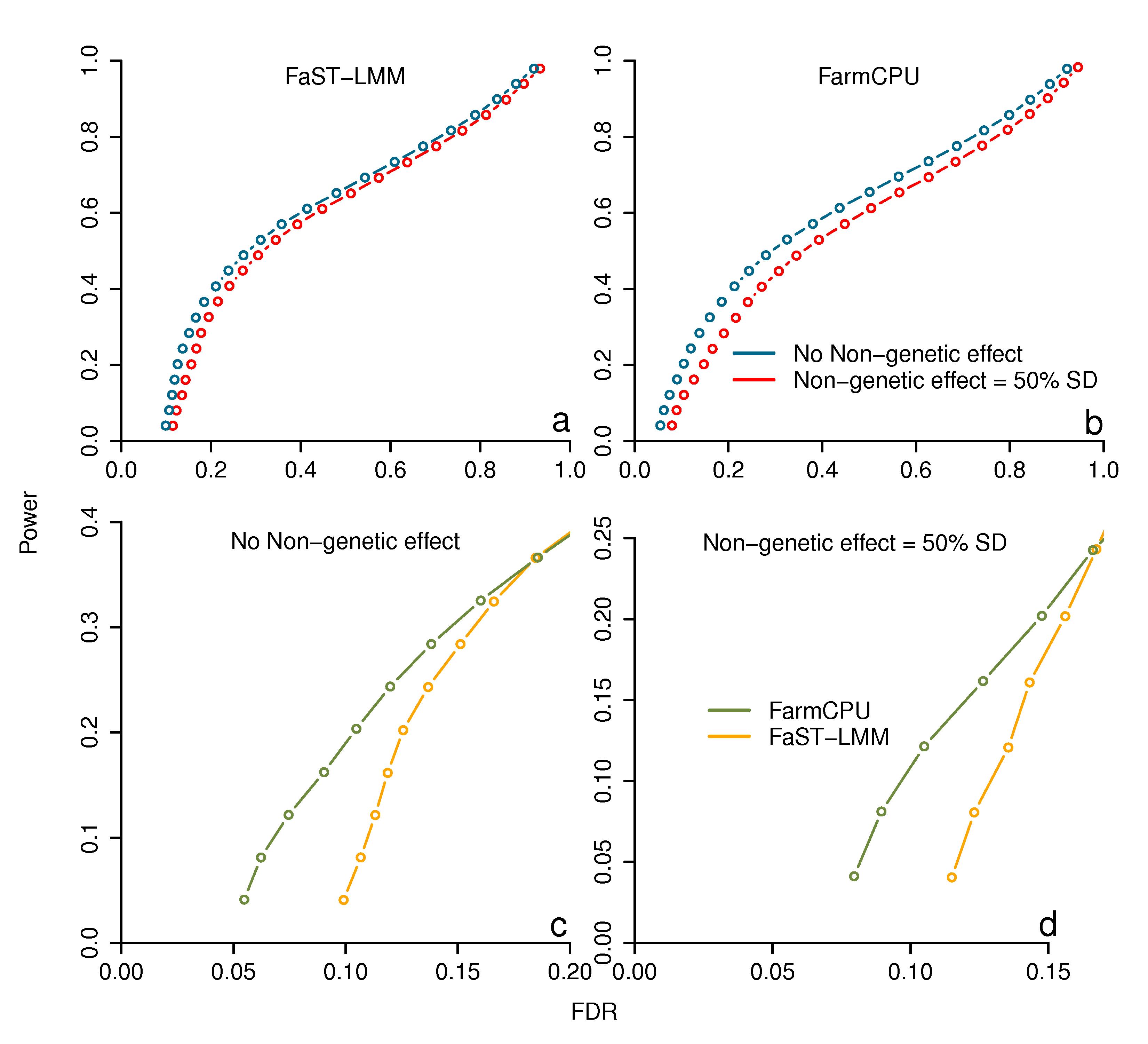
**

**S14 Fig. Power and FDR of MLM and FarmCPU in simulated phenotypes with non-genetic effects.** Two methods were performed in this study including MLM (performed by FaST-LMM) and FarmCPU. The genotype data is from East Asian lung cancer data set. Additive genetic effects were simulated with 500 QTNs and each QTN has the same effect. Residuals with normal distribution were added to the genetic effect to form phenotypes with heritability of 0.75. We further added the environmental effects from the three populations (China, Japan, and Korea) by adding an effect that aligned with subpopulation indicators that explained an additional 50% standard deviation (SD) of the phenotype. Both with and without non-genetic effects were tested and the simulations were replicated 100 times. MLM includes PCs while FarmCPU not. Power was examined under different levels of FDR. All markers are sorted with the most significant one on top. A marker is claimed as false positive if no QTN is within a bilateral distance of 100,000 base pairs. For each threshold of FDR, Power is defined as the proportion of QTNs detected (**a-d**). Performances in with and without non-genetic effect of the FaST-LMM and FarmCPU methods are shown in top panel (**a** and **b**). Performances of different methods in with and without non-genetic effect are shown in bottom panel (**c** and **d**). FarmCPU over performed FaST-LMM in both two cases.
