# Supplementary material for: Iterative Usage of Fixed and Random Effect Models for Powerful and Efficient Genome-Wide Association Studies
Source: PLoS Genet. 2016 Feb 1;12(2):e1005767. doi: 10.1371/journal.pgen.1005767 (PMC4734661; doi:10.1371/journal.pgen.1005767)
Supplement: S16 Fig — (DOCX) [file pgen.1005767.s016.docx]

**
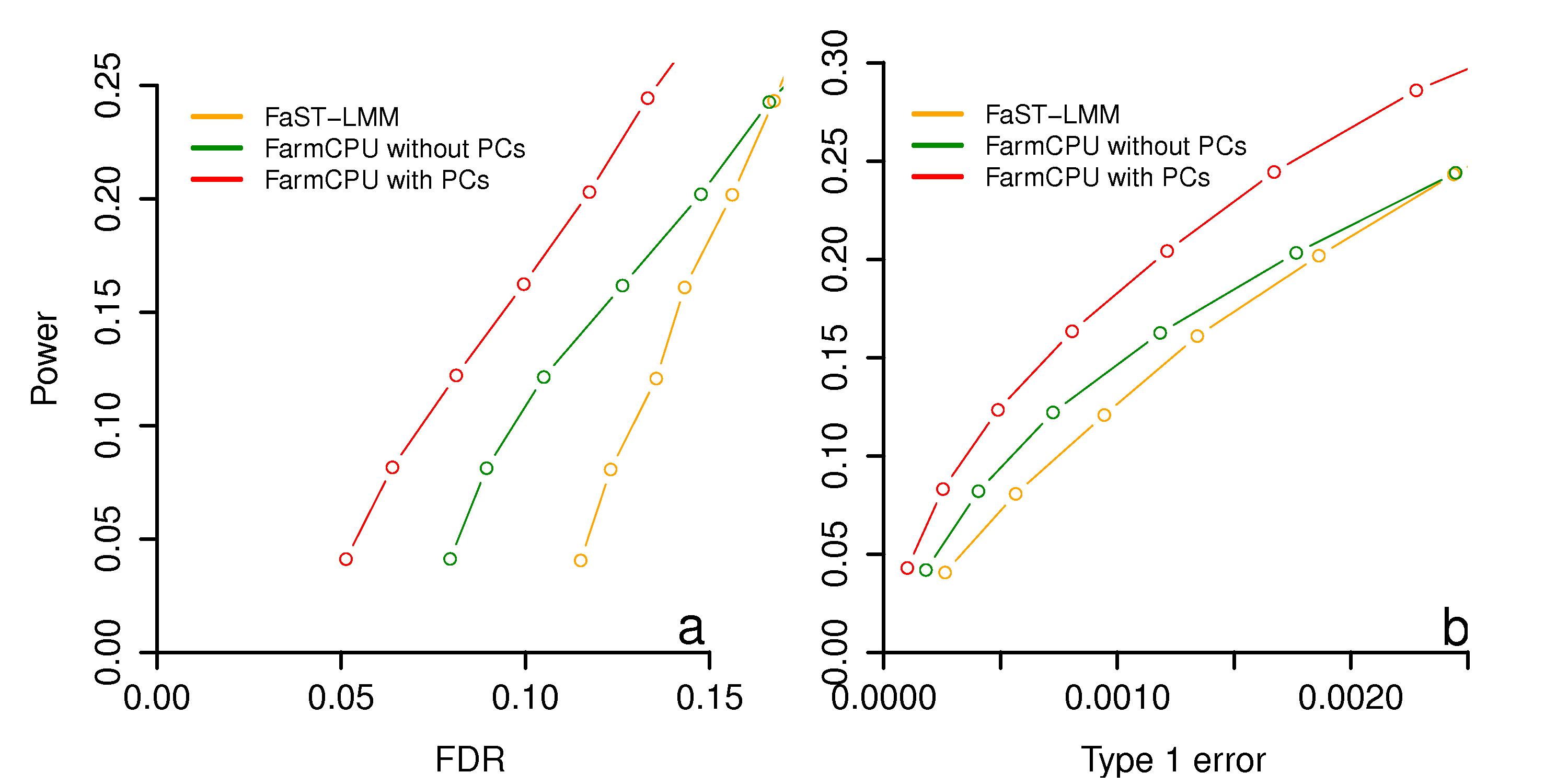
**

**S16 Fig. Effect of PCs in FarmCPU using simulated phenotypes that include non-genetic effects.** Two methods were performed in this study including MLM (performed by FaST-LMM) and FarmCPU. MLM include PCs and FarmCPU was tested in two situations: without PCs and include PCs. The genotype data is from East Asian lung cancer data set. Additive genetic effects were simulated with 500 QTNs and each QTN has the same effect. Residuals with normal distribution were added to the genetic effect to form phenotypes with heritability of 0.75. We further added the environmental effects from the three populations (China, Japan, and Korea) by adding an effect that aligned with subpopulation indicators that explained an additional 25% of the phenotype variance. The simulations were replicated 100 times. Power was examined under different levels of FDR and Type I error. All markers are sorted with the most significant one on top. A marker is claimed as false positive if no QTN is within a bilateral distance of 100,000 base pairs. For each threshold of FDR, Power is defined as the proportion of QTNs detected (**a**). Similarly, markers without a QTN within 100,000 base pairs distance are used to derive the empirical null distribution of Type I error. For each threshold of Type I error, Power is defined as the proportion of QTNs detected (**b**). The results shown that include PCs in FarmCPU will increase the Power when non-genetic effects are included in phenotype and correlated with population structure.
