# Supplementary material for: Iterative Usage of Fixed and Random Effect Models for Powerful and Efficient Genome-Wide Association Studies
Source: PLoS Genet. 2016 Feb 1;12(2):e1005767. doi: 10.1371/journal.pgen.1005767 (PMC4734661; doi:10.1371/journal.pgen.1005767)
Supplement: S17 Fig — (DOCX) [file pgen.1005767.s017.docx]

**
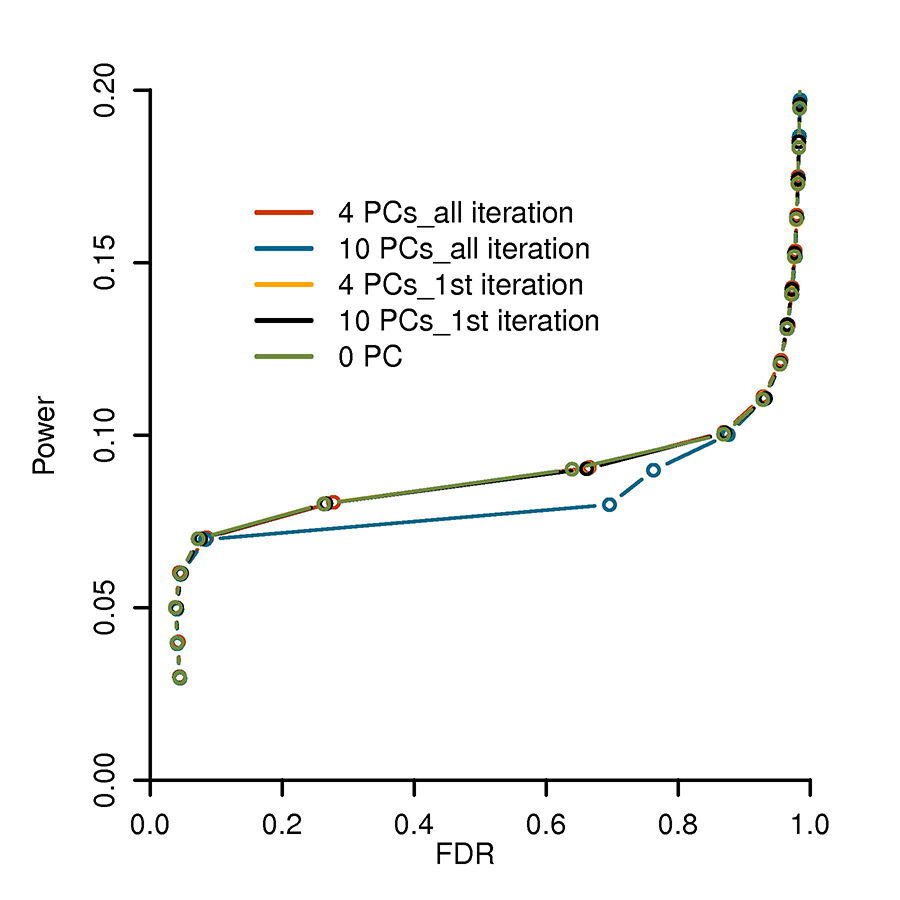
**

**S17 Fig. Effect of including population structure as covariates in FarmCPU.** The effect was evaluated in WTCCC1 controls population. Population structure was calculated as PCs, derived from 10% of SNPs sampled randomly. Simulated phenotypes were used to examine Power and FDR when fitting PCs as covariates in FarmCPU. The simulated trait was controlled by 100 QTNs with heritability of 0.5. The 100 QTNs were sampled from all the SNPs. Different numbers of PCs (4 and 10) were fitted in FarmCPU in two ways. First, PCs were fit in the fixed effect model for testing markers in the first iteration between the fixed and random effect models. Second, the PCs were fit in the fixed effect model for all the iterations between the fixed and random effect models. The results indicated that FarmCPU was tolerant to fitting PCs in both cases, unless the number of PCs was too big (e.g., 10).
