# Supplementary material for: Iterative Usage of Fixed and Random Effect Models for Powerful and Efficient Genome-Wide Association Studies
Source: PLoS Genet. 2016 Feb 1;12(2):e1005767. doi: 10.1371/journal.pgen.1005767 (PMC4734661; doi:10.1371/journal.pgen.1005767)
Supplement: S18 Fig — (DOCX) [file pgen.1005767.s018.docx]

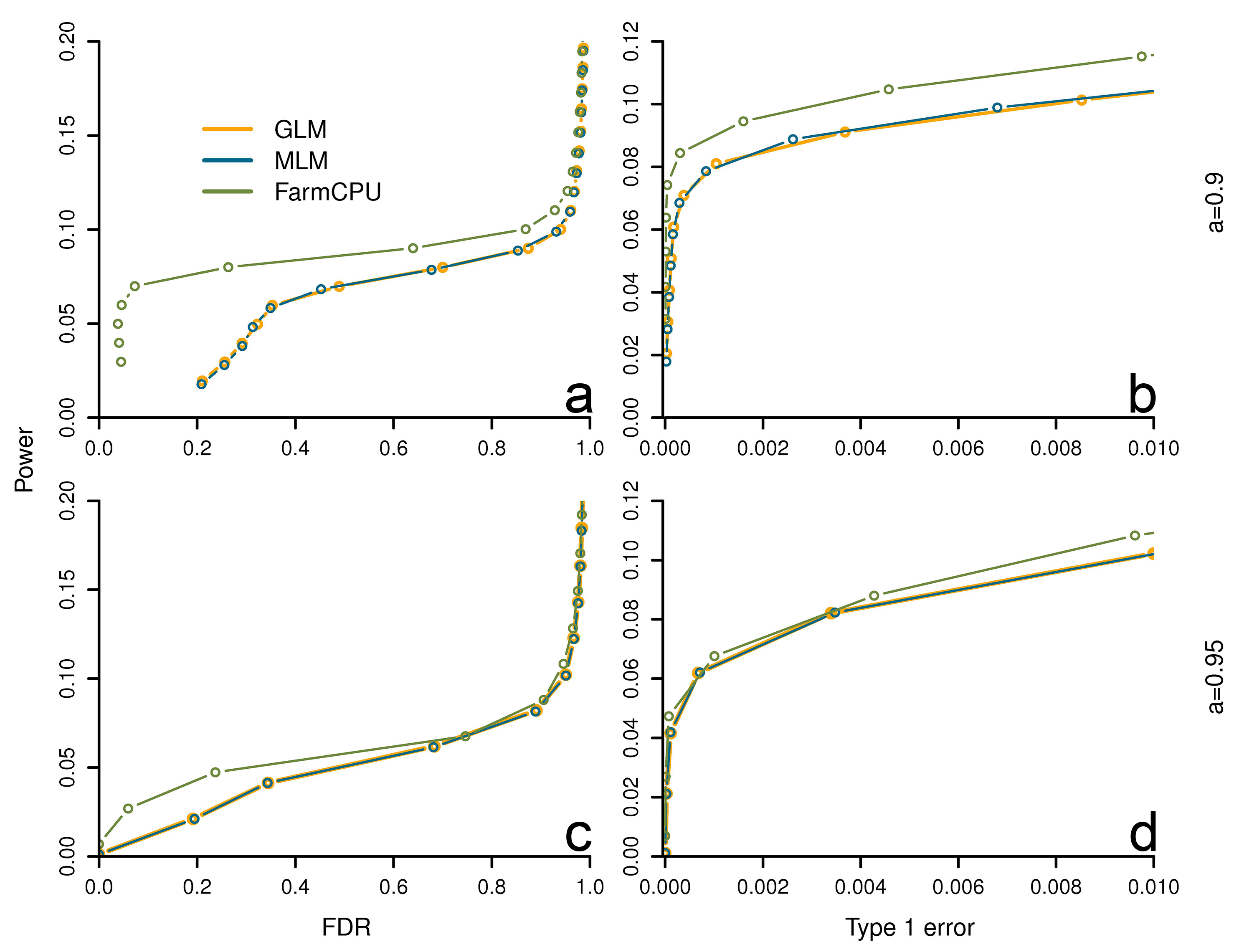
**S18 Fig. Performances of Power against False Discovery Rate and Type I error in different QTN effect size.** Three methods were performed in this study including GLM, MLM, and FarmCPU. The genotype data is from WTCCC1 controls population. Additive genetic effects were simulated with 100 QTNs. The QTN effects followed a geometric distribution with additive effect of parameter *a*. The effect of the i^th^ QTN was *a^i^*. Two levels of *a* were tested include 0.9 (**a**, **b**) and 0.95 (**c**, **d**). The QTNs were randomly sampled from all the SNPs in each dataset. Residuals with normal distribution were added to the genetic effect to form phenotypes with heritability of 0.5. Power was examined under different levels of FDR and Type I error. A positive SNP is considered a true positive if a QTN is within a distance of 50,000 base pairs on either side, otherwise is considered a false positive. Power under different levels of FDR is displayed in subfigures **a** and **c**. Power under different levels of Type I error is displayed in subfigures **b** and **d**.
