# Supplementary material for: Iterative Usage of Fixed and Random Effect Models for Powerful and Efficient Genome-Wide Association Studies
Source: PLoS Genet. 2016 Feb 1;12(2):e1005767. doi: 10.1371/journal.pgen.1005767 (PMC4734661; doi:10.1371/journal.pgen.1005767)
Supplement: S19 Fig — (DOCX) [file pgen.1005767.s019.docx]

**
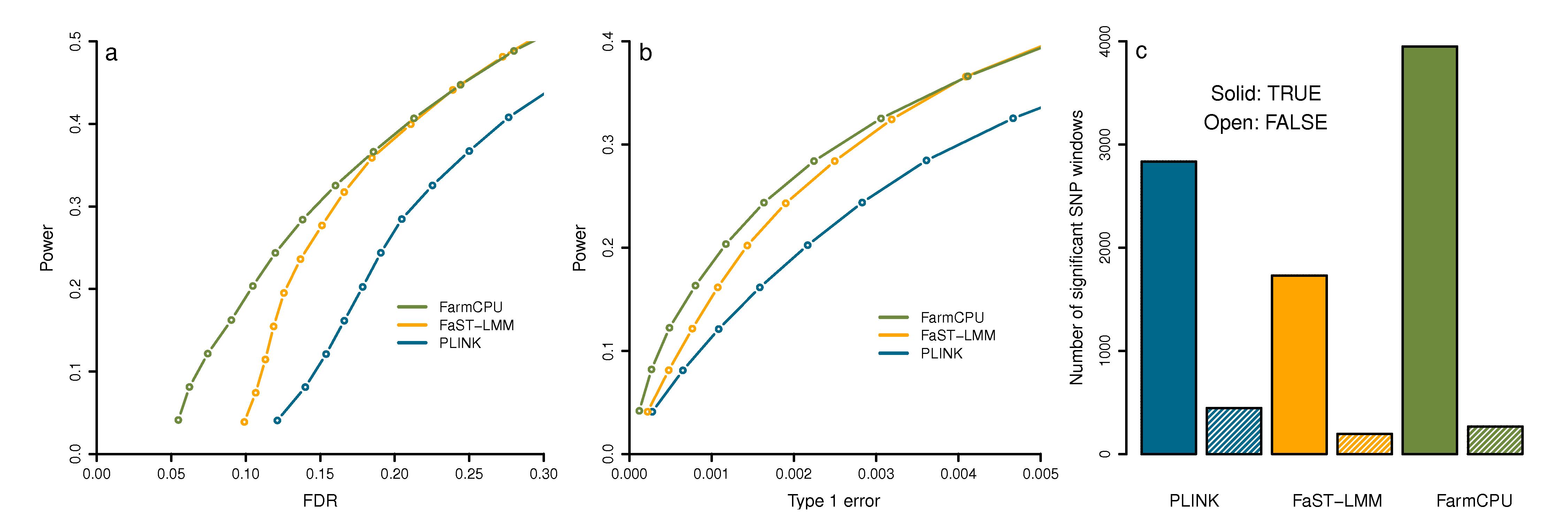
S19 Fig. Performances of Power against False Discovery Rate and Type I error in complex traits.** Three methods were performed in this study including GLM (performed by PLINK), MLM (performed by FaST-LMM), and FarmCPU. The genotype data is from East Asian lung cancer data set. Additive genetic effects were simulated with 500 QTNs and each QTN has the same effect. The QTNs were randomly sampled from the SNPs located in first 10 Chromosomes. Residuals with normal distribution were added to the genetic effect to form phenotypes with heritability of 0.75. Both PLINK and FaST-LMM include the top four PCs in the model while FarmCPU didn’t. The simulations were replicated 100 times. Power was examined under different levels of FDR and Type I error. All markers are sorted with the most significant one on top. A marker is claimed as false positive if no QTN is within a bilateral distance of 100,000 base pairs. For each threshold of FDR, power is defined as the proportion of QTNs detected (**a**). Similarly, markers without a QTN within 100,000 base pairs distance are used to derive the empirical null distribution of Type I error. For each threshold of Type I error, power is defined as the proportion of QTNs detected (**b**). The **c panel** displays the counts of positive SNPs that passed a threshold of 1% after a Bonferroni multiple test correction. These positive SNPs are categorized into true positive and false positive. A positive SNP is true positive (solid filled) if a QTN is within a bilateral distance of 100,000 base pairs; otherwise, it is false positive (open shaded). In all three panels, PLINK, FaST-LMM, and FarmCPU are represented by blue, orange, and green colors, respectively.
