# Supplementary material for: Iterative Usage of Fixed and Random Effect Models for Powerful and Efficient Genome-Wide Association Studies
Source: PLoS Genet. 2016 Feb 1;12(2):e1005767. doi: 10.1371/journal.pgen.1005767 (PMC4734661; doi:10.1371/journal.pgen.1005767)
Supplement: S20 Fig — (DOCX) [file pgen.1005767.s020.docx]

**
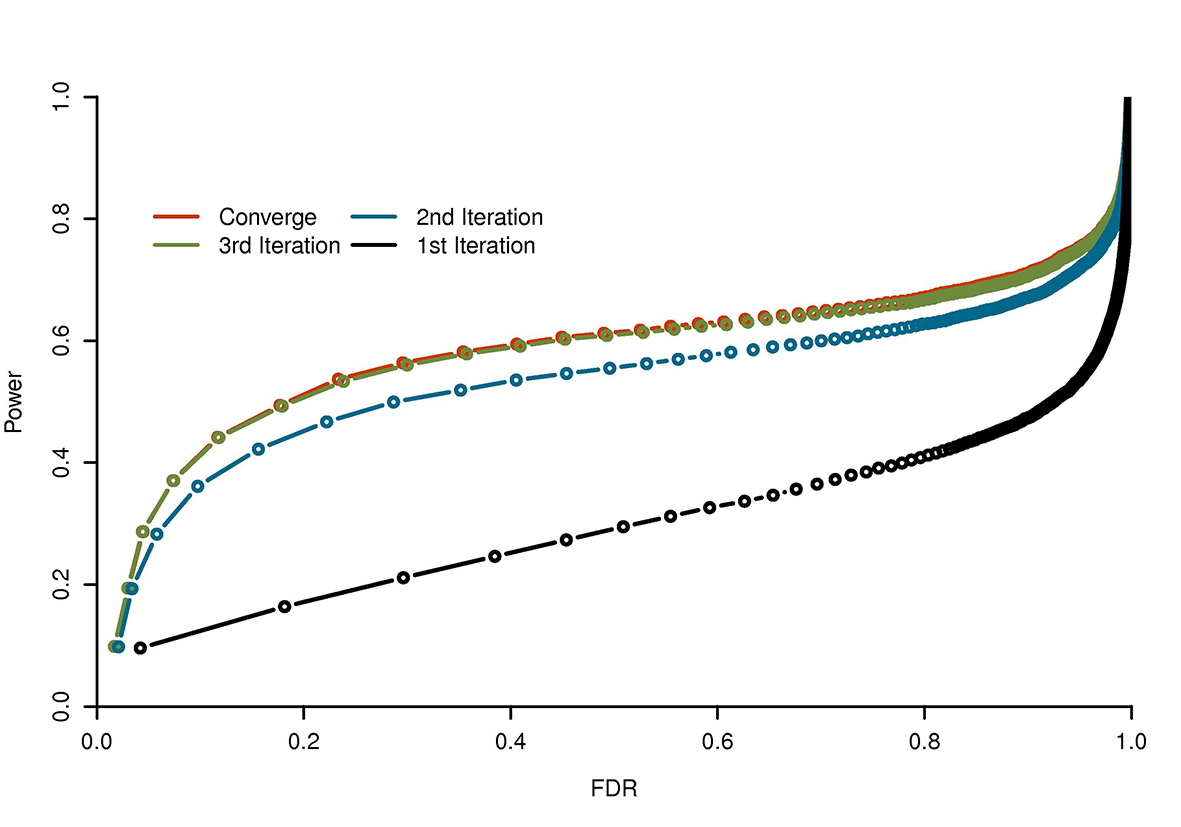
S20 Fig. The impact of iterations on FarmCPU.** The impact was evaluated by pairwise Power and FDR on simulated phenotypes controlled by 10 QTNs with heritability of 50%. These QTNs were randomly sampled from real genotypes of 1,178 *Arabidopsis thaliana* individuals with 214,545 SNP markers. Iteration refers to testing genetic markers, one at a time, in a fixed model with pseudo QTNs as covariates; updating pseudo QTNs in a random model; then returning to the fixed model to repeat the sequence over and over again. Power and FDR were examined at the first, second, third iterations, and finally, at the iteration when pseudo QTNs converged. The simulations were replicated 1,000 times. A marker is claimed as false positive if no QTN is within a bilateral distance of 10,000 base pairs. The average Power and FDR are displayed.
