# Supplementary material for: Iterative Usage of Fixed and Random Effect Models for Powerful and Efficient Genome-Wide Association Studies
Source: PLoS Genet. 2016 Feb 1;12(2):e1005767. doi: 10.1371/journal.pgen.1005767 (PMC4734661; doi:10.1371/journal.pgen.1005767)
Supplement: S21 Fig — (DOCX) [file pgen.1005767.s021.docx]

**
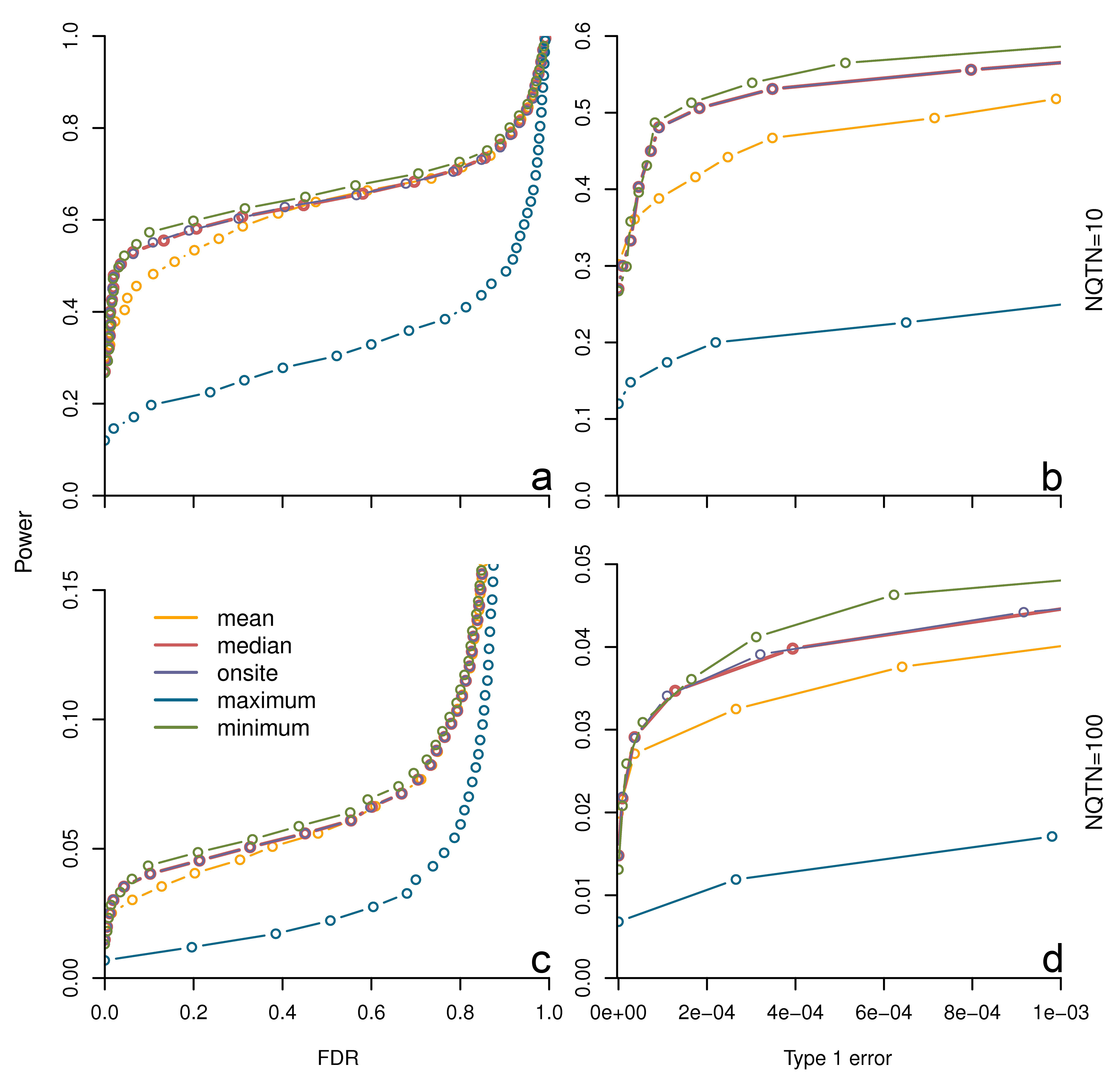
**

**S21 Fig. Impact of substitution methods on P values of pseudo QTNs.** The impact was evaluated by pairing Power versus FDR and Type I error. Five substitution methods were examined, including onsite, mean, minimum, median, and maximum. Onsite refers to the same method used by the MLMM, which reports P values of pseudo QTNs from the model that includes only the pseudo QTNs, without the testing markers. The other methods use the mean, minimum, median, and maximum P value of each pseudo QTN when examined together with all markers, one at a time. Both simple phenotypes controlled by 10 QTNs and complex phenotypes controlled by 100 QTNs are simulated to examined Power/FDR and Power/Type I error. The QTNs were randomly sampled from genotypes of 1,178 *Arabidopsis* *thaliana* individuals with 214,545 SNP markers. A marker is claimed as false positive if no QTN is within a bilateral distance of 50,000 base pairs. The simulations were replicated 100 times and the averages of Power versus FDR and Type I error are displayed. The results indicate that the minimum method performs best.
