# Supplementary material for: Iterative Usage of Fixed and Random Effect Models for Powerful and Efficient Genome-Wide Association Studies
Source: PLoS Genet. 2016 Feb 1;12(2):e1005767. doi: 10.1371/journal.pgen.1005767 (PMC4734661; doi:10.1371/journal.pgen.1005767)
Supplement: S22 Fig — (DOCX) [file pgen.1005767.s022.docx]

**
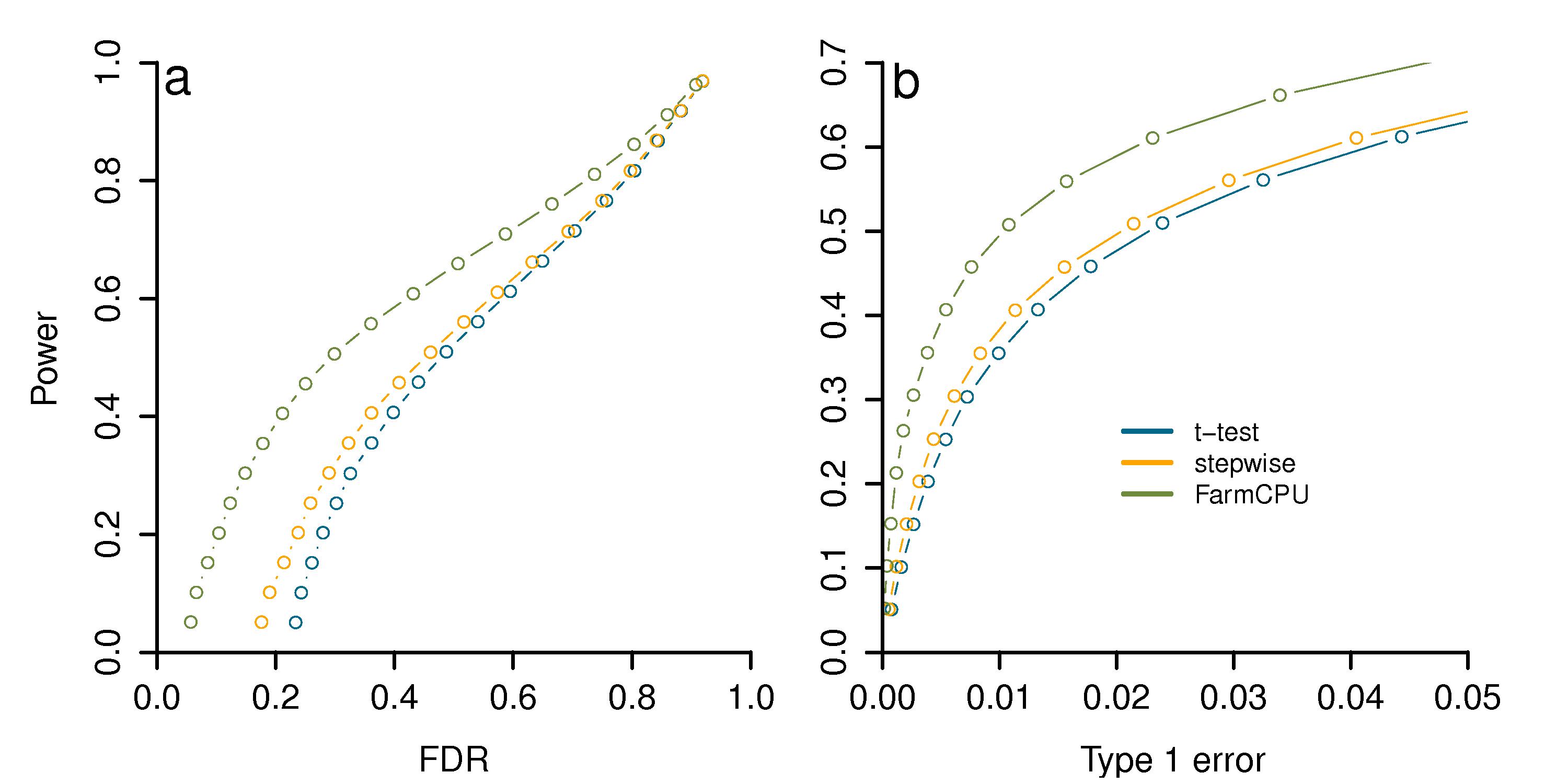
**

**S22 Fig. Power versus FDR and Type I error in three methods.** Three methods were t-test, stepwise regression (stepwise) and FarmCPU. Both stepwise regression and FarmCPU use a stepwise fashion. The stepwise regression starts from t-test and use BIC (Bayesian Information Criterion) to select pseudo QTNs as fixed effects while FarmCPU use a combination of bin method, substitution process and random effect model. The genotype data is from East Asian lung cancer data set. Additive genetic effects were simulated with 500 Quantitative Trait Nucleotide (QTNs) and each QTN has the same effect. Residuals with normal distribution were added to the genetic effect to form phenotypes with heritability of 0.75. The simulations were replicated 100 times. Power was examined under different levels of FDR and Type I error. All markers are sorted with the most significant one on top. A marker is claimed as false positive if no QTN is within a bilateral distance of 100,000 base pairs. For each threshold of FDR, Power is defined as the proportion of QTNs detected (**a**). Similarly, markers without a QTN within 100,000 base pairs distance are used to derive the empirical null distribution of Type I error. For each threshold of Type I error, Power is defined as the proportion of QTNs detected (**b**). Stepwise regression does increase the Power (stepwise performs better than t-test) and a combination of bin method, substitution process and random effect model make more gains on the Power respect to both FDR and Type I error.
