# Supplementary material for: Iterative Usage of Fixed and Random Effect Models for Powerful and Efficient Genome-Wide Association Studies
Source: PLoS Genet. 2016 Feb 1;12(2):e1005767. doi: 10.1371/journal.pgen.1005767 (PMC4734661; doi:10.1371/journal.pgen.1005767)
Supplement: S23 Fig — (DOCX) [file pgen.1005767.s023.docx]

**
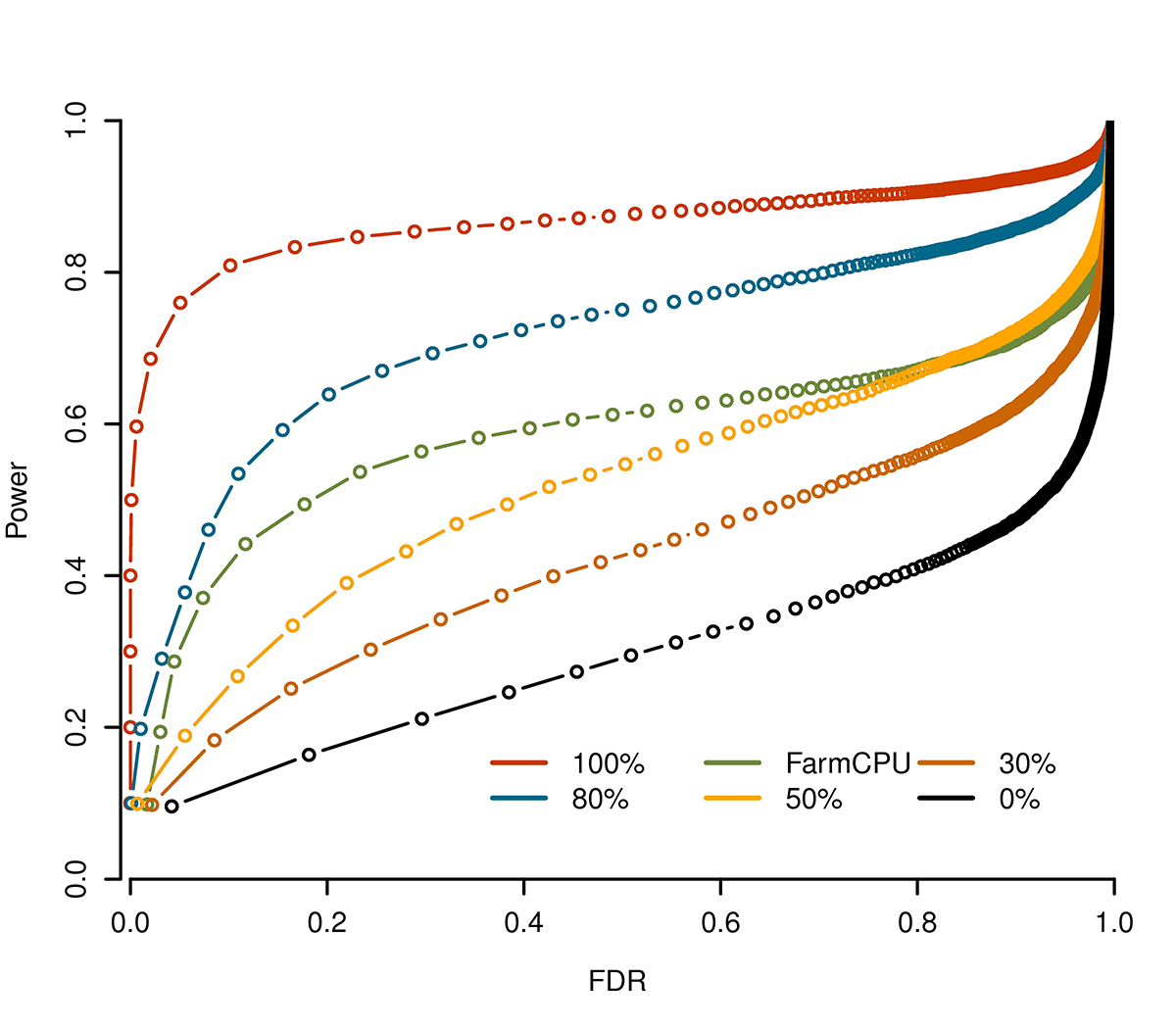
S23 Fig. Comparison of FarmCPU and GLM with varying levels of prior knowledge.** The comparisons were performed on simulated phenotypes controlled by 10 QTNs with heritability of 50%. These QTNs were randomly sampled from real genotypes of 1,178 *Arabidopsis thaliana* individuals with 214,545 SNP markers. Different proportions of QTNs were fitted as covariates in GLM as prior knowledge. FarmCPU did not use the prior knowledge. The comparisons are based on Power and FDR. Power and FDR were examined in pairs. The simulations were replicated 1,000 times. A marker is claimed as false positive if no QTN is within a bilateral distance of 10,000 base pairs. The averages of Power and FDR are displayed. The higher the proportion of true QTNs incorporated in GLM, the higher the Power for a given level of FDR. Interestingly, FarmCPU's Power with no prior knowledge is equivalent to a GLM with more than 50% known true QTNs incorporated.
