# Supplementary material for: Iterative Usage of Fixed and Random Effect Models for Powerful and Efficient Genome-Wide Association Studies
Source: PLoS Genet. 2016 Feb 1;12(2):e1005767. doi: 10.1371/journal.pgen.1005767 (PMC4734661; doi:10.1371/journal.pgen.1005767)
Supplement: S24 Fig — (DOCX) [file pgen.1005767.s024.docx]

**
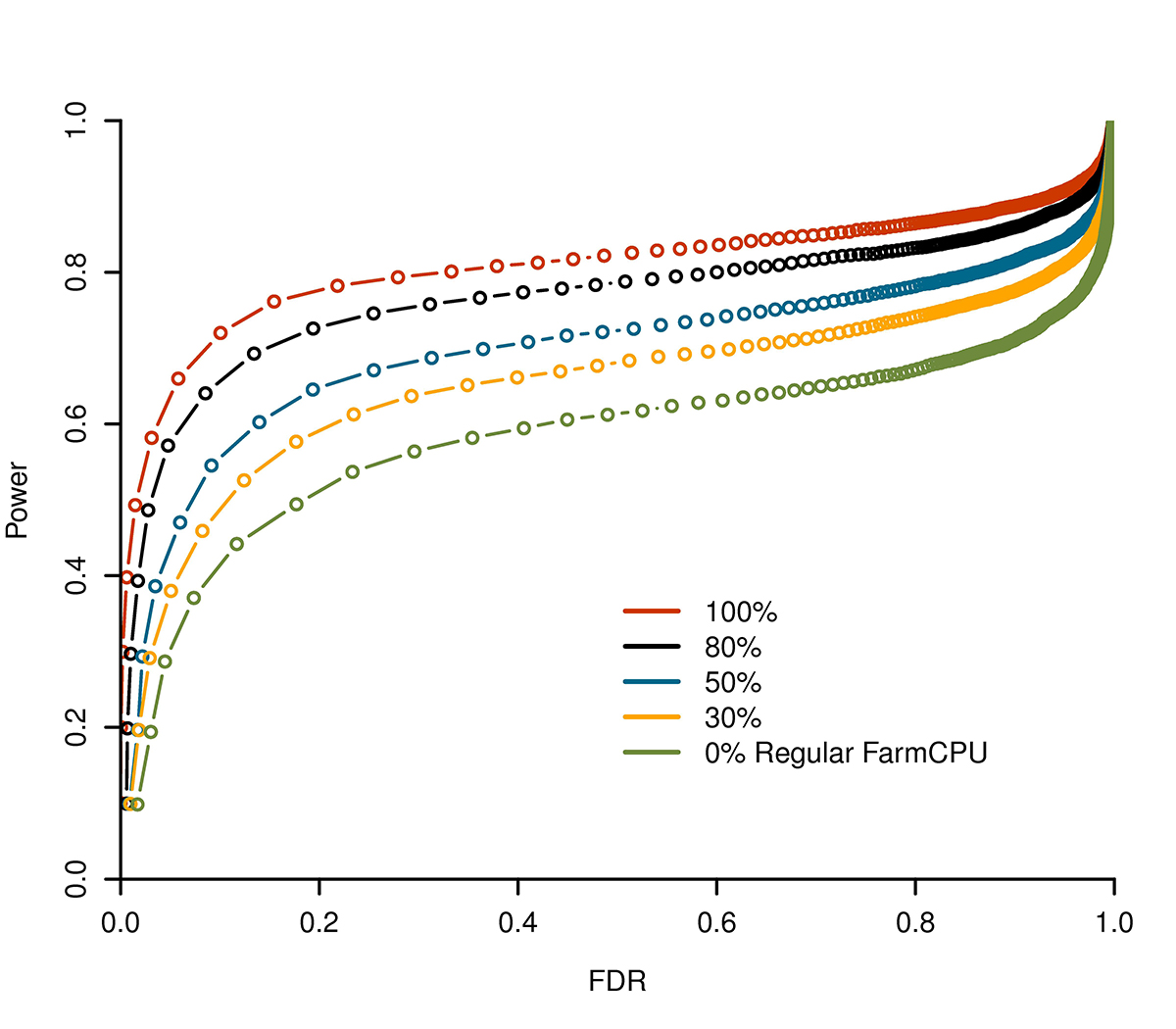
S24 Fig. Impact of prior knowledge on the Power of FarmCPU.** The examinations were performed on simulated phenotypes controlled by 10 QTNs with heritability of 50%. These QTNs were randomly selected from real genotypes of 1,178 *Arabidopsis* *thaliana* individuals with 214,545 SNP markers. Different proportions (0%, 30%, 50%, 80%, and 100%) of QTNs were fitted as covariates in FarmCPU as prior knowledge. The Power and FDR were examined in pairs. The simulations were replicated 1,000 times. A marker is claimed as false positive if no QTN is within a bilateral distance of 10,000 base pairs. The averages of Power and FDR are displayed. FarmCPU has higher Power when more QTNs are fitted as covariates for a given level of FDR.
