# Supplementary material for: Iterative Usage of Fixed and Random Effect Models for Powerful and Efficient Genome-Wide Association Studies
Source: PLoS Genet. 2016 Feb 1;12(2):e1005767. doi: 10.1371/journal.pgen.1005767 (PMC4734661; doi:10.1371/journal.pgen.1005767)
Supplement: S25 Fig — (DOCX) [file pgen.1005767.s025.docx]

**
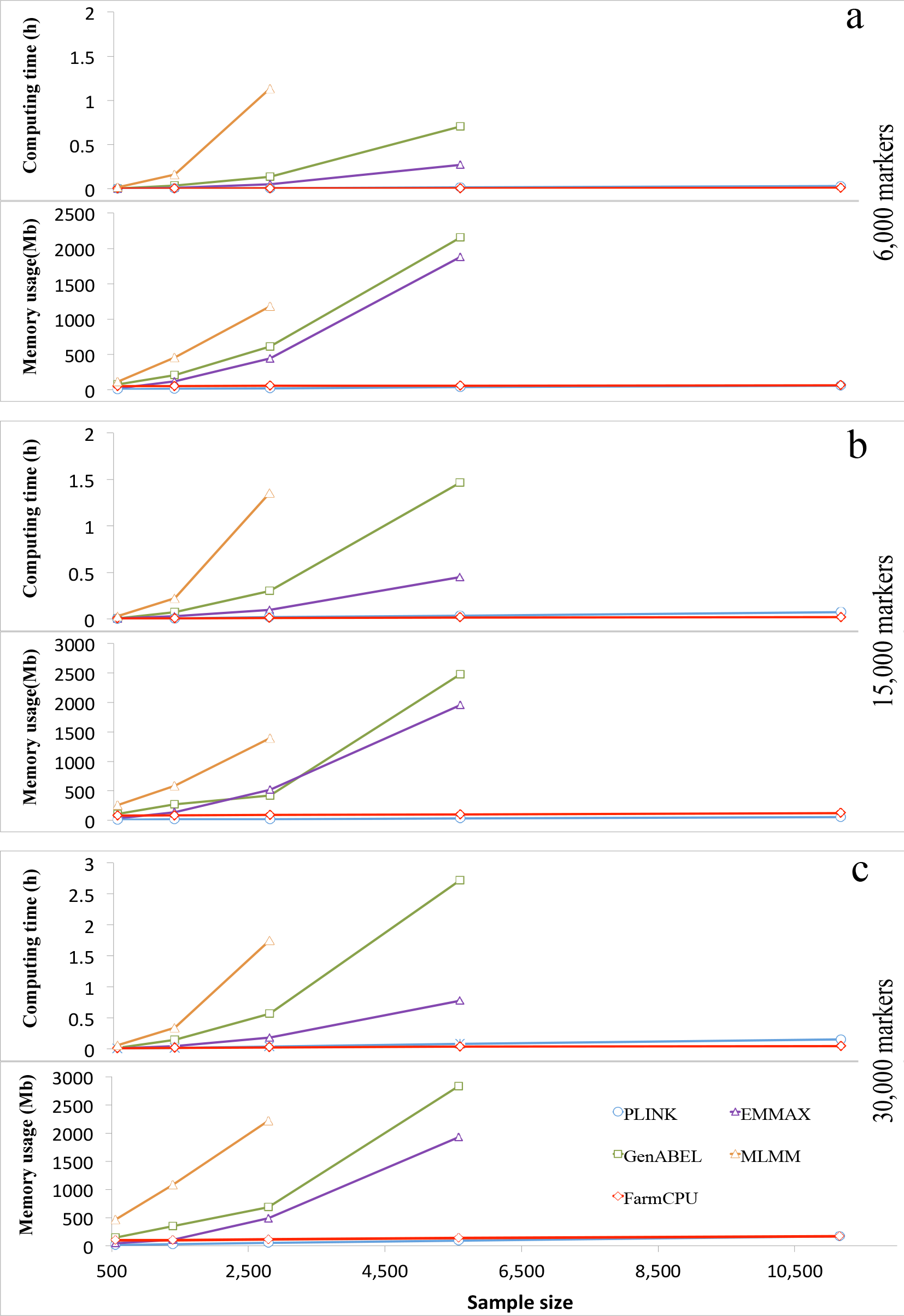
**

**S25 Fig. Comparison of computing time and memory usage among five software packages.** These packages are PLINK, EMMAX, GenABEL, MLMM, and FarmCPU. The GLM was performed by PLINK. MLMs were performed by EMMAX, GenABEL, and MLMM. The analyses were performed on a laptop (Asus A53S) running a Linux system (Ubuntu 12.10, 64 bit) with a 4.0Gb of random-access memory (RAM) and an Inter duo Core i3-2310M processor at 2.1 GHz. One core was used for this test. Three levels of total number of markers were tested: **(a)** 6,000, **(b)** 15,000, and **(c)** 30,000. The last data point coincides with the maximum sample size (n) that each software package could process without freezing the computer, except PLINK and FarmCPU. The limitations for these two software packages were not reached with the maximum n tested.
