# Supplementary material for: Iterative Usage of Fixed and Random Effect Models for Powerful and Efficient Genome-Wide Association Studies
Source: PLoS Genet. 2016 Feb 1;12(2):e1005767. doi: 10.1371/journal.pgen.1005767 (PMC4734661; doi:10.1371/journal.pgen.1005767)
Supplement: S26 Fig — (DOCX) [file pgen.1005767.s026.docx]

**
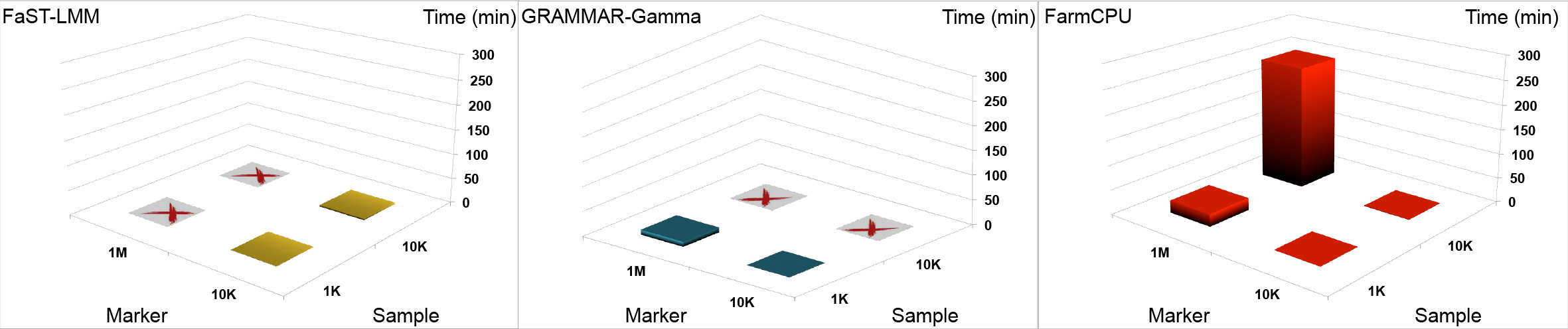
**

**S26 Fig. Computing times for analyzing datasets with large numbers of individuals and large numbers of markers.** The computing time of FarmCPU (R Package) is compared with the computing times of two commonly used software packages, FaST-LMM (FaST-LMM software, version 2.07) and GRAMMAR-Gamma (GenABEL software, version 1.8-0). The analyses were performed on a laptop (Asus A53S) running a Linux system (Ubuntu 12.10, 64 bit), with 4.0Gb of Random-Access Memory (RAM) and an Intel duo Core i3-2310M processer at 2.1 GHz. One core was used to analyze datasets with various combinations of two sample sizes (1,000 and 10,000) and two marker densities (10,000 and 1,000,000). Combinations labeled “X” indicate when a software package was unable to process the dataset due to computer freezing.
