# Supplementary material for: Iterative Usage of Fixed and Random Effect Models for Powerful and Efficient Genome-Wide Association Studies
Source: PLoS Genet. 2016 Feb 1;12(2):e1005767. doi: 10.1371/journal.pgen.1005767 (PMC4734661; doi:10.1371/journal.pgen.1005767)
Supplement: S27 Fig — (DOCX) [file pgen.1005767.s027.docx]

**
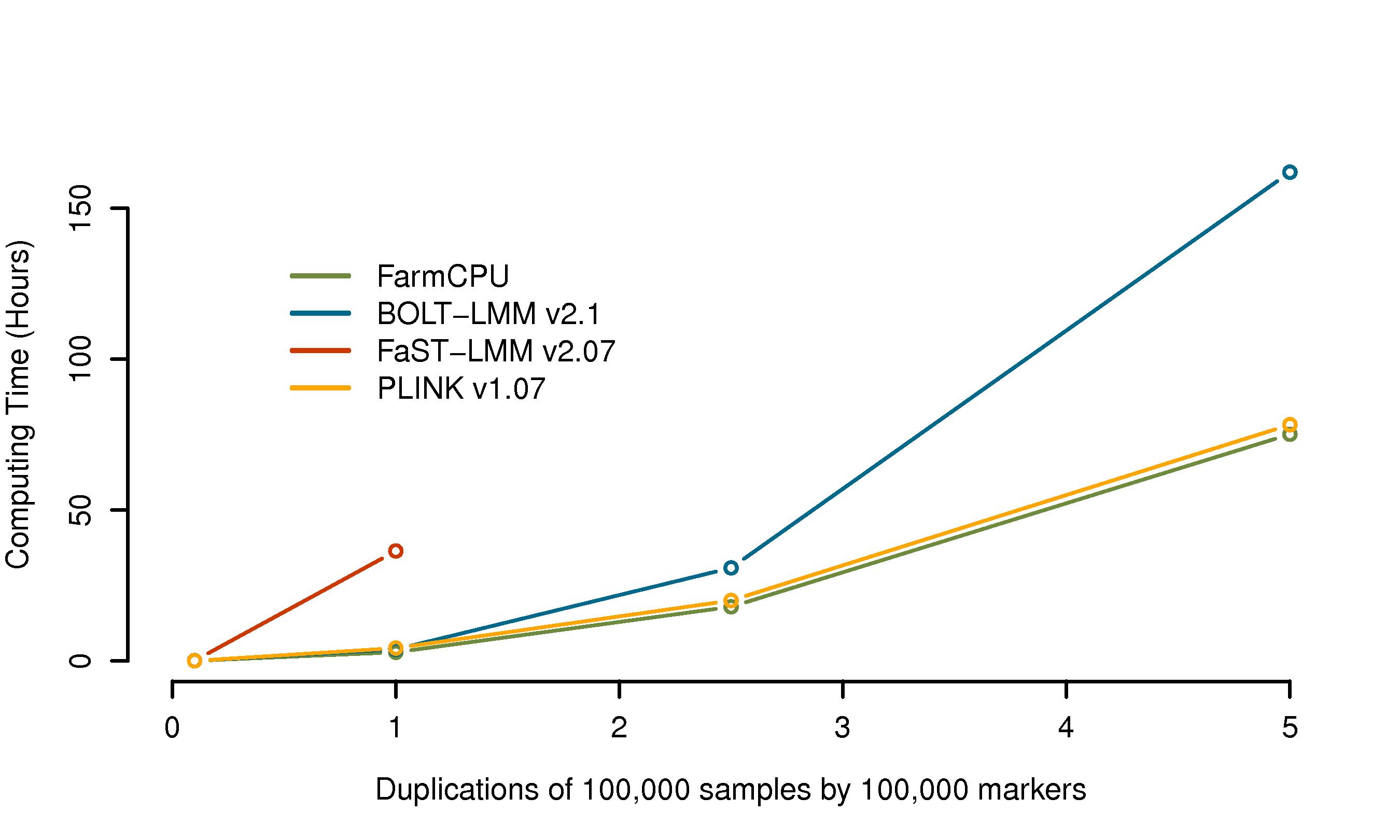
**

**S27 Fig. Computing time on big data analysis.** Four soft wares are used in this study including PLINK (v1.07), FaST-LMM (v2.07), BOLT-LMM (v2.1) and FarmCPU. Computing times in response to data units are displayed, each data unit represents 10,000 samples and each sample includes 10,000 markers. The biggest data for test includes 50 data units (500,000 samples and each sample includes 500,000 markers). The genotype data was generated by PLINK and all markers were unlinked, 3,000 markers were selected as QTNs and the heritability of simulated phenotype is 30%. The analyses were performed on a server running a Linux system (RedHat 7.1, 64 bit) with a 512.0 Gb of Random-Access Memory (RAM), an AMD Opteron(tm) Processor 6376 processor at 2.2GHz, 1TB SSD, and 9.4 TB HDD. One core was used for this test. FarmCPU and PLINK v1.07 completed the analysis on the biggest data in 3 days and BOLT-LMM v2.1 complete it in one week, FaST-LMM v2.07 was stopped by the out of memory issue.
