# Supplementary material for: Iterative Usage of Fixed and Random Effect Models for Powerful and Efficient Genome-Wide Association Studies
Source: PLoS Genet. 2016 Feb 1;12(2):e1005767. doi: 10.1371/journal.pgen.1005767 (PMC4734661; doi:10.1371/journal.pgen.1005767)
Supplement: S28 Fig — (DOCX) [file pgen.1005767.s028.docx]

**
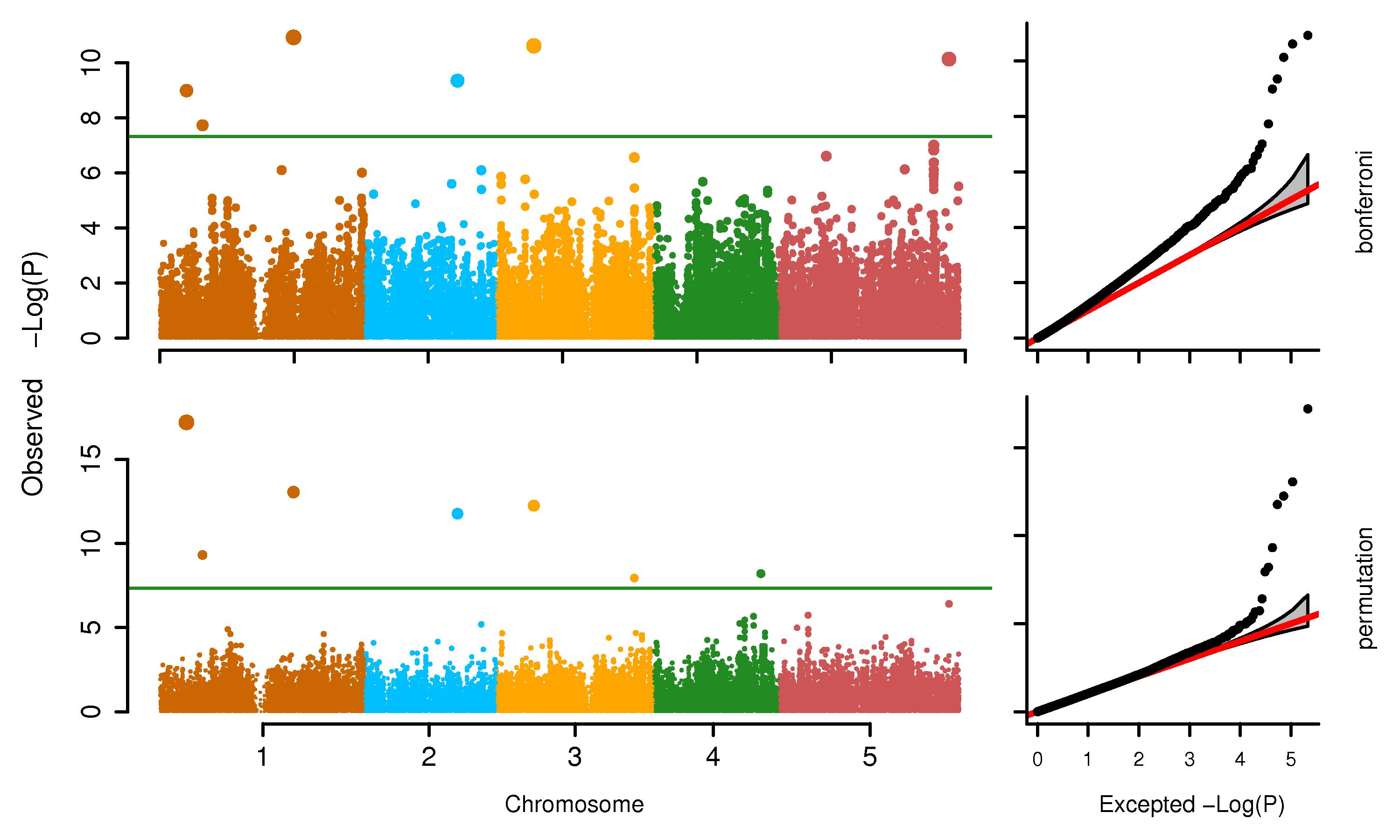
**

**S28 Fig. Performances of different P-value thresholds for selecting pseudo QTNs in FarmCPU.** Two different P value thresholds are tested in this study, Bonferroni multiple test threshold and permutation threshold. Bonferroni multiple test threshold is used in FarmCPU as default. Permutation threshold is calculated from permutation tests, in each test, phenotype is permuted to break the relationship with the genotypes. A vector of minimum P value of each time is recorded and the 95% quantile value of the vector is named as “permutation threshold”. In some cases (e.g. the results of 2 weeks vernalization, grown at JIC (2W) showed in top panels), Bonferroni multiple test threshold is too serious because of the linkage disequilibrium among markers and missed some QTNs with small effects. QQ plots showed permutation threshold makes a better control on the population structure. In addition, simulation results showed different thresholds have the similar performance, the Power versus FDR and Type I error curves are overlapped. So, Bonferroni multiple test threshold works well for most cases and in some cases it doesn’t make FarmCPU control the population very well, spend more time to calculate a permutation threshold to instead of Bonferroni multiple test threshold.
