# Supplementary material for: Iterative Usage of Fixed and Random Effect Models for Powerful and Efficient Genome-Wide Association Studies
Source: PLoS Genet. 2016 Feb 1;12(2):e1005767. doi: 10.1371/journal.pgen.1005767 (PMC4734661; doi:10.1371/journal.pgen.1005767)
Supplement: S1 Table — (DOCX) [file pgen.1005767.s029.docx]

**S1 Table. Top 10 associated SNPs identified by FarmCPU on flowering time in *Arabidopsis thaliana****

| SNP_ID | Chr | Position (base pairs) | P value | Nearby Candidate Genes or QTLs  (base pairs, start: end) |
| --- | --- | --- | --- | --- |
| SNP_1_3190243 | 1 | 3,190,243 | 8.64E-15 | DRB1 (3,137,767:3,140,353) |
| SNP_1_26458686 | 1 | 26,458,686 | 3.26E-13 | MMP (26,423,874:26,425,356) |
| SNP_5_3188328 | 5 | 3,188,328 | 1.99E-12 | FLC (3,173,497:3,179,448) |
| SNP_3_2881111 | 3 | 2,881,111 | 9.74E-10 |  |
| SNP_1_8888799 | 1 | 8,888,799 | 3.67E-08 |  |
| SNP_3_22949500 | 3 | 22,949,500 | 5.33E-07 | PIL2 (22,988,547:22,990,709) |
| SNP_1_27164014 | 1 | 27,164,014 | 6.40E-07 | TFIIIA (27,115,024:27,117,470) |
| SNP_4_15126597 | 4 | 15,126,597 | 8.93E-07 | ATPRMT5 (15,132,011:15,136,639) |
| SNP_1_27161588 | 1 | 27,161,588 | 1.00E-06 | TFIIIA (27,115,024:27,117,470) |
| SNP_1_27164195 | 1 | 27,164,195 | 1.24E-06 | TFIIIA (27,115,024:27,117,470) |

*The flowering time was measured at 16°C. Candidate genes are from the list published by Atwell et.al.
