# Supplementary material for: Iterative Usage of Fixed and Random Effect Models for Powerful and Efficient Genome-Wide Association Studies
Source: PLoS Genet. 2016 Feb 1;12(2):e1005767. doi: 10.1371/journal.pgen.1005767 (PMC4734661; doi:10.1371/journal.pgen.1005767)
Supplement: S2 Table — (DOCX) [file pgen.1005767.s030.docx]

**S2 Table. Top 10 associated SNPs identified by FarmCPU on lung cancer in human*****

| SNP_ID | Chr | Physical position (base pairs) | P value | Nearby Candidate Genes or QTLs (base pairs, start: end) |
| --- | --- | --- | --- | --- |
| rs2736100 | 5 | 1,286,401 | 8.51E-27 | TERT (1,253,167: 1,295,047) |
| rs7086803 | 10 | 112,738,717 | 4.83E-16 | VTI1A: (112,446,998: 112,855,364) |
| rs4867308 | 5 | 31,114,483 | 1.18E-08 | RPL19P11: (31,053,523: 31,054,248); CDH6: (31,193,655: 31,329,146) |
| rs4374829 | 6 | 155,828,142 | 1.05E-07 | LOC101928923: (155,808,725: 156,373,758) |
| rs11612312 | 12 | 51,955,304 | 1.41E-07 | ACVR1B: (51,951,667: 51,997,079) |
| rs11823347 | 11 | 21,192,794 | 1.72E-07 | NELL1: (20,669,551: 21,575,686) |
| rs2968019 | 5 | 59,049,321 | 1.93E-07 | PDE4D: (58,969,038: 60,522,120) |
| rs2106664 | 17 | 61,659,433 | 1.95E-07 |  |
| rs6693140 | 1 | 107,639,325 | 5.79E-07 | VAV3: (107,571,160: 107,964,923) |
| rs1038273 | 3 | 99,067,757 | 6.56E-07 |  |

***** The physical positions of nearby candidate genes are from NCBI (URL: http://www.ncbi.nlm.nih.gov/gene).
