# Supplementary material for: Iterative Usage of Fixed and Random Effect Models for Powerful and Efficient Genome-Wide Association Studies
Source: PLoS Genet. 2016 Feb 1;12(2):e1005767. doi: 10.1371/journal.pgen.1005767 (PMC4734661; doi:10.1371/journal.pgen.1005767)
Supplement: S3 Table — (DOCX) [file pgen.1005767.s031.docx]

**S3 Table. Top 10 associated SNPs identified by FarmCPU on flowering time in maize*****

| SNP_ID | Chr | Physical position (base pairs) | P value | Nearby Candidate Genes or QTLs (base pairs, start: end) |
| --- | --- | --- | --- | --- |
| S10_94253531 | 10 | 94,253,531 | 1.54E-40 | ZmCCT: (94,248,710: 94,251,264) |
| S3_159389461 | 3 | 159,389,461 | 2.54E-15 | Sbp22: (159,420,552: 159,425,244) |
| S10_31079051 | 10 | 31,079,051 | 5.01E-15 | GRMZM2G117028 (31,015,686: 31,021,381) |
| S10_23805684 | 10 | 23,805,684 | 6.84E-15 | GRMZM2G176472 (23,812,687: 23,816,765) |
| S2_202311467 | 2 | 202,311,467 | 2.33E-12 | GRMZM2G179476 (202,964,730: 202,965,761) |
| S8_123473596 | 8 | 123,473,596 | 3.27E-12 | ZCN8: (123,030,387: 123,032,135) |
| S8_131176643 | 8 | 131,176,643 | 1.36E-11 | Vgt1: (132,473,577: 132,963,880) |
| S5_189048393 | 5 | 189,048,393 | 1.77E-11 | GRMZM2G066851 (189,096,630: 189,097,596) |
| S1_188065174 | 1 | 188,065,174 | 8.16E-11 |  |
| S5_5028961 | 5 | 5,028,961 | 1.15E-10 |  |

*Flowering time was measured as days to silk. The candidate genes are from MaizeGDB (URL: <http://maizegdb.org/gene_center/gene>).
