# Supplementary material for: Iterative Usage of Fixed and Random Effect Models for Powerful and Efficient Genome-Wide Association Studies
Source: PLoS Genet. 2016 Feb 1;12(2):e1005767. doi: 10.1371/journal.pgen.1005767 (PMC4734661; doi:10.1371/journal.pgen.1005767)
Supplement: S4 Table — (DOCX) [file pgen.1005767.s032.docx]

**S4 Table. Top 10 associated SNPs identified by FarmCPU on weight growth in mouse*****

| SNP_ID | Chr | Position (centimorgan) | P value | Nearby Candidate Genes or QTLs (centimorgan) |
| --- | --- | --- | --- | --- |
| CEL-10_30960309 | 10 | 16.05990775 | 4.74E-09 | Rspo3 (16.64) |
| rs2020669 | 14 | 37.96958294 | 1.10E-07 | Fndc3a (37.62) |
| rs13481023 | 11 | 28.428282 | 1.45E-07 | Dppa1 (28.02) |
| rs13481364 | 12 | 11.65477273 | 3.46E-07 |  |
| mCV24530618 | 3 | 29.9419253 | 1.07E-06 | Ssr3 (30.15) |
| rs3667738 | 8 | 22.02349987 | 1.19E-06 | Wrn (20.3) |
| rs13483390 | 18 | 38.478198 | 1.79E-06 | Mir122 (38.39) |
| rs13477472 | 3 | 77.20018232 | 6.75E-06 |  |
| rs6155297 | 9 | 0.06146069 | 1.45E-05 |  |
| rs3658204 | 12 | 18.52508228 | 3.20E-05 | Immp2l (18.69) |

*****The candidate genes are from MGI (Mouse Genome Informatics, URL: <http://www.informatics.jax.org/marker>).
