# Supplementary material for: Iterative Usage of Fixed and Random Effect Models for Powerful and Efficient Genome-Wide Association Studies
Source: PLoS Genet. 2016 Feb 1;12(2):e1005767. doi: 10.1371/journal.pgen.1005767 (PMC4734661; doi:10.1371/journal.pgen.1005767)
Supplement: S5 Table — (DOCX) [file pgen.1005767.s033.docx]

**S5 Table. Top 10 associated SNPs identified by FarmCPU on back-fat thickness in pig*****

| SNP_ID | Chr | Physical position (base pairs) | P value | Nearby Candidate Genes or QTLs (million base pairs, start: end) |
| --- | --- | --- | --- | --- |
| ALGA0096104 | 17 | 50,461,052 | 2.03E-09 | QTL #1191 (46.3:67.9) |
| ALGA0025287 | 4 | 56,857,008 | 2.26E-08 | QTL #7271 (38.9:61.9) |
| ASGA0075004 | 17 | 2,287,265 | 5.14E-07 | QTL #12538 (0.4:19.2) |
| DRGA0010301 | 10 | 9,397,877 | 2.88E-06 |  |
| ASGA0057437 | 13 | 30,331,879 | 3.59E-06 | QTL #16840 (16.6:67.3) |
| ASGA0033098 | 7 | 43,236,720 | 5.23E-06 | QTL #16837 (36.5:63.4) |
| ALGA0006623 | 1 | 139,472,483 | 6.68E-06 | QTL #3794 (105.1:274.1) |
| ALGA0004042 | 1 | 57,168,010 | 8.93E-06 |  |
| ALGA0006893 | 1 | 146,201,596 | 1.43E-05 | QTL #3794 (105.1:274.1) |
| ALGA0026196 | 4 | 76,603,552 | 1.49E-05 | QTL #411 (53.0:95.9) |

***** The candidate genes are from PigQTLdb (Pig Quantitative Trait Locus Database, URL: <http://www.animalgenome.org/cgi-bin/QTLdb/SS/index>).
