# Supplementary material for: Iterative Usage of Fixed and Random Effect Models for Powerful and Efficient Genome-Wide Association Studies
Source: PLoS Genet. 2016 Feb 1;12(2):e1005767. doi: 10.1371/journal.pgen.1005767 (PMC4734661; doi:10.1371/journal.pgen.1005767)
Supplement: S6 Table — (DOCX) [file pgen.1005767.s034.docx]

**S6 Table. Significant SNPs detected by FarmCPU are overlapped with previously published results for lung cancer*****

| SNP_ID | Author | Time | Journal | Title |
| --- | --- | --- | --- | --- |
| rs2736100 | Lan Q et.al | November 11, 2012 | *Nat Genet* | Genome-wide association analysis identifies new lung cancer susceptibility loci in never-smoking women in Asia |
|  | Hu Z et.al | July 03, 2011 | *Nat Genet* | A genome-wide association study identifies two new lung cancer susceptibility loci at 13q12.12 and 22q12.2 in Han Chinese |
|  | McKay JD et.al | November 02, 2008 | *Nat Genet* | Lung cancer susceptibility locus at 5p15.33 |
| rs7086803 | Lan Q et.al | November 11, 2012 | *Nat Genet* | Genome-wide association analysis identifies new lung cancer susceptibility loci in never-smoking women in Asia |

***** 29 studies were included in human GWAS Catalog (http://www.genome.gov/gwastudies/). Among those studies, top significant SNPs detected in MLM and FarmCPU are overlapped with several previous studies. On the contrast, the most significant SNP detected in t-test and GLM has never reported.
