# Supplementary material for: Iterative Usage of Fixed and Random Effect Models for Powerful and Efficient Genome-Wide Association Studies
Source: PLoS Genet. 2016 Feb 1;12(2):e1005767. doi: 10.1371/journal.pgen.1005767 (PMC4734661; doi:10.1371/journal.pgen.1005767)
Supplement: S7 Table — (DOCX) [file pgen.1005767.s035.docx]

**S7 Table. Observed number of false positives for FarmCPU (FC) versus t-test (T) at different P-value thresholds in simulation*****

| P-value Threshold | 10,000 bps | | 50,000 bps | | 100,000 bps | | 500,000 bps | | 1,000,000 bps | |
| --- | --- | --- | --- | --- | --- | --- | --- | --- | --- | --- |
|  | T | FC | T | FC | T | FC | T | FC | T | FC |
| 1e-4 | 22,037 | 5,533 | 12,297 | 4,352 | 10,300 | 4,046 | 7,784 | 3,584 | 6,739 | 3,303 |
| 1e-6 | 9,456 | 540 | 3,229 | 193 | 2,043 | 119 | 911 | 57 | 678 | 51 |
| 0.05bonf^a^ | 8,293 | 435 | 2,710 | 130 | 1,655 | 72 | 660 | 21 | 480 | 18 |
| 0.01bonf^b^ | 7,712 | 415 | 2,487 | 122 | 1,493 | 66 | 578 | 17 | 421 | 14 |

***** The genotype data is from East Asian lung cancer dataset. Phenotypes with 50% heritability and controlled by 100 QTNs were simulated across the whole genome. A false positive was recorded if there is no QTN on either side of a significant SNP within a specific resolution (i.e., 10,000, 50,000, 100,000, 500,000, 1,000,000 base pairs (bps)). Numbers of false positives of FarmCPU and t-test under different P-value thresholds were recorded. The experiments were repeated 100 times and total number of false positives in each threshold was displayed in the table. ^a^ A threshold of 5% after Bonferroni multiple test correction. ^b^ A threshold of 1% after Bonferroni multiple test correction.
