# Supplementary material for: Iterative Usage of Fixed and Random Effect Models for Powerful and Efficient Genome-Wide Association Studies
Source: PLoS Genet. 2016 Feb 1;12(2):e1005767. doi: 10.1371/journal.pgen.1005767 (PMC4734661; doi:10.1371/journal.pgen.1005767)
Supplement: S8 Table — (DOCX) [file pgen.1005767.s036.docx]

**S8 Table. Observed versus expected number of false positives for FarmCPU at different P-value thresholds in the null distribution *****

| P-value Threshold | 95% Confidence Limits (Lower) | FarmCPU | Expected | 95% Confidence Limits (Upper) |
| --- | --- | --- | --- | --- |
| 0.05 | 880,395 | 882,253 | 881,900 | 883,406 |
| 1e-4 | 1,695 | 1,787 | 1,763 | 1,833 |
| 1e-6 | 11 | 23 | 17 | 25 |
| 0.05bonf^a^ | 0 | 2 | 2 | 4 |
| 0.025bonf^b^ | 0 | 0 | 0 | 3 |
| 0.01bonf^c^ | 0 | 0 | 0 | 2 |

***** The dataset is from WTCCC1 controls human population consists of 1,500 individuals genotyped with 495,473 SNPs.100 QTNs were simulated in the first 10 Chromosomes and no QTNs in other Chromosomes (Chromosomes 11-22, X). Numbers of significant markers of FarmCPU under different p value thresholds in Chromosomes 11-22, X were recorded. The expected numbers of false positives are generated from a uniform distribution. The experiments were repeated 100 times and total number of false positives in each threshold was displayed in the table. ^a^ A threshold of 5% after Bonferroni multiple test correction. ^b^ A threshold of 2.5% after Bonferroni multiple test correction. ^c^ A threshold of 1% after Bonferroni multiple test correction.
