# Supplementary material for: Iterative Usage of Fixed and Random Effect Models for Powerful and Efficient Genome-Wide Association Studies
Source: PLoS Genet. 2016 Feb 1;12(2):e1005767. doi: 10.1371/journal.pgen.1005767 (PMC4734661; doi:10.1371/journal.pgen.1005767)
Supplement: S10 Table — (DOCX) [file pgen.1005767.s038.docx]

**S10 Table. Computing time complexity among statistical methods for Genome-Wide Association Studies*****

| **Methods** | **Kinship** | **Optimization** | **Association Test** |
| --- | --- | --- | --- |
| GLM | NA | NA |  |
| MLM |  | NA^a^ |  |
| EMMAX/P3D |  |  |  |
| GRAMMAR-Gamma |  |  |  |
| FaST-LMM^b^ | NA^c^ | $O(M^{2}n)$ | $O(mMn)$ |
| FaST-LMM-Select^b^ | NA^c^ | $O(M^{2}n)$ | $O(mMn)$ |
| SUPER^b^ | NA^c^ | $O(M^{2}n)$ | $O(mMn)$ |
| MLMM |  |  |  |
| FarmCPU | NA^c^ | $O(M^{2}n)$ |  |

***** The computing process is classified into three categories-calculation of kinship, optimization of variance components or their ratio, and association test on markers. Parameters impacting complexity include the following: sample size (n), total number of marker (m), and number of markers (M) used to build the kinship matrix. ^a^ The optimization is performed for each testing marker. ^b^ These methods choose M that is smaller than n. ^c^ These methods do not construct kinship matrix.
