# Supplementary material for: Iterative Usage of Fixed and Random Effect Models for Powerful and Efficient Genome-Wide Association Studies
Source: PLoS Genet. 2016 Feb 1;12(2):e1005767. doi: 10.1371/journal.pgen.1005767 (PMC4734661; doi:10.1371/journal.pgen.1005767)
Supplement: S11 Table — (DOCX) [file pgen.1005767.s039.docx]

**S11 Table. Software versions and codes for building kinship matrices and association tests.**

| Software | Version | Building kinship | Association tests |
| --- | --- | --- | --- |
| PLINK | PLINK v1.07 |  | ./plink --tfile tfile_data --linear --out plinkresult |
| EMMAX | EMMAX beta as of March 7, 2010 | ./emmax-kin -v -h -d 10 tfile_data | ./emmax -v -d 10 -t tfile_data -p phenotype.txt -k tfile_data.hBN.kinf -o results.txt |
| GenABEL | GenABEL v1.8-0 | gkin<-ibs(GenABELdata,weight="freq")  diag(gkin)<-hom(GenABELdata)$Var | h2ht<-polygenic(phenotype,kin=gkin,data=GenABELdata)  grammar_results<-grammar(h2ht,data=GenABELdata,method="gamma") |
| MLMM | MLMM Jun12 2012 10:57 AM | MLMM recommends using EMMAX calculating kinship matrix | MLMMresults  <- mlmm(phenotype,genotype,emmax_kinship,10,5) |
| FarmCPU | FarmCPU v1.0 |  | FarmCPU_results <- FarmCPU(Y=phenotype,GD=genotype,GM=SNP_map_information,maxLoop=5) |
