# Supplementary material for: Iterative Usage of Fixed and Random Effect Models for Powerful and Efficient Genome-Wide Association Studies
Source: PLoS Genet. 2016 Feb 1;12(2):e1005767. doi: 10.1371/journal.pgen.1005767 (PMC4734661; doi:10.1371/journal.pgen.1005767)
Supplement: S1 File — (DOCX) [file pgen.1005767.s040.docx]

**S29-S134 Figs. Association studies of 106 traits in *Arabidopsis thaliana*.**

***All acronyms of phenotypes in S29-S134 Figs are the same with previous study^21^. For flowering time phenotypes, top 10 SNPs with most significant P values were marked with candidate genes.***

That some results look a little different from previous study is because the simple model in Atwell’s paper is using Wilcox test and we use t-test; the complex model in Atwell’s paper is using EMMA and we use a different kinship algorithm (VanRaden et al. 2008 J Dairy Sci.) and added first 3 columns of principal components as fixed effects.

**
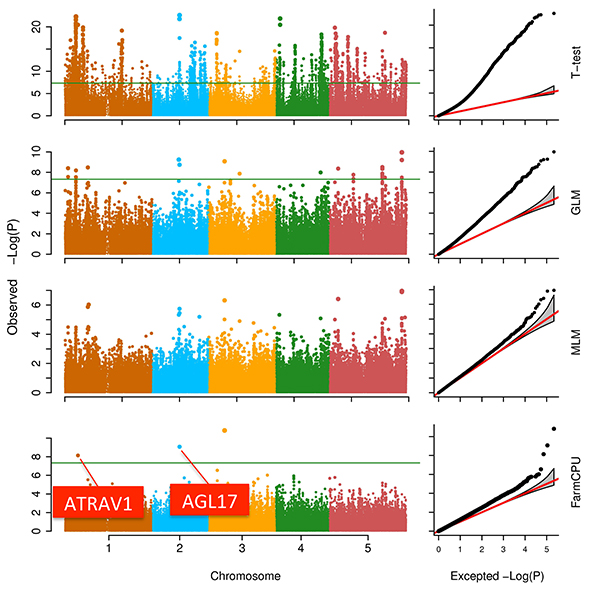
**

**S29 Fig. GWAS results of Days to Flowering under Long Days (LD) using four models (naïve model (t-test), GLM, MLM and FarmCPU).**

**
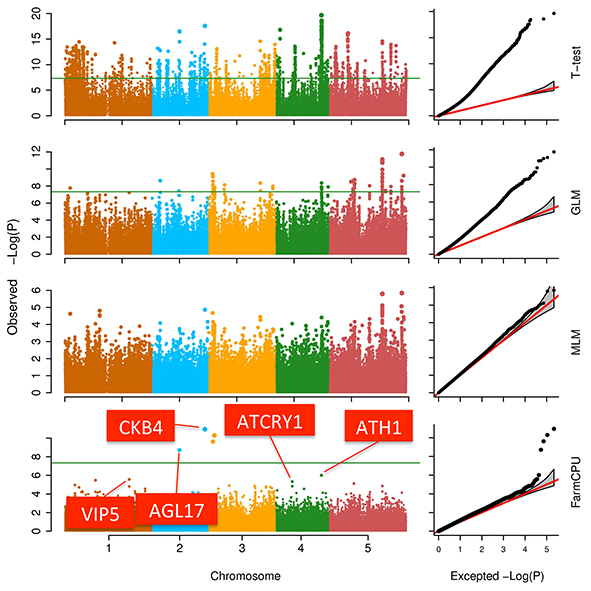
**

**S30 Fig. GWAS results of Days to Flowering under Long Days with Vernalization (LDV) using four models (naïve model (t-test), GLM, MLM and FarmCPU).**

**
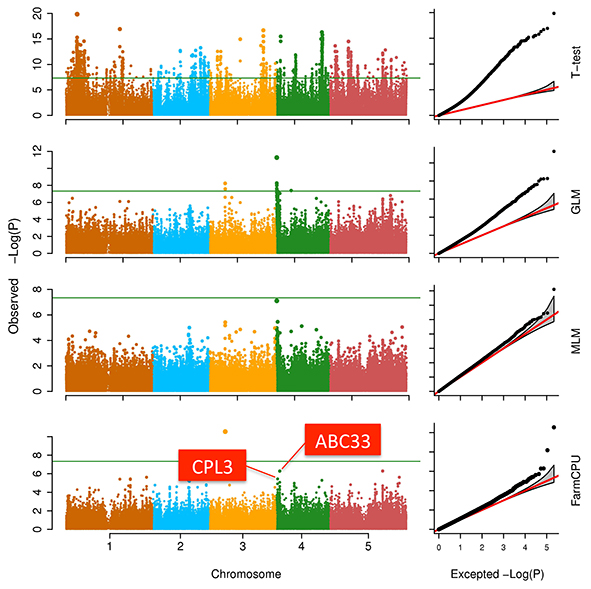
**

**S31 Fig. GWAS results of Days to Flowering under Short Days (SD) using four models (naïve model (t-test), GLM, MLM and FarmCPU).**

**
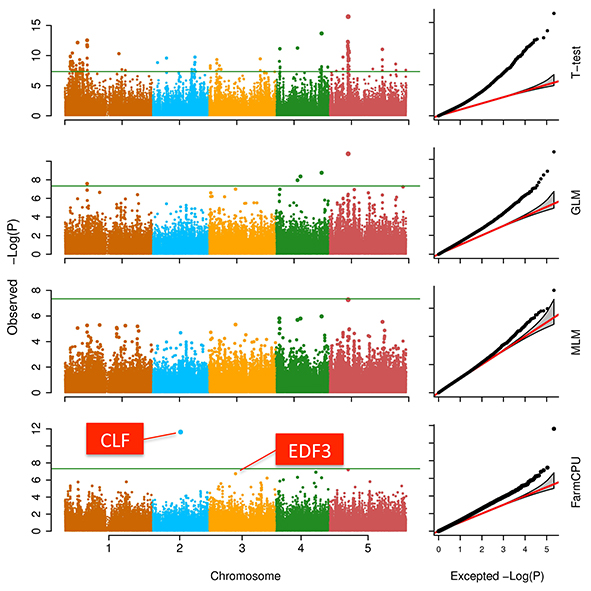
**

**S32 Fig. GWAS results of Days to Flowering under Short Days with Vernalization (SDV) using four models (naïve model (t-test), GLM, MLM and FarmCPU).**

**
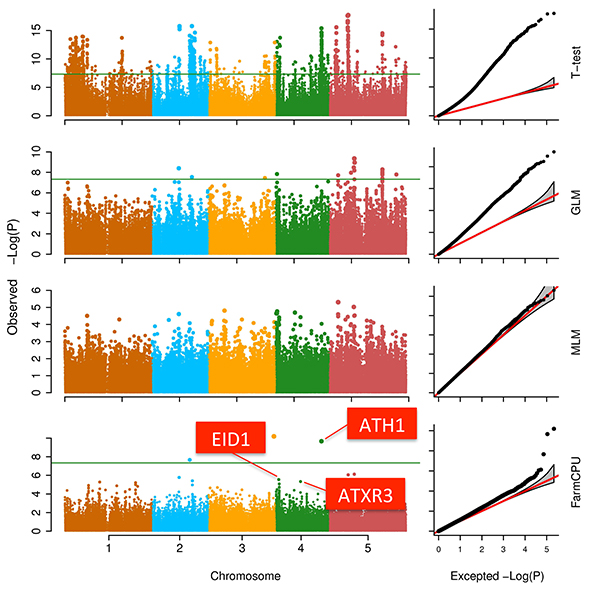
**

**S33 Fig. GWAS results of Days to Flowering at 10◦C (FT10) using models (naïve model (t-test), GLM, MLM and FarmCPU).**

**
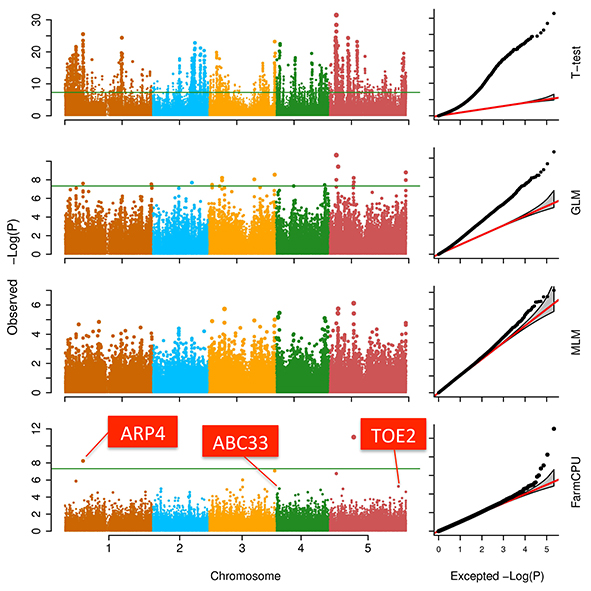
**

**S34 Fig. GWAS results of Days to Flowering at 22◦C (FT22) using four models (naïve model (t-test), GLM, MLM and FarmCPU).**

**
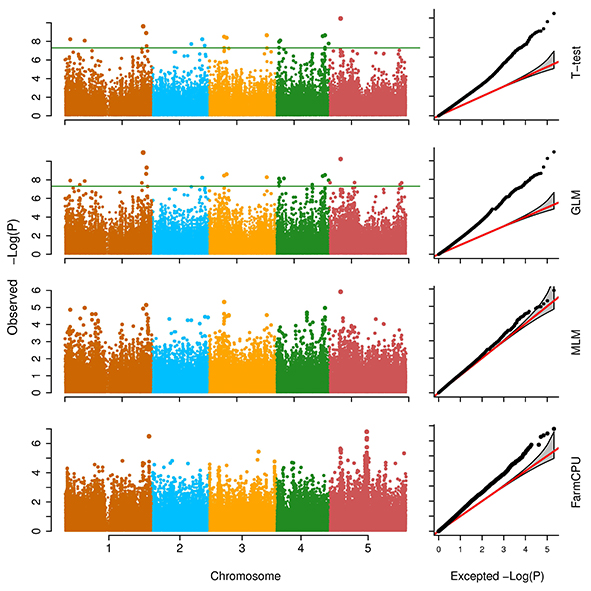
**

**S35 Fig. GWAS results of Seed Dormancy (SD) using four models (naïve model (t-test), GLM, MLM and FarmCPU).**

**
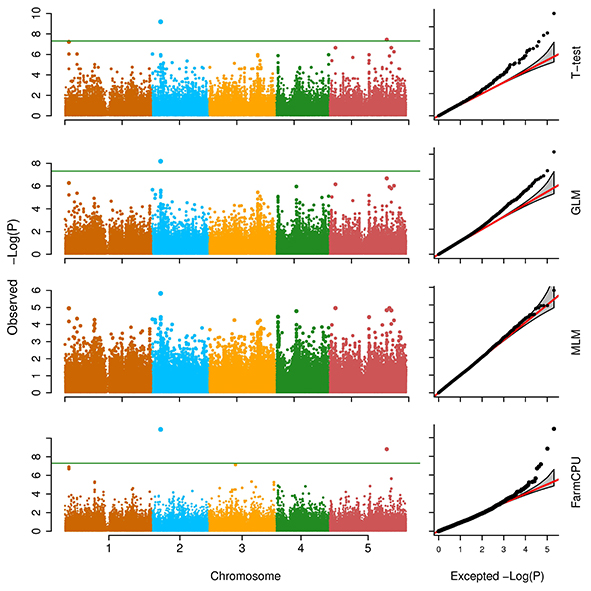
**

**S36 Fig. GWAS results of Emco5 using four models (naïve model (t-test), GLM, MLM and FarmCPU).**

**
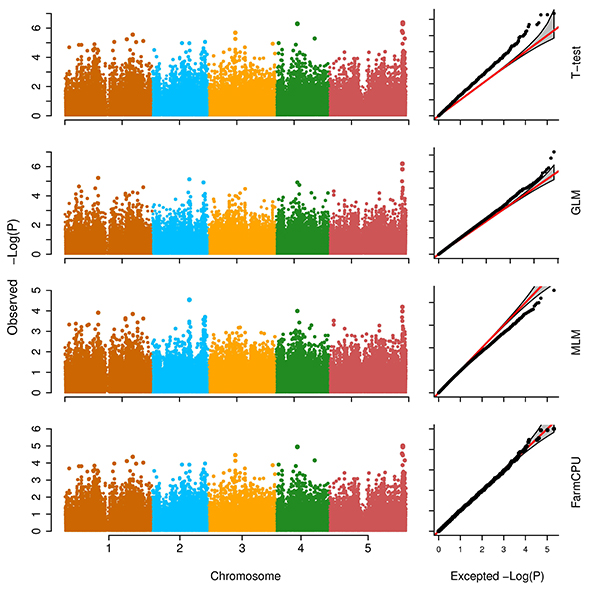
**

**S37 Fig. GWAS results of Emwa1 using four models (naïve model (t-test), GLM, MLM and FarmCPU).**

**
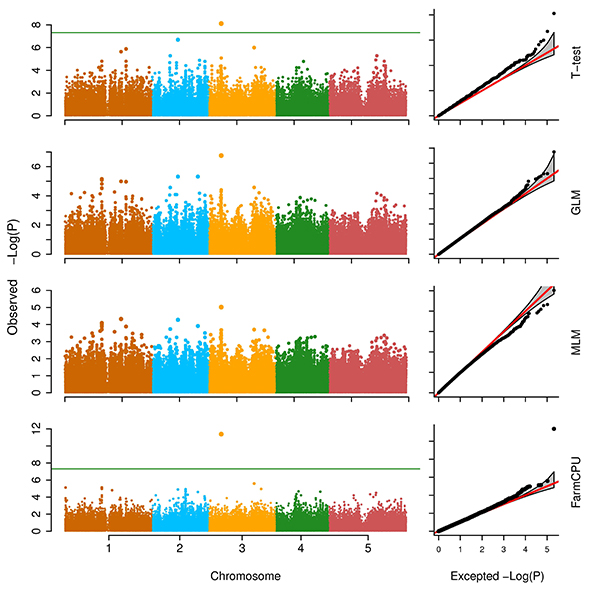
**

**S38 Fig. GWAS results of Emoy2 using four models (naïve model (t-test), GLM, MLM and FarmCPU).**

**
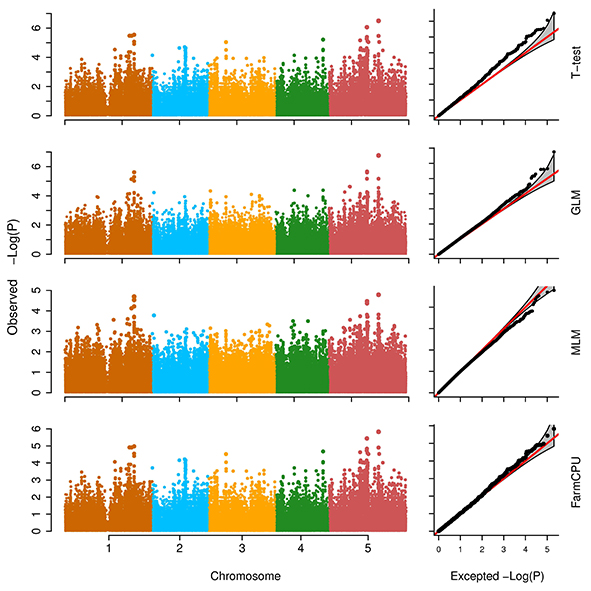
**

**S39 Fig. GWAS results of Hiks1 using four models (naïve model (t-test), GLM, MLM and FarmCPU).**

**
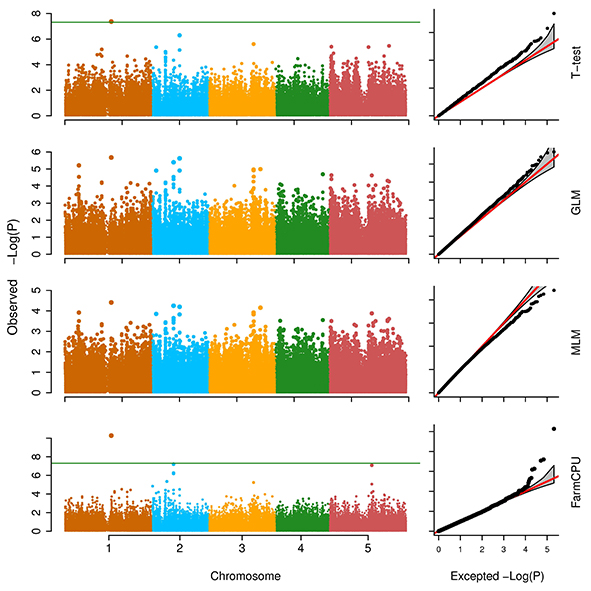
**

**S40 Fig. GWAS results of Noco2 using four models (naïve model (t-test), GLM, MLM and FarmCPU).**

**
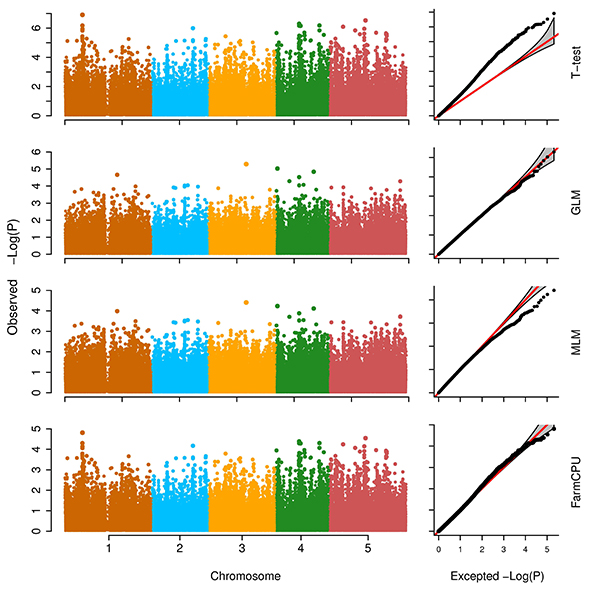
**

**S41 Fig. GWAS results of Lithium concentration (Li) four models (naïve model (t-test), GLM, MLM and FarmCPU).**

**
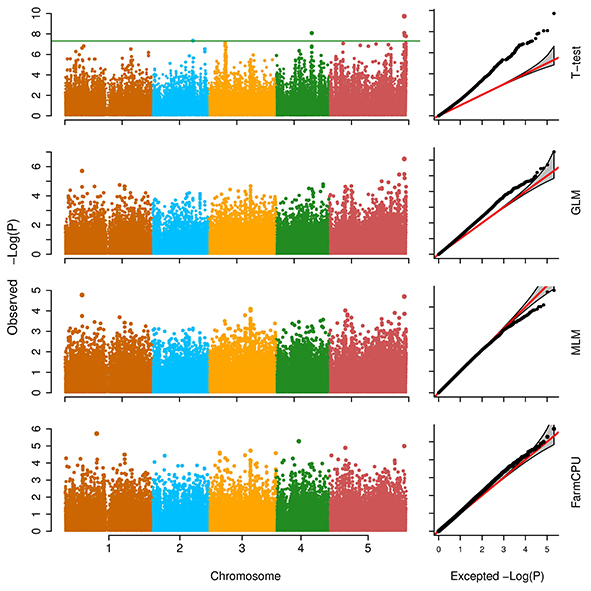
**

**S42 Fig. GWAS results of Boron concentration (B) using four models (naïve model (t-test), GLM, MLM and FarmCPU).**

**
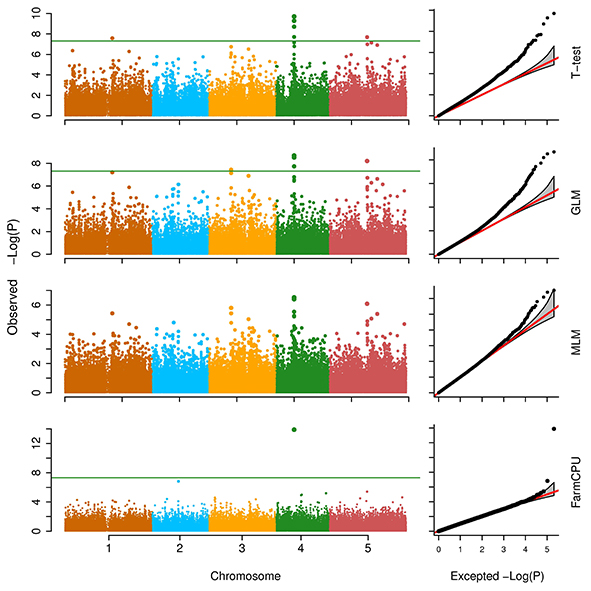
**

**S43 Fig. GWAS results of Sodium concentration (Na) using four models (naïve model (t-test), GLM, MLM and FarmCPU).**

**
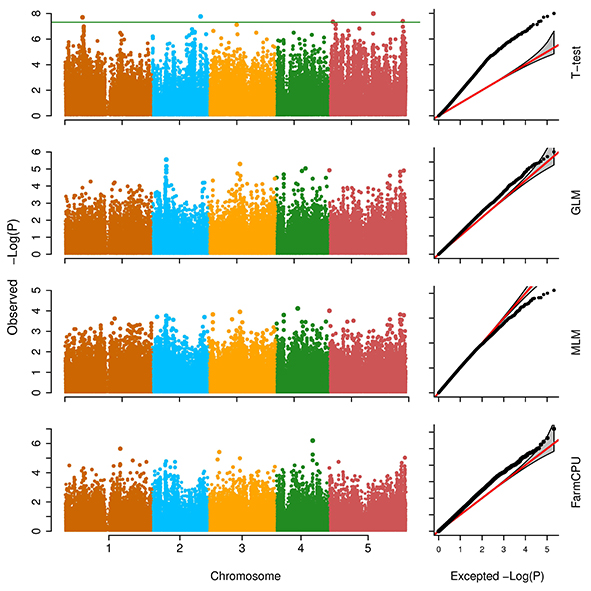
**

**S44 Fig. GWAS results of Magnesium concentration (Mg) using four models (naïve model (t-test), GLM, MLM and FarmCPU).**

**
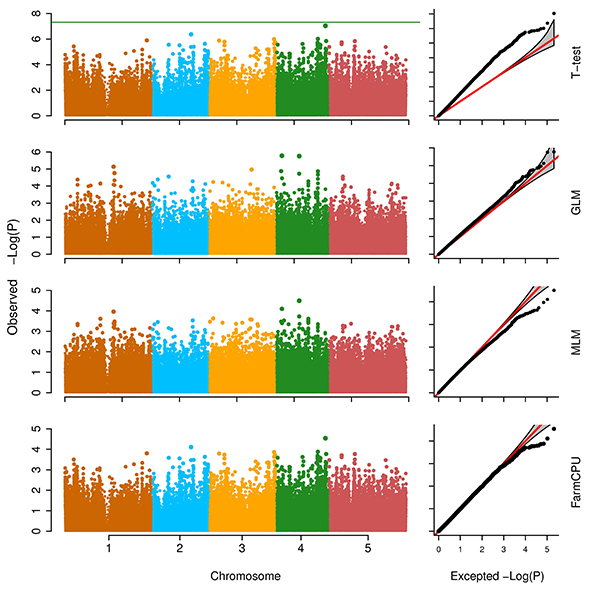
**

**S45 Fig. GWAS results of Phosphorus concentration (P) using four models (naïve model (t-test), GLM, MLM and FarmCPU).**

**
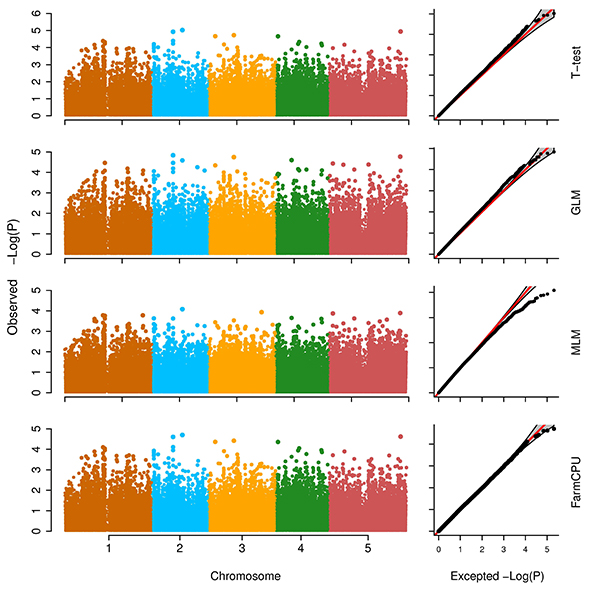
**

**S46 Fig. GWAS results of Sulfur concentration (S) using four models (naïve model (t-test), GLM, MLM and FarmCPU).**

**
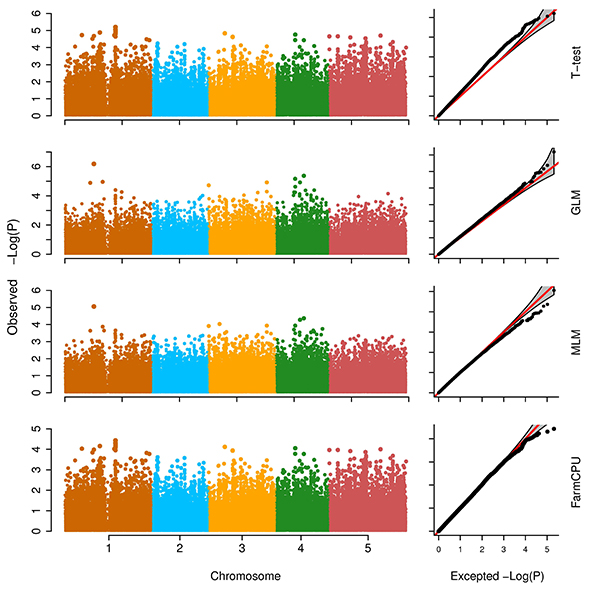
**

**S47 Fig. GWAS results of Potassium concentration (K) using four models (naïve model (t-test), GLM, MLM and FarmCPU).**

**
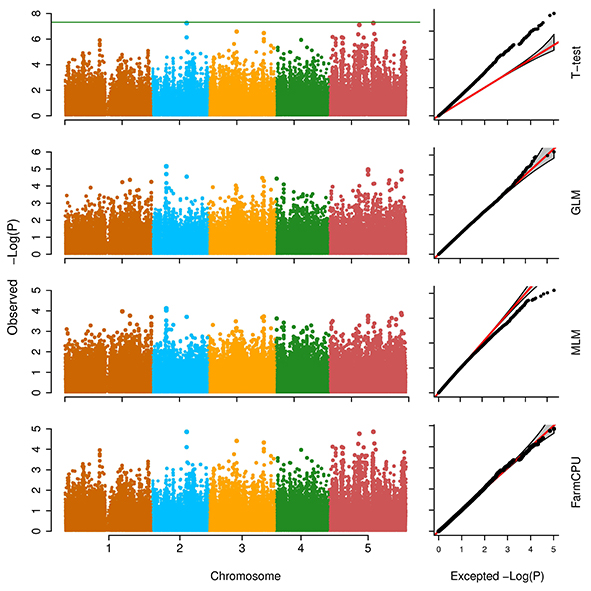
**

**S48 Fig. GWAS results of Calcium concentration (Ca) using four models (naïve model (t-test), GLM, MLM and FarmCPU).**

**
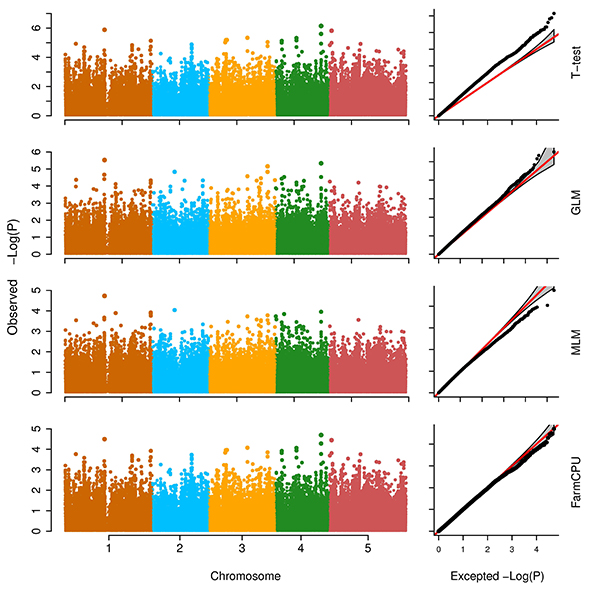
**

**S49 Fig. GWAS results of Manganese concentration (Mn) using four models (naïve model (t-test), GLM, MLM and FarmCPU).**

**
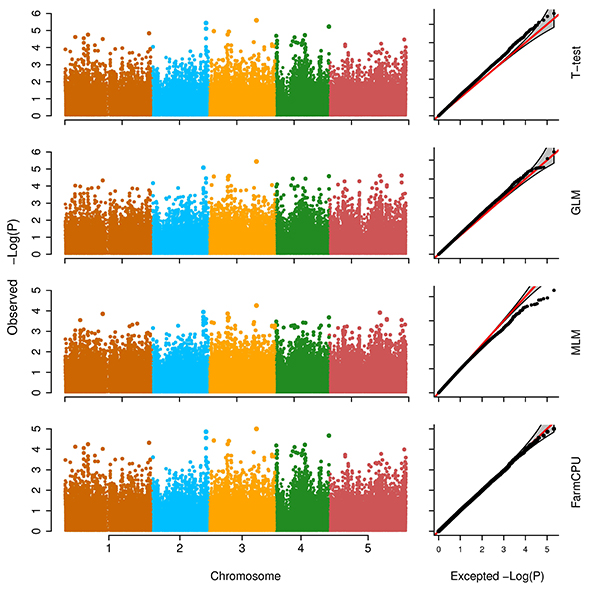
**

**S50 Fig. GWAS results of Iron concentration (Fe) using four models (naïve model (t-test), GLM, MLM and FarmCPU).**

**
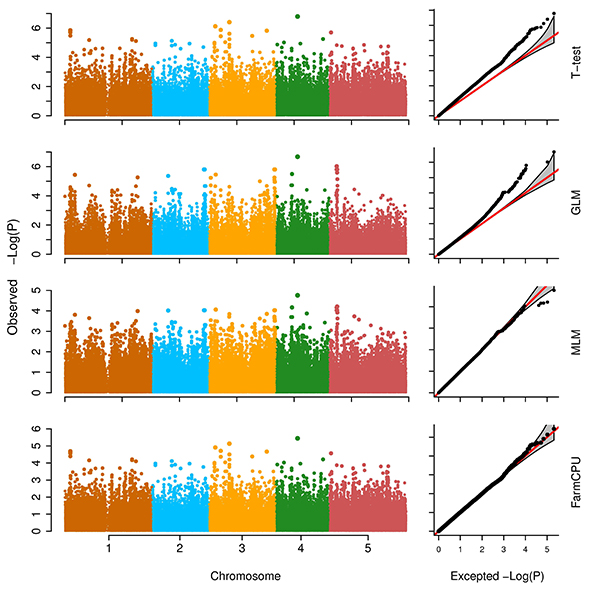
**

**S51 Fig. GWAS results of Cobolt concentration (Co) using four models (naïve model (t-test), GLM, MLM and FarmCPU).**

**
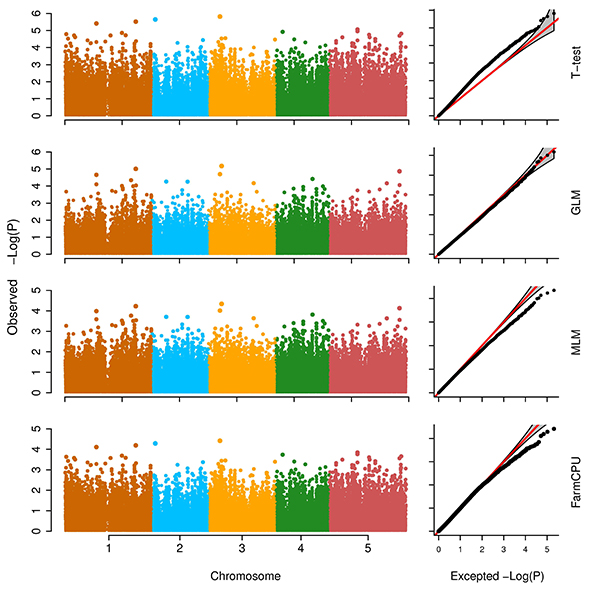
**

**S52 Fig. GWAS results of Nickel concentration (Ni) using four models (naïve model (t-test), GLM, MLM and FarmCPU).**

**
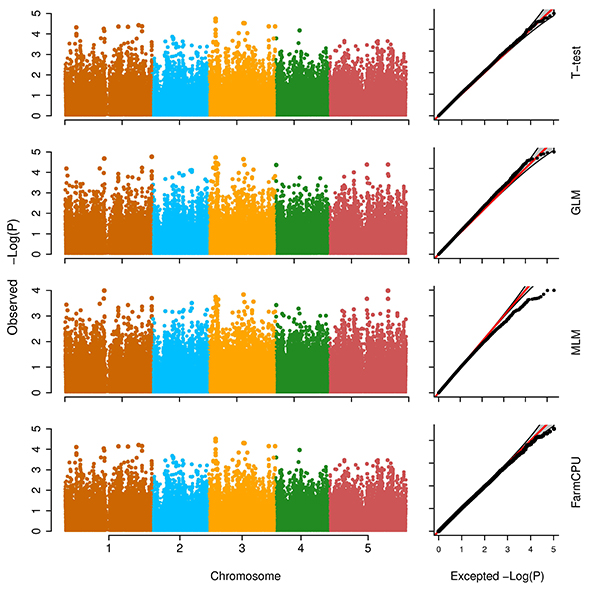
**

**S53 Fig. GWAS results of Copper concentration (Cu) using four models (naïve model (t-test), GLM, MLM and FarmCPU).**

**
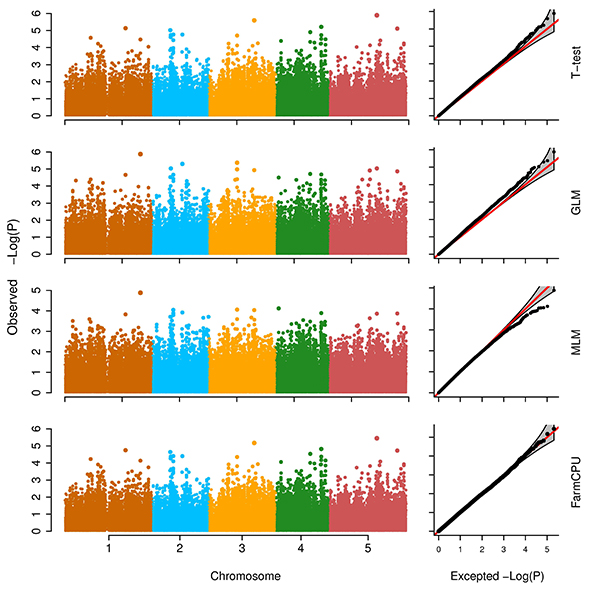
**

**S54 Fig. GWAS results of Zinc concentration (Zn) using four models (naïve model (t-test), GLM, MLM and FarmCPU).**

**
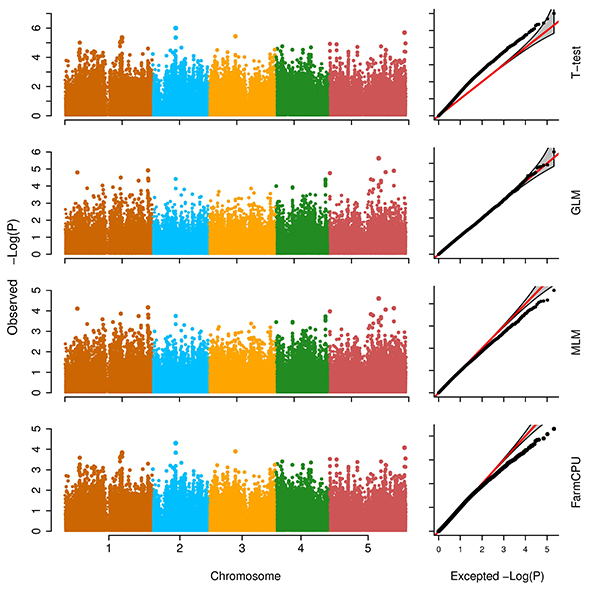
**

**S55 Fig. GWAS results of Arsenic concentration (As) using four models (naïve model (t-test), GLM, MLM and FarmCPU).**

**
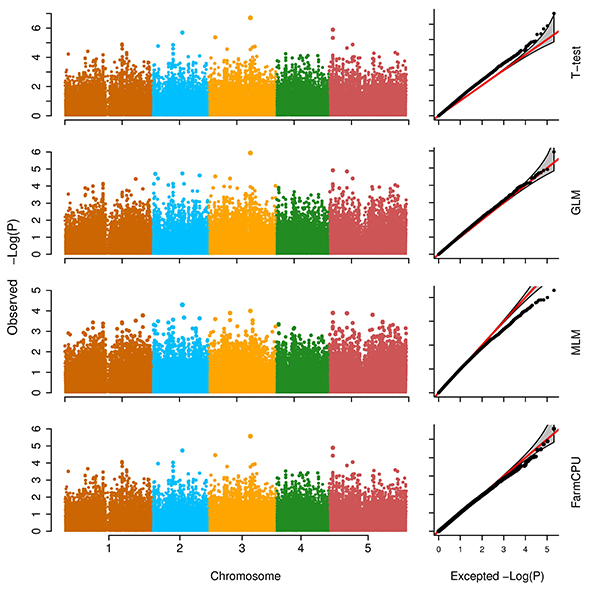
**

**S56 Fig. GWAS results of Selenium concentration (Se) using four models (naïve model (t-test), GLM, MLM and FarmCPU).**

**
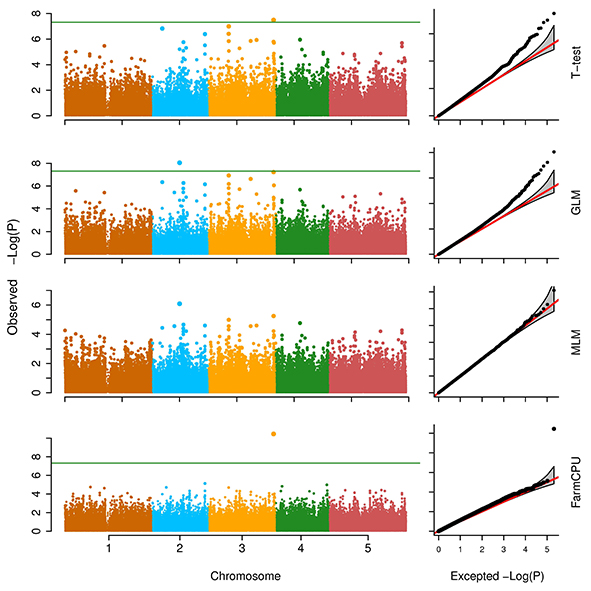
**

**S57 Fig. GWAS results of Molybdenum concentration (Mo) using four models (naïve model (t-test), GLM, MLM and FarmCPU).**

**
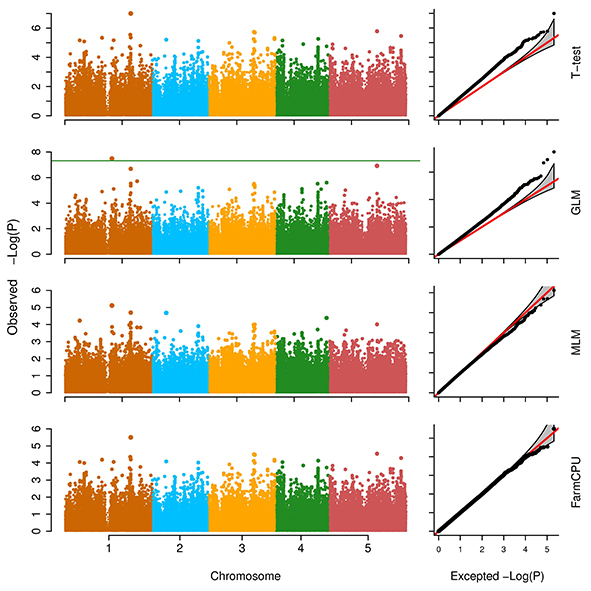
**

**S58 Fig. GWAS results of Cadmium concentration (Cd) using four models (naïve model (t-test), GLM, MLM and FarmCPU).**

**
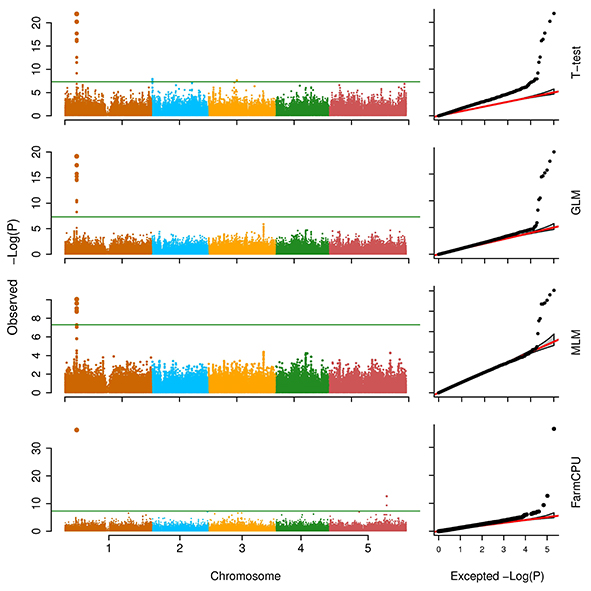
**

**S59 Fig. GWAS results of AvrPphB using four models (naïve model (t-test), GLM, MLM and FarmCPU).**

**
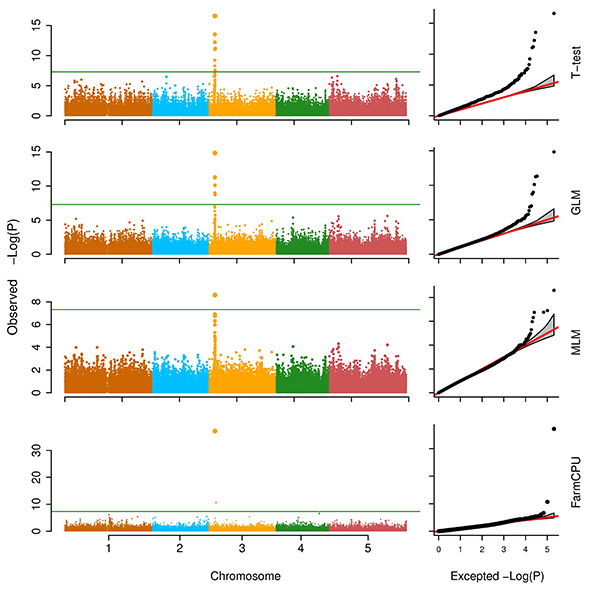
**

**S60 Fig. GWAS results of AvrRpm1 using four models (naïve model (t-test), GLM, MLM and FarmCPU).**

**
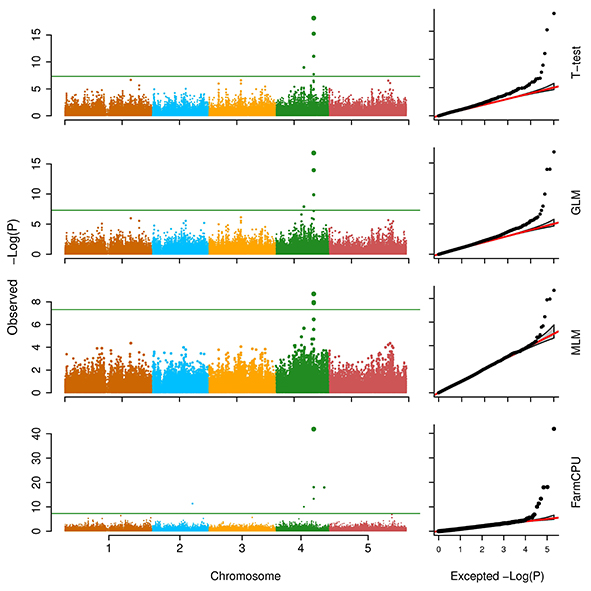
**

**S61 Fig. GWAS results of AvrRpt2 using four models (naïve model (t-test), GLM, MLM and FarmCPU).**

**
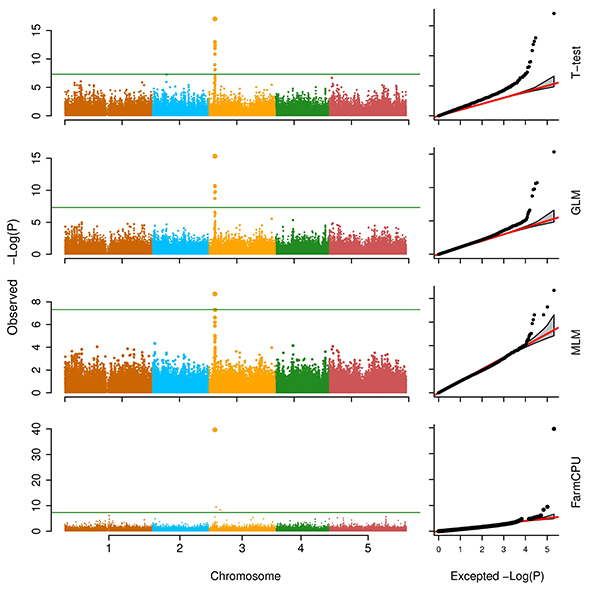
**

**S62 Fig. GWAS results of AvrB using four models (naïve model (t-test), GLM, MLM and FarmCPU).**

**
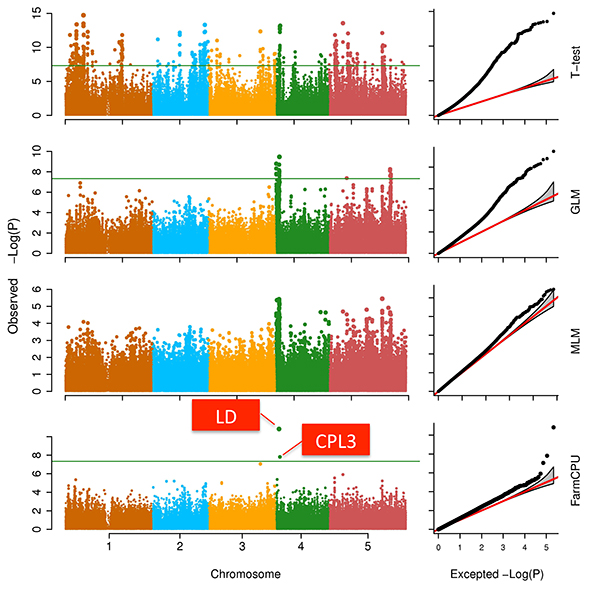
**

**S63 Fig. GWAS results of No vernalization, grown at JIC (0W) using four models (naïve model (t-test), GLM, MLM and FarmCPU).**

**
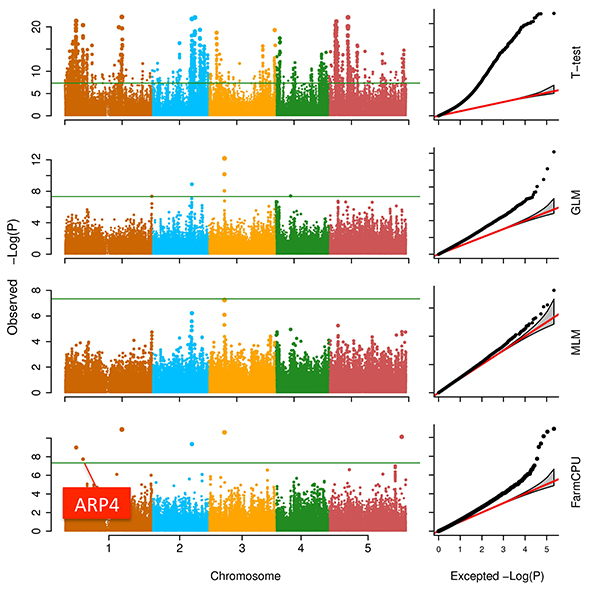
**

**S64 Fig. GWAS results of 2 weeks vernalization, grown at JIC (2W) using four models (naïve model (t-test), GLM, MLM and FarmCPU).**

**
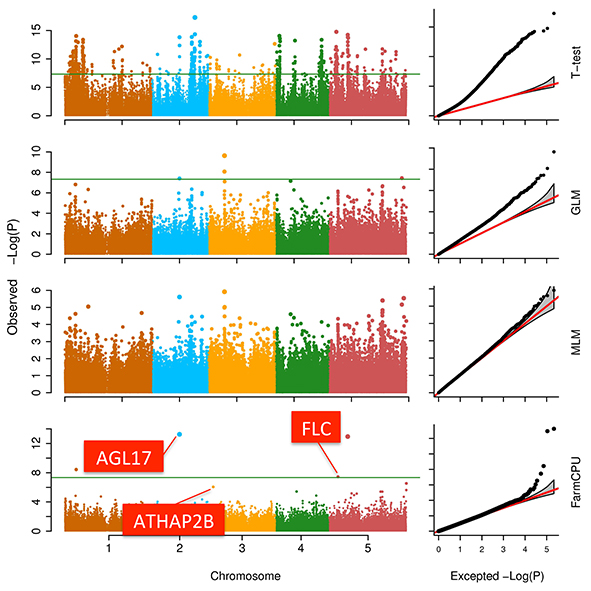
**

**S65 Fig. GWAS results of 4 weeks vernalization, grown at JIC (4W) using four models (naïve model (t-test), GLM, MLM and FarmCPU).**

**
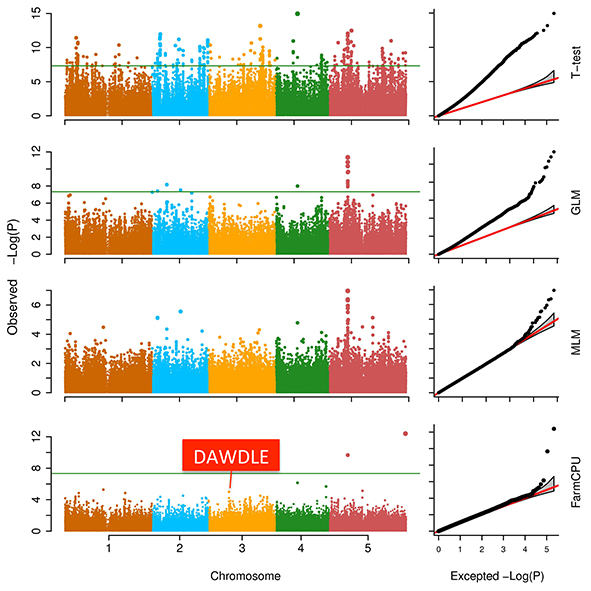
**

**S66 Fig. GWAS results of 8 weeks vernalization, grown at JIC (8W) using four models (naïve model (t-test), GLM, MLM and FarmCPU).**

**
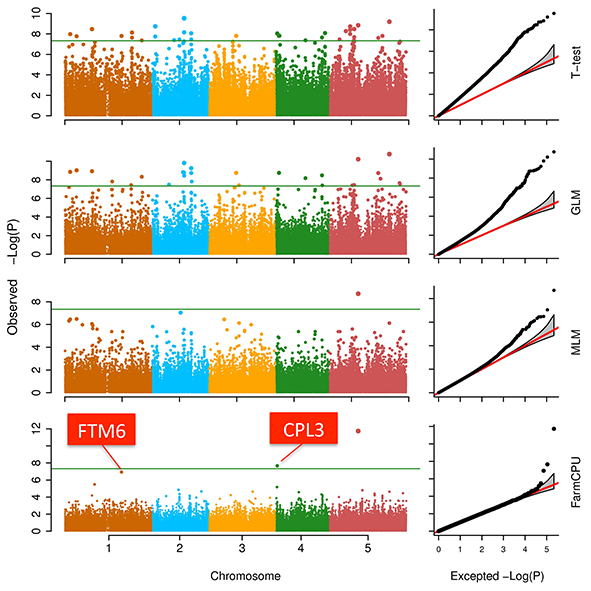
**

**S67 Fig. GWAS results of FLC gene expression using four models (naïve model (t-test), GLM, MLM and FarmCPU).**

**
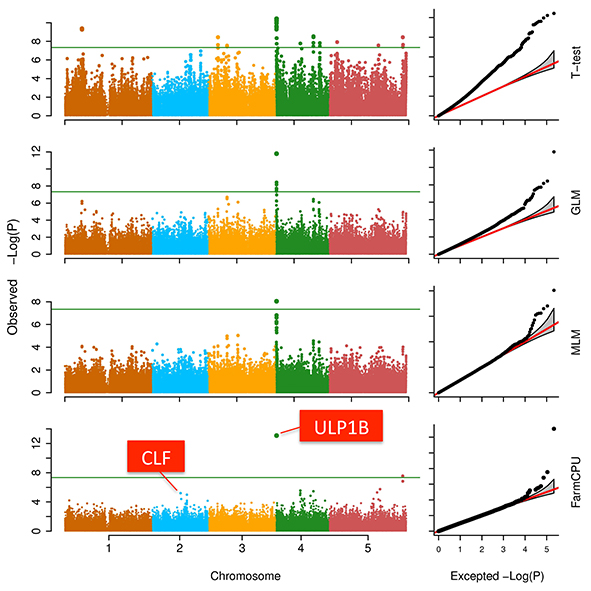
**

**S68 Fig. GWAS results of FRI gene expression (FRI) using four models (naïve model (t-test), GLM, MLM and FarmCPU).**

**
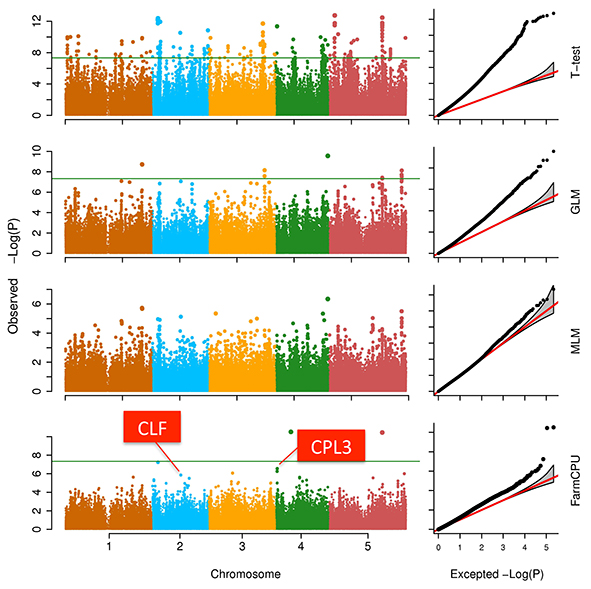
**

**S69 Fig. GWAS results of Days to Flowering, 8wks vernalization, greenhouse (8W GH FT) using four models (naïve model (t-test), GLM, MLM and FarmCPU).**

**
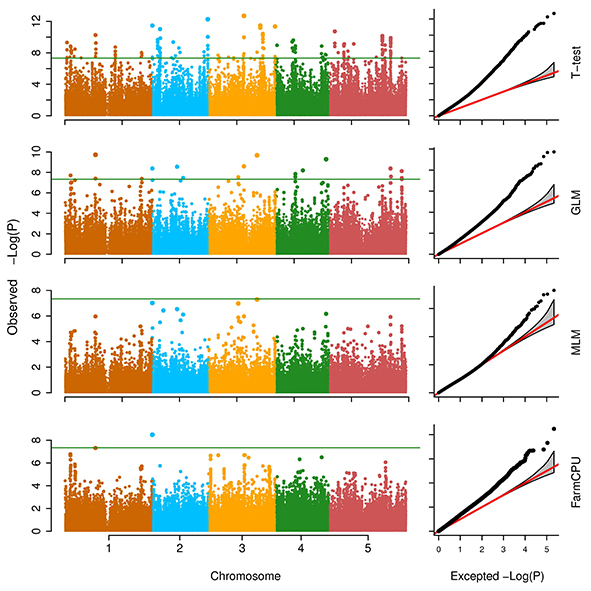
**

**S70 Fig. GWAS results of Leaf Number at Flowering with 8 wks vernalization, greenhouse (8W GH LN) using four models (naïve model (t-test), GLM, MLM and FarmCPU).**

**
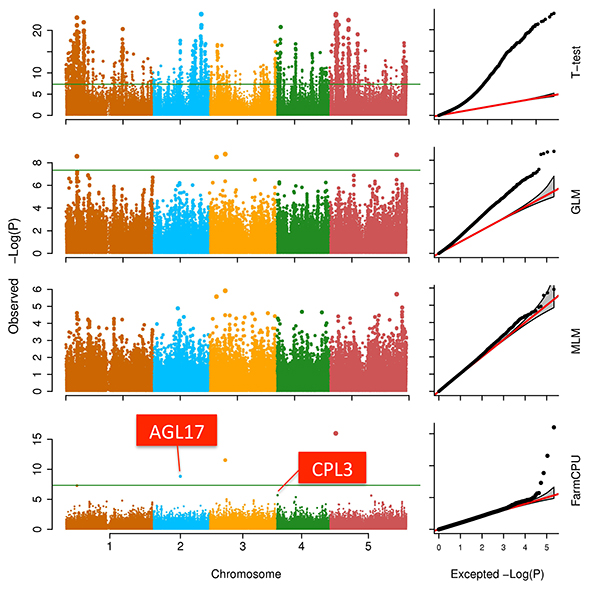
**

**S71 Fig. GWAS results of Days to Flowering, no vernalization, greenhouse (0W GH FT) using four models (naïve model (t-test), GLM, MLM and FarmCPU).**

**
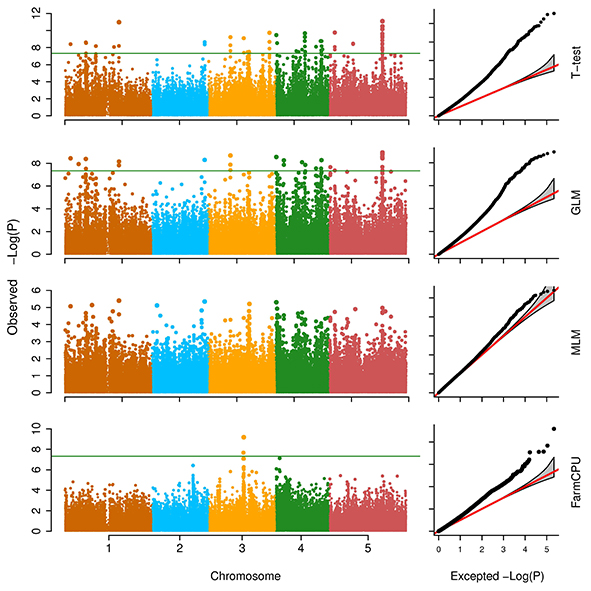
**

**S72 Fig. GWAS results of Leaf Number at Flowering, no vernalization, greenhouse (0W GH LN) using four models (naïve model (t-test), GLM, MLM and FarmCPU).**

**
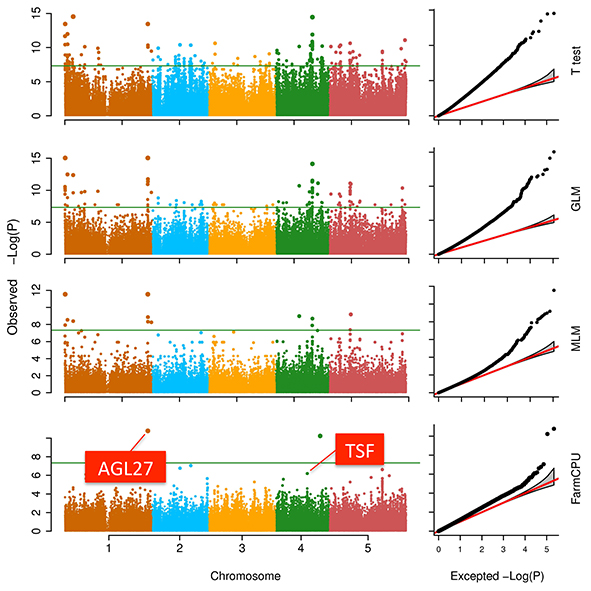
**

**S73 Fig. GWAS results of Days to Flowering, field (FT Field) using four models (naïve model (t-test), GLM, MLM and FarmCPU).**

**
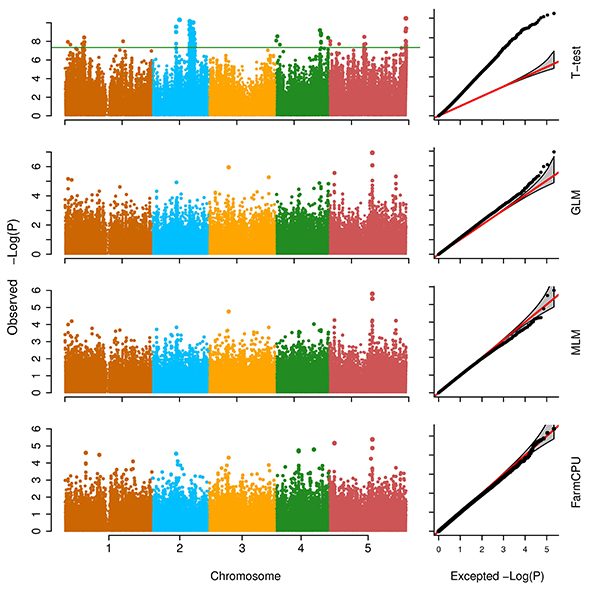
**

**S74 Fig. GWAS results of Diameter of Plants at Flowering, field (FT Diameter Field) using four models (naïve model (t-test), GLM, MLM and FarmCPU).**

**
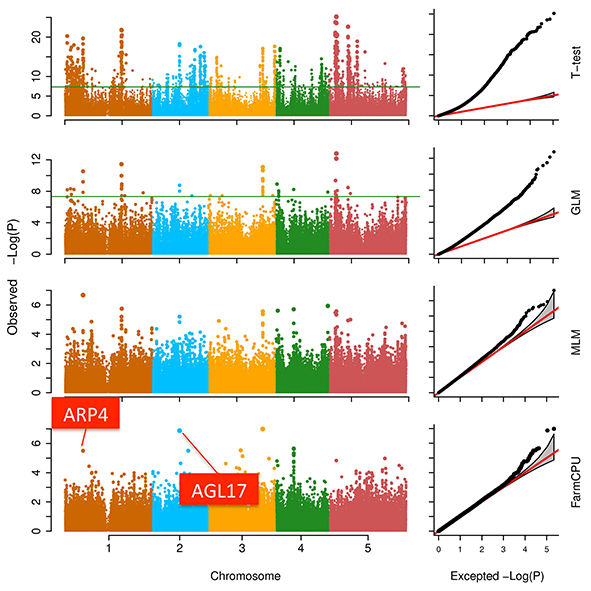
**

**S75 Fig. GWAS results of Days to Flowering, greenhouse (FT GH) using four models (naïve model (t-test), GLM, MLM and FarmCPU).**

**
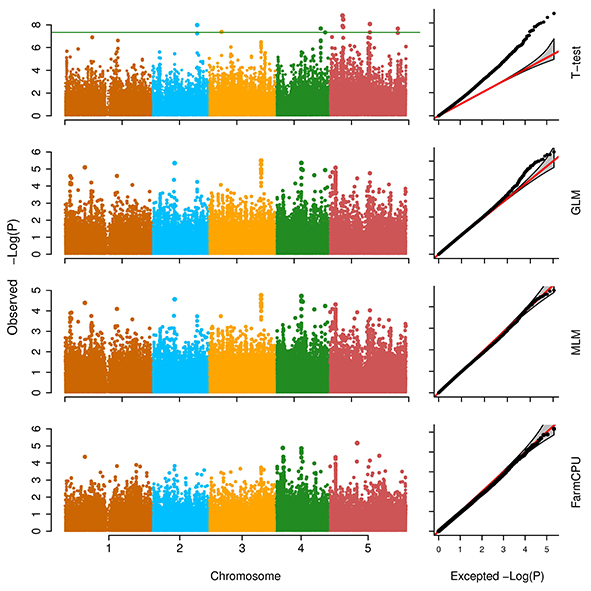
**

**S76 Fig. GWAS results of Duration of Flowering Time, greenhouse (FT duration GH) using four models (naïve model (t-test), GLM, MLM and FarmCPU).**

**
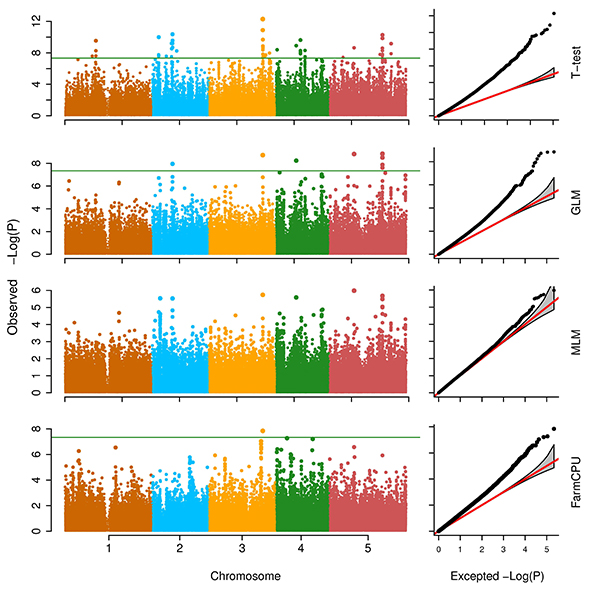
**

**S77 Fig. GWAS results of Life Cycle Duration, greenhouse (LC Duration GH) using four models (naïve model (t-test), GLM, MLM and FarmCPU).**

**
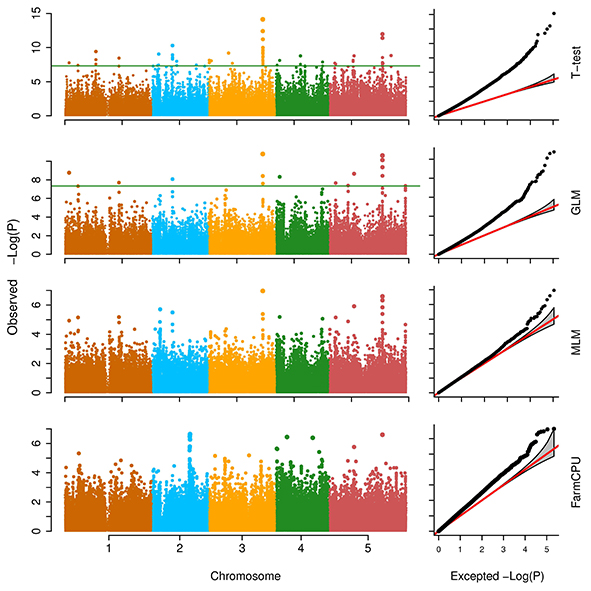
**

**S78 Fig. GWAS results of Length until Flower Senescence, greenhouse (LFS GH) using four models (naïve model (t-test), GLM, MLM and FarmCPU).**

**
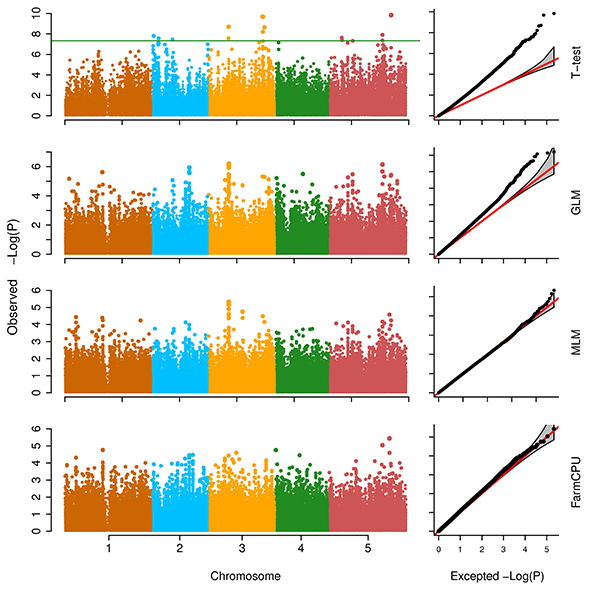
**

**S79 Fig. GWAS results of Time to Maturation, greenhouse (MT GH) using four models (naïve model (t-test), GLM, MLM and FarmCPU).**

**
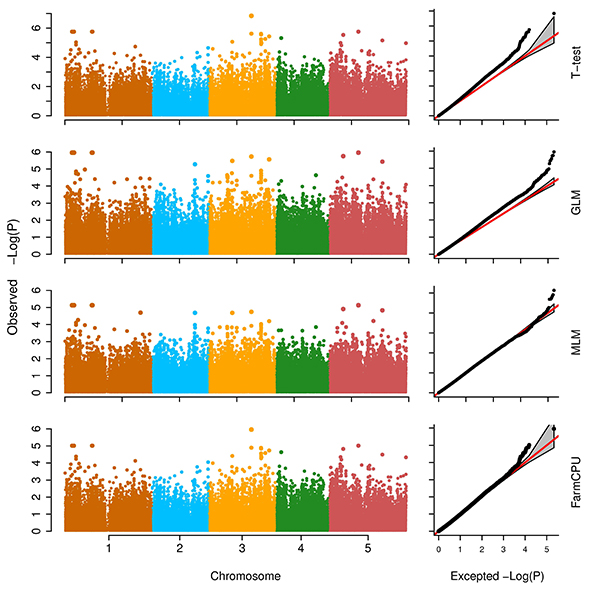
**

**S80 Fig. GWAS results of Reproductive Period, greenhouse (RP GH) using four models (naïve model (t-test), GLM, MLM and FarmCPU).**

**
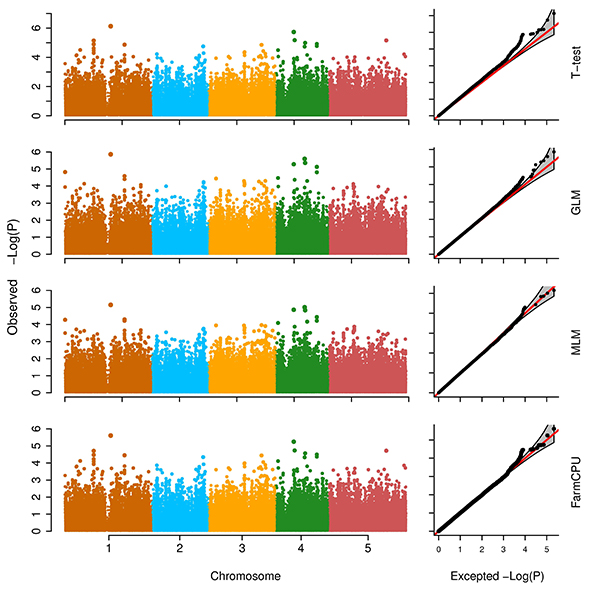
**

**S81 Fig. GWAS results of At1 using four models (naïve model (t-test), GLM, MLM and FarmCPU).**

**
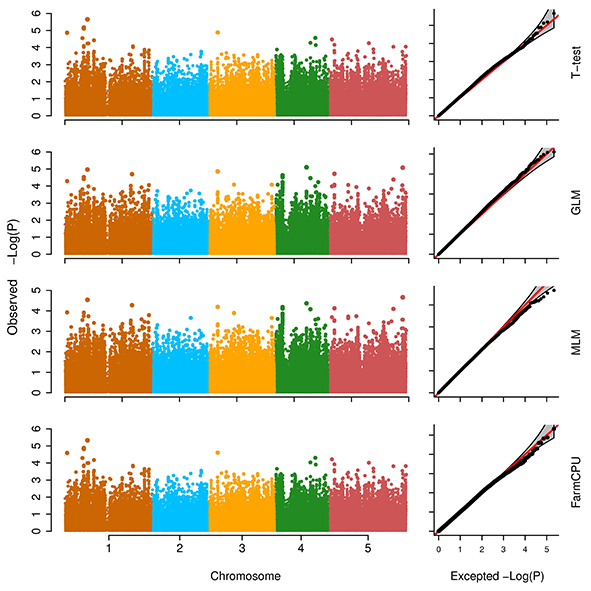
**

**S82 Fig. GWAS results of At1 CFU2 using four models (naïve model (t-test), GLM, MLM and FarmCPU).**

**
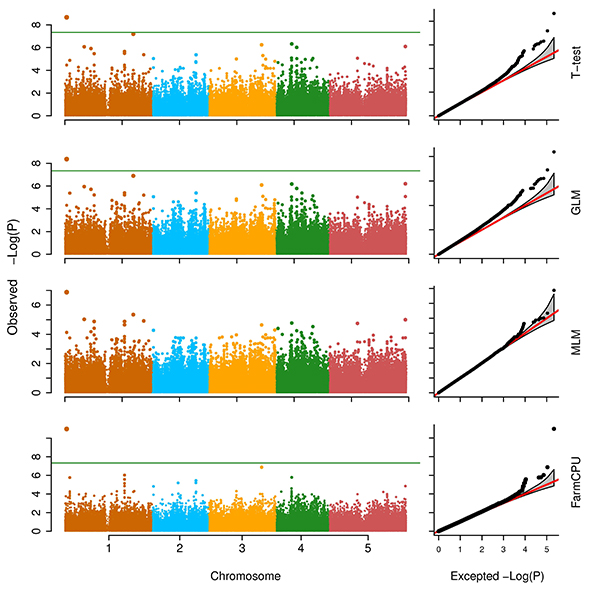
**

**S83 Fig. GWAS results of Arsenic concentration (As) using four models (naïve model (t-test), GLM, MLM and FarmCPU).**

**
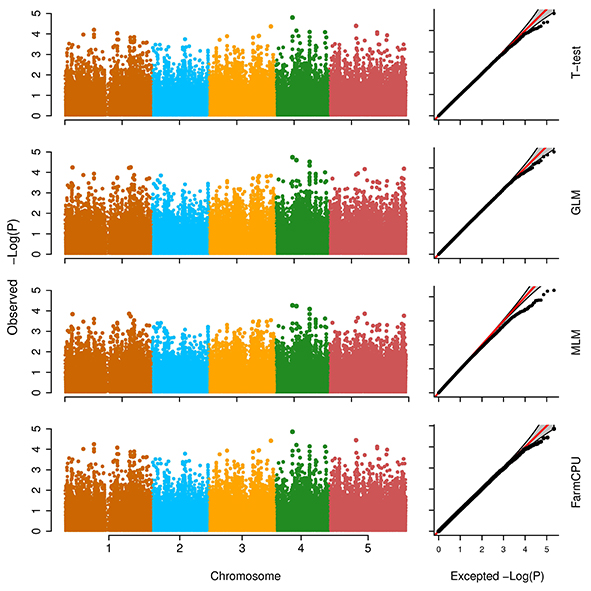
**

**S84 Fig. GWAS results of As CFU2 using four models (naïve model (t-test), GLM, MLM and FarmCPU).**

**
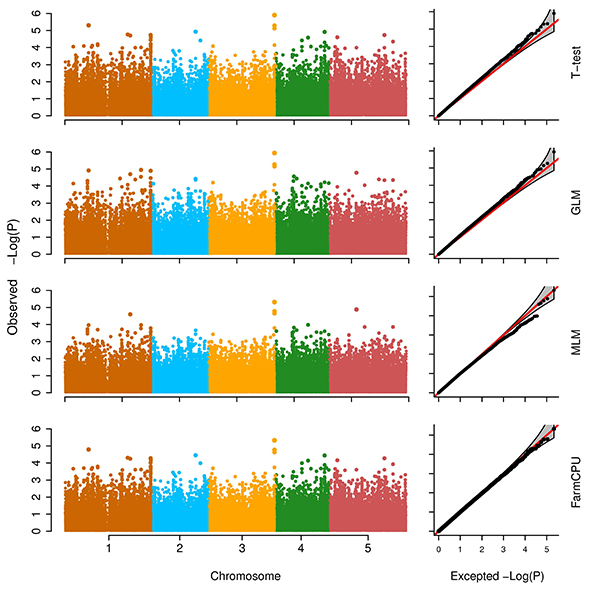
**

**S85 Fig. GWAS results of Bs using four models (naïve model (t-test), GLM, MLM and FarmCPU).**

**
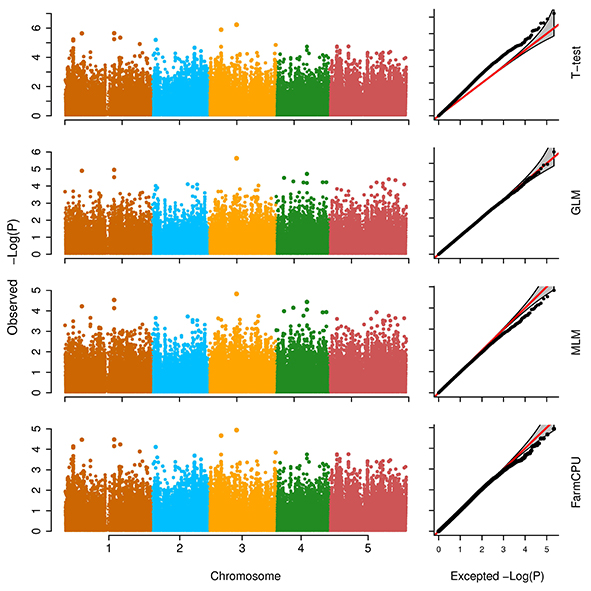
**

**S86 Fig. GWAS results of Bs CFU2 using four models (naïve model (t-test), GLM, MLM and FarmCPU).**

**
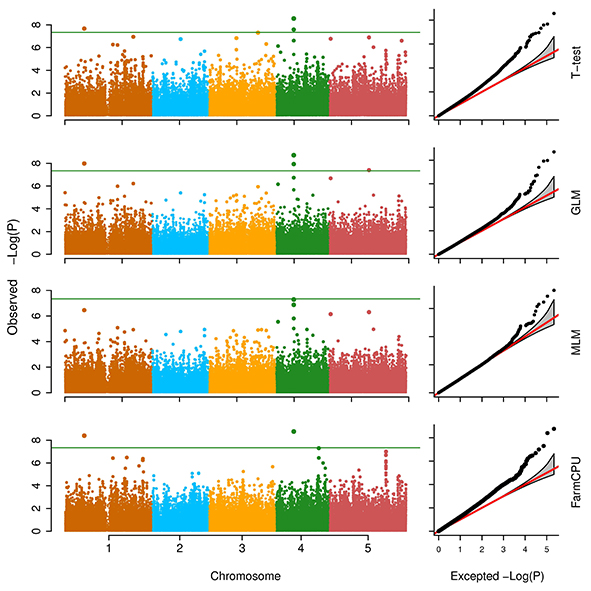
**

**S87 Fig. GWAS results of At2 using four models (naïve model (t-test), GLM, MLM and FarmCPU).**

**
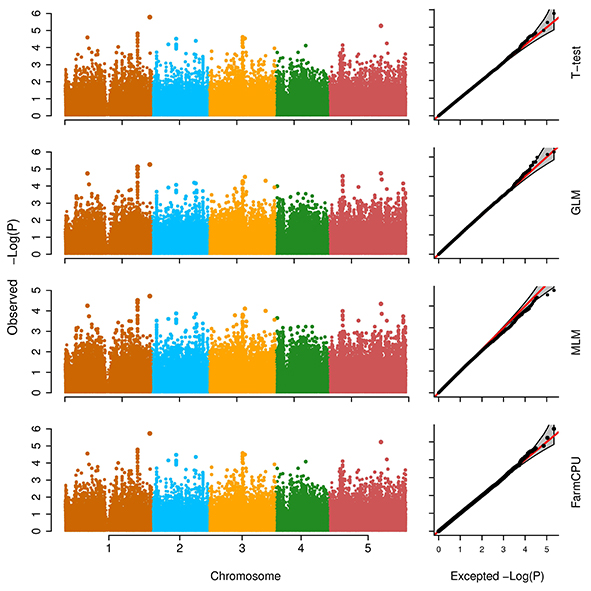
**

**S88 Fig. GWAS results of At2 CFU2 using four models (naïve model (t-test), GLM, MLM and FarmCPU).**

**
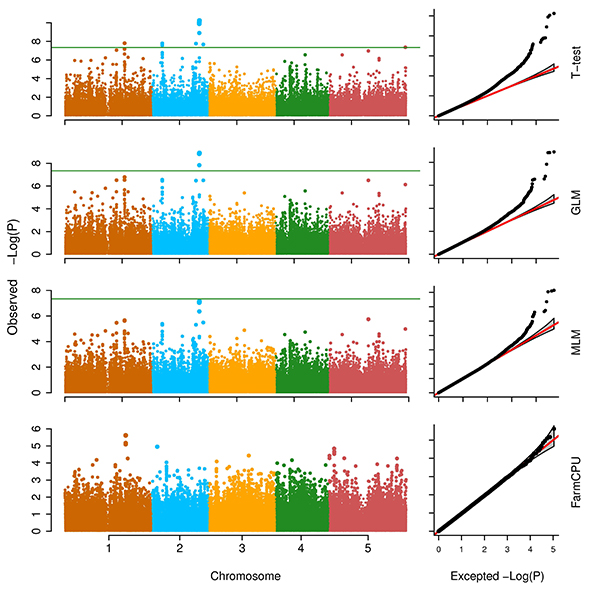
**

**S89 Fig. GWAS results of As2 using four models (naïve model (t-test), GLM, MLM and FarmCPU).**

**
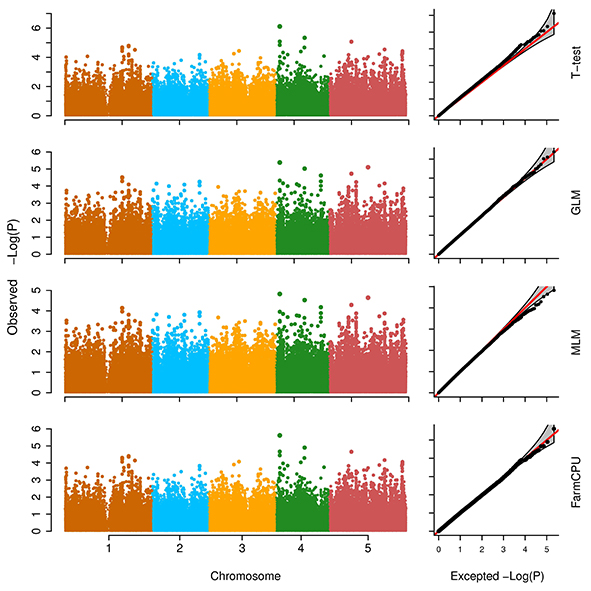
**

**S90 Fig. GWAS results of As2 CFU2 using four models (naïve model (t-test), GLM, MLM and FarmCPU).**

**
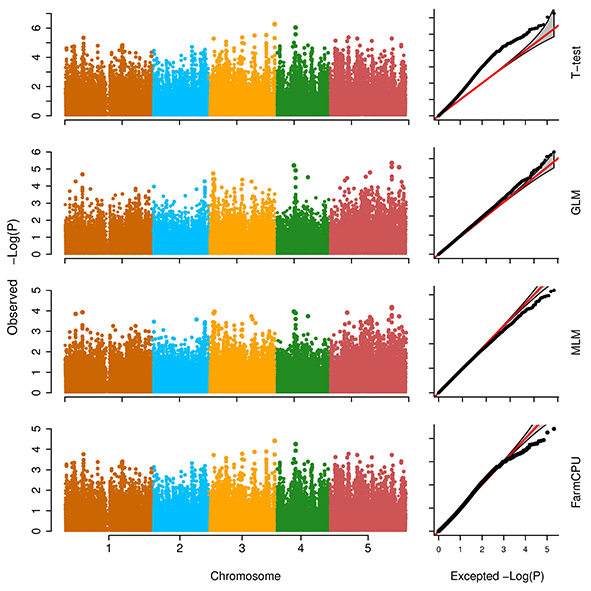
**

**S91 Fig. GWAS results of Fresh Weight (FW) using four models (naïve model (t-test), GLM, MLM and FarmCPU).**

**
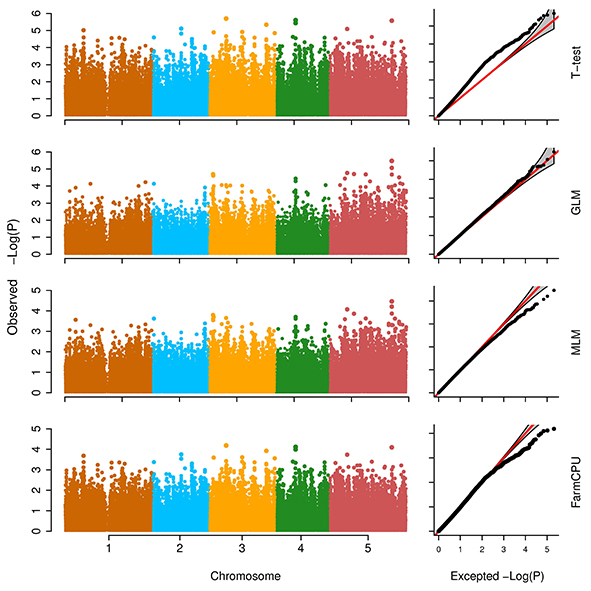
**

**S92 Fig. GWAS results of Dry Weight (DW) using four models (naïve model (t-test), GLM, MLM and FarmCPU).**

**
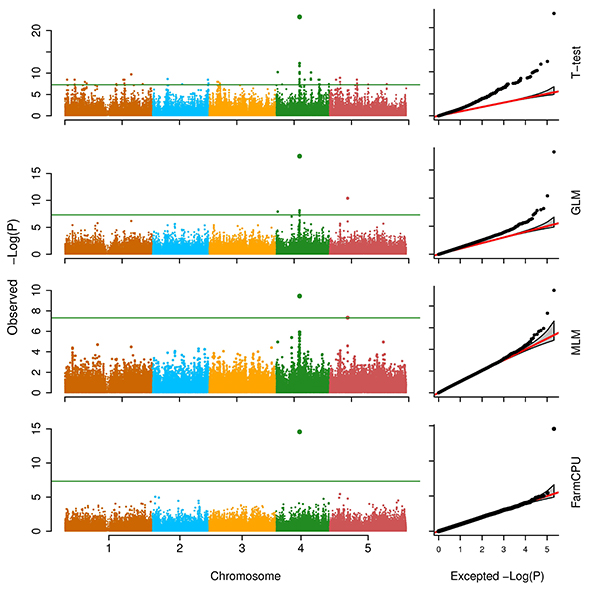
**

**S93 Fig. GWAS results of Lesioning (LES) using four models (naïve model (t-test), GLM, MLM and FarmCPU).**

**
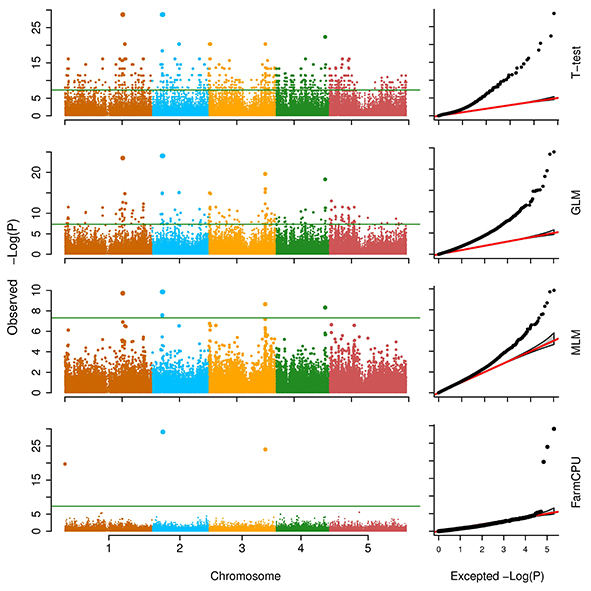
**

**S94 Fig. GWAS results of Yellowing (YEL) using four models (naïve model (t-test), GLM, MLM and FarmCPU).**

**
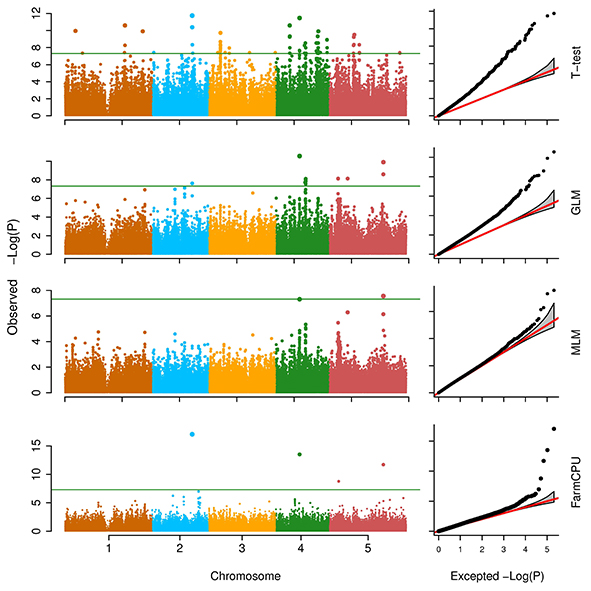
**

**S95 Fig. GWAS results of Lesioning and Yellowing (LY) using four models (naïve model (t-test), GLM, MLM and FarmCPU).**

**
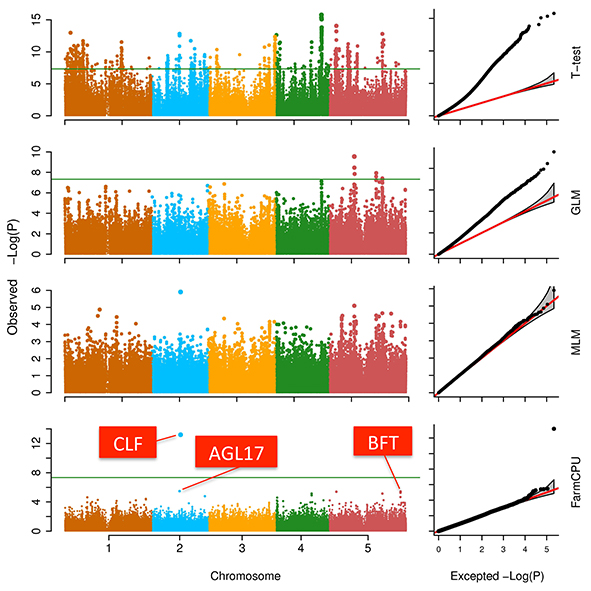
**

**S96 Fig. GWAS results of Leaf Number at 10◦C (LN10) using four models (naïve model (t-test), GLM, MLM and FarmCPU).**

**
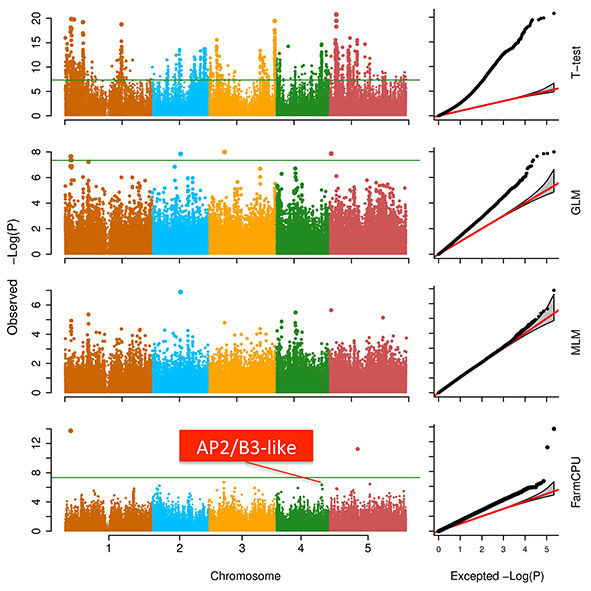
**

**S97 Fig. GWAS results of Leaf Number at 16◦C (LN16) using four models (naïve model (t-test), GLM, MLM and FarmCPU).**

**
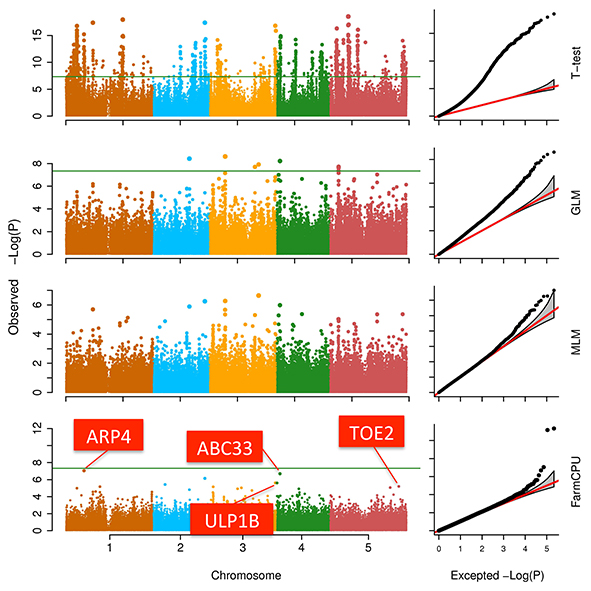
**

**S98 Fig. GWAS results of Leaf Number at 22◦C (LN22) using four models (naïve model (t-test), GLM, MLM and FarmCPU).**

**
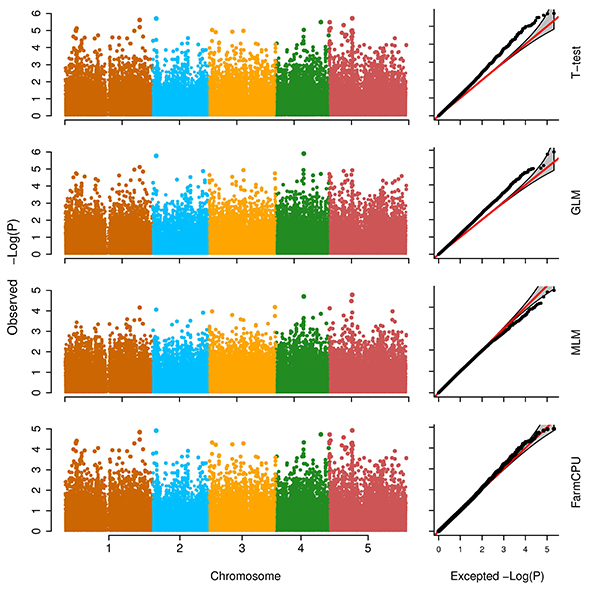
**

**S99 Fig. GWAS results of Silique Length at 16◦C (SL16) using four models (naïve model (t-test), GLM, MLM and FarmCPU).**

**
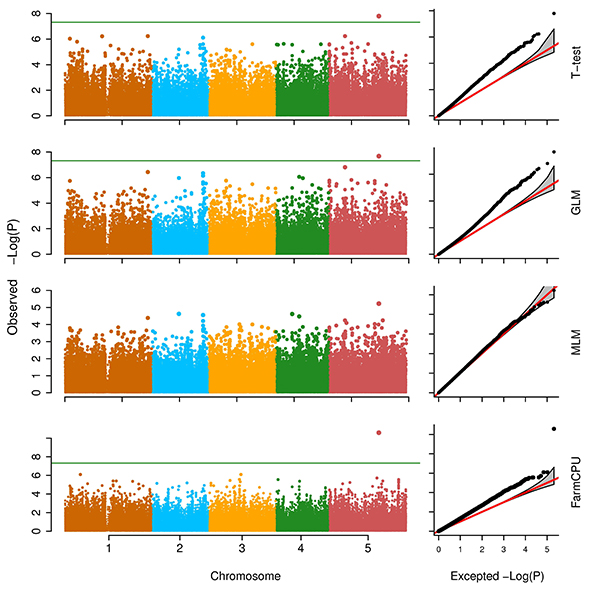
**

**S100 Fig. GWAS results of Silique Length at 22◦C (SL22) using four models (naïve model (t-test), GLM, MLM and FarmCPU).**

**
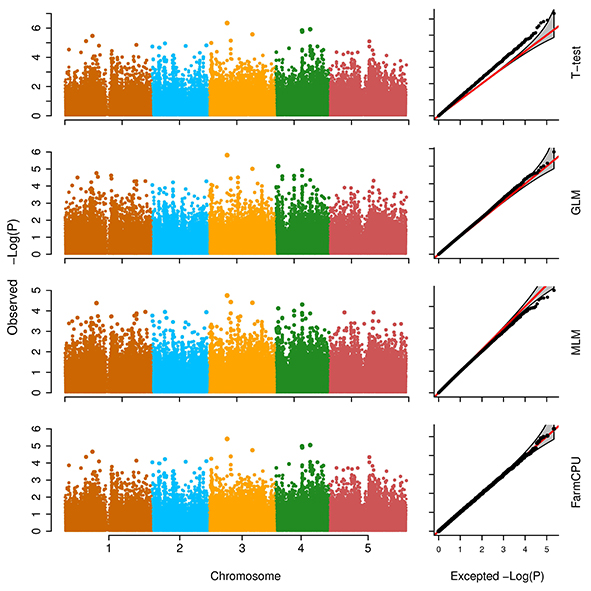
**

**S101 Fig. GWAS results of Days to Germination at 10◦C (DG10) using four models (naïve model (t-test), GLM, MLM and FarmCPU).**

**
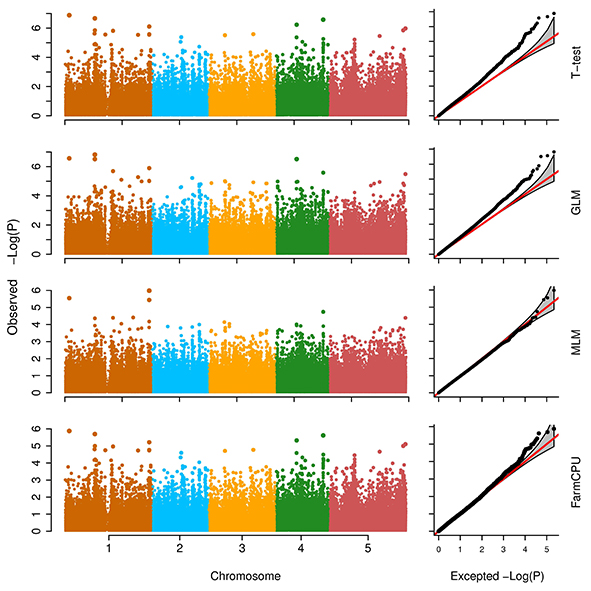
**

**S102 Fig. GWAS results of Days to Germination at 16◦C (DG16) using four models (naïve model (t-test), GLM, MLM and FarmCPU).**

**
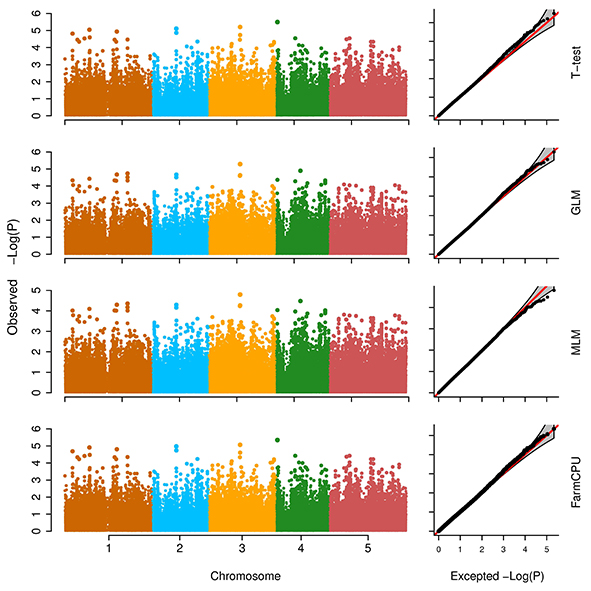
**

**S103 Fig. GWAS results of Days to Germination at 22◦C (DG22) using four models (naïve model (t-test), GLM, MLM and FarmCPU).**

**
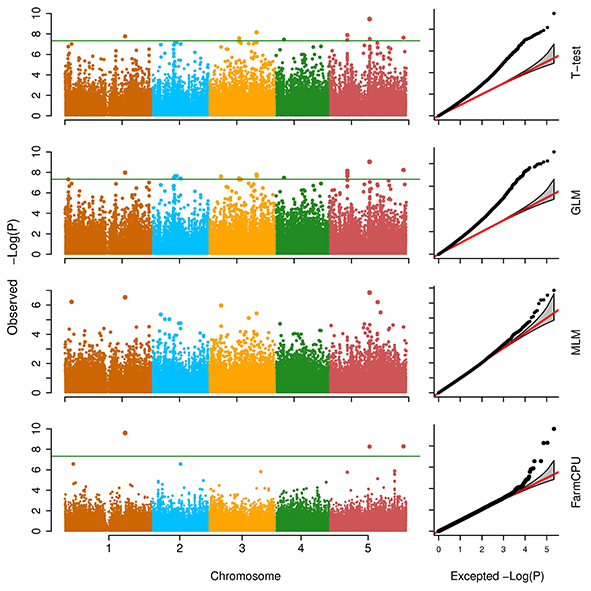
**

**S104 Fig. GWAS results of Plant Diameter at 10◦C (Width 10) using four models (naïve model (t-test), GLM, MLM and FarmCPU).**

**
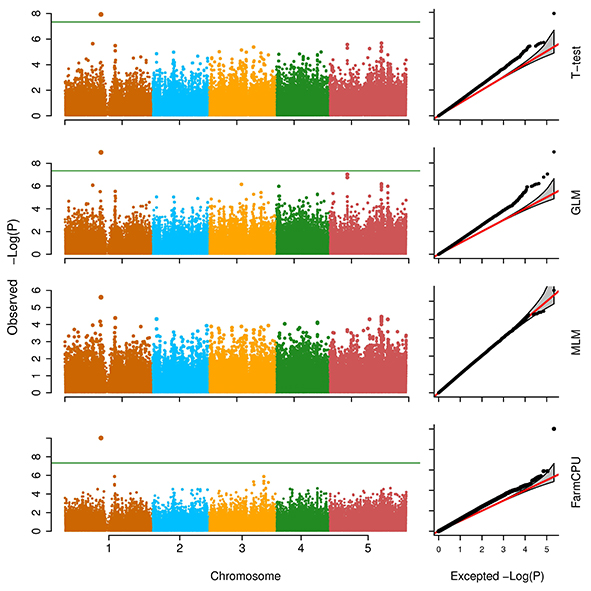
**

**S105 Fig. GWAS results of Plant Diameter at 16◦C (Width 16) using four models (naïve model (t-test), GLM, MLM and FarmCPU).**

**
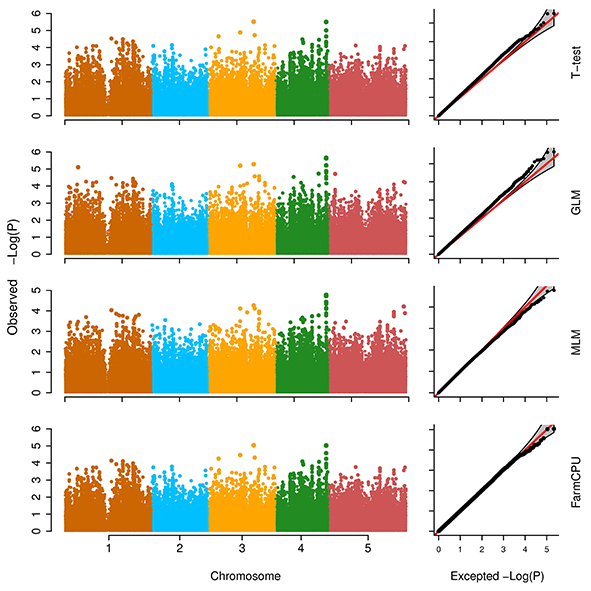
**

**S106 Fig. GWAS results of Plant Diameter at 22◦C (Width 22) using four models (naïve model (t-test), GLM, MLM and FarmCPU).**

**
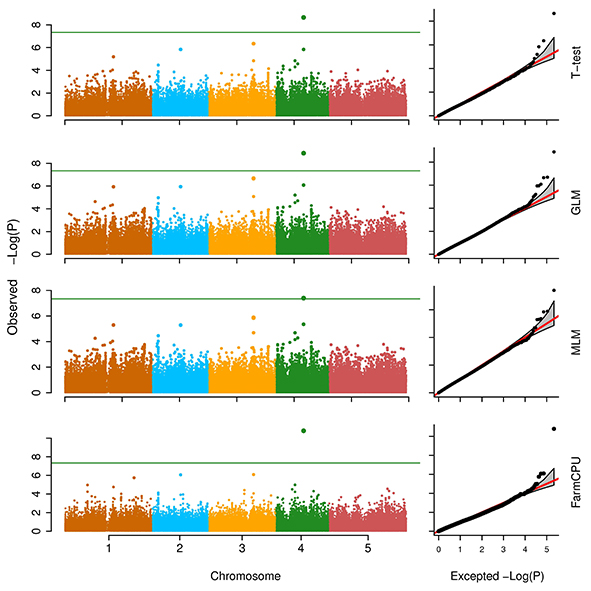
**

**S107 Fig. GWAS results of Presence or Absence of Chlorosis at 10◦C using four models (naïve model (t-test), GLM, MLM and FarmCPU).**

**
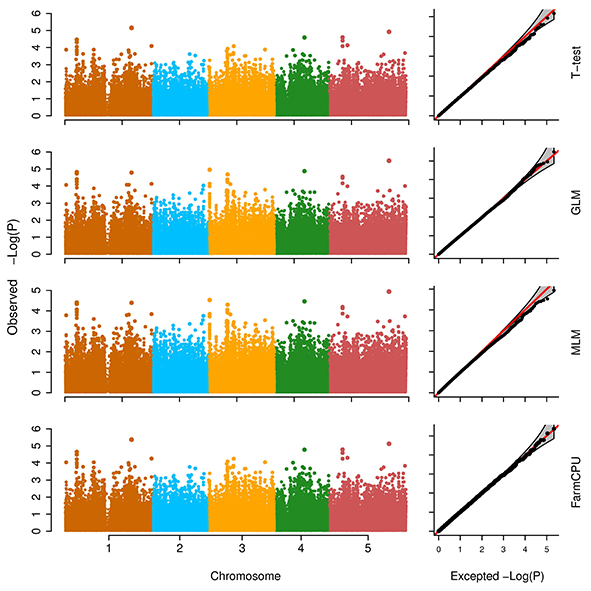
**

**S108 Fig. GWAS results of Presence or Absence of Chlorosis at 16◦C using four models (naïve model (t-test), GLM, MLM and FarmCPU).**

**
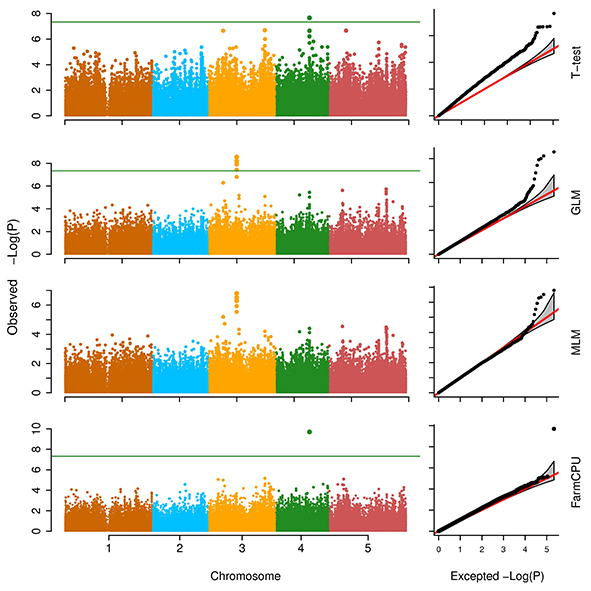
**

**S109 Fig. GWAS results of Presence or Absence of Chlorosis at 22◦C using four models (naïve model (t-test), GLM, MLM and FarmCPU).**

**
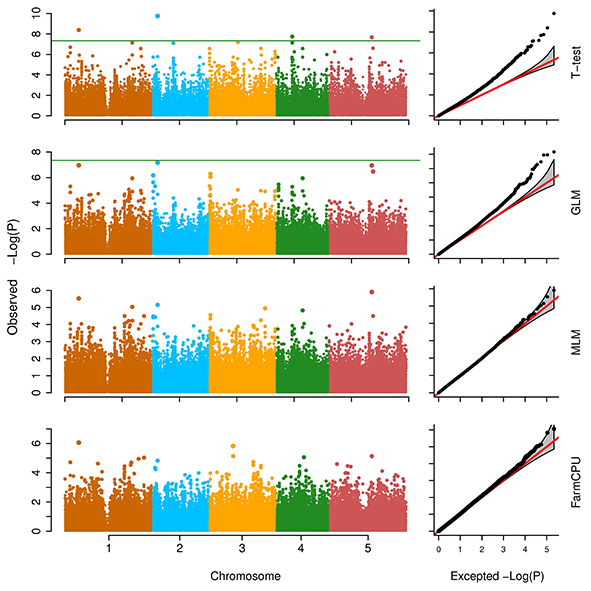
**

**S110 Fig. GWAS results of Presence or Absence of Anthocyanin at 10◦C using four models (naïve model (t-test), GLM, MLM and FarmCPU).**

**
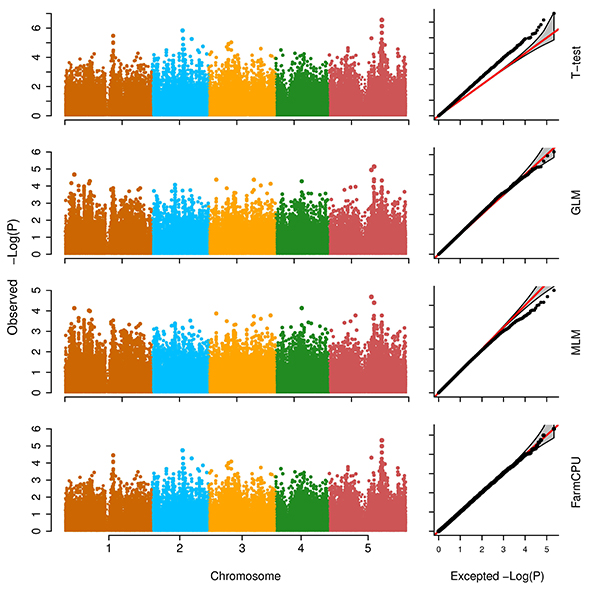
**

**S111 Fig. GWAS results of Presence or Absence of Anthocyanin at 16◦C using four models (naïve model (t-test), GLM, MLM and FarmCPU).**

**
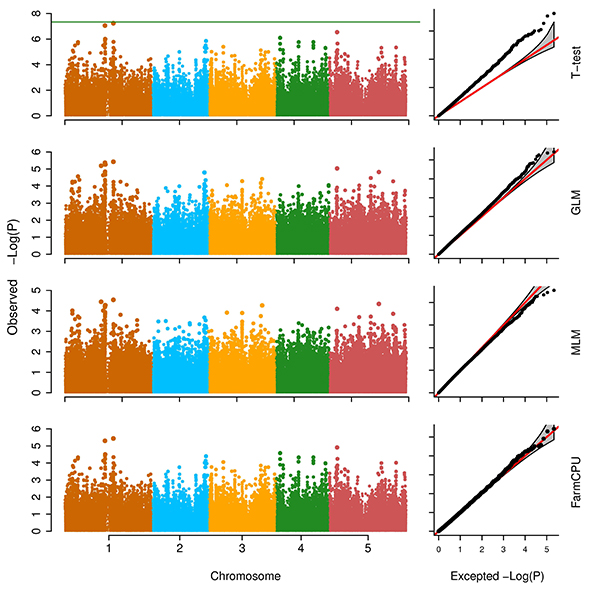
**

**S112 Fig. GWAS results of Presence or Absence of Anthocyanin at 22◦C using four models (naïve model (t-test), GLM, MLM and FarmCPU).**

**
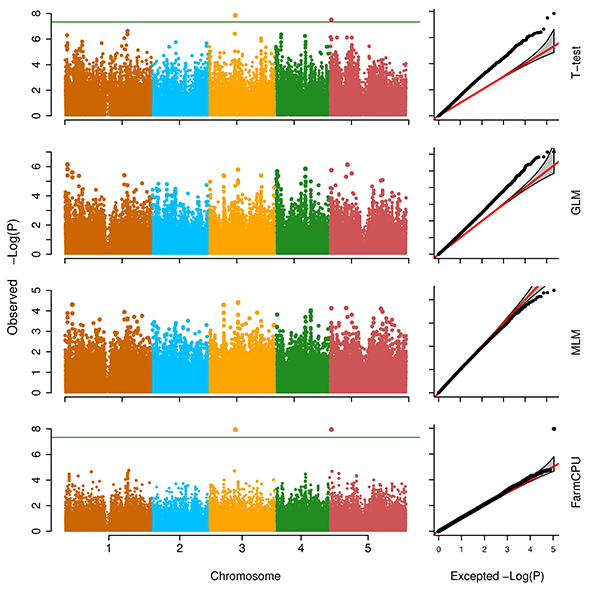
**

**S113 Fig. GWAS results of Presence of Leaf Serration at 10◦C (PLS10) using four models (naïve model (t-test), GLM, MLM and FarmCPU).**

**
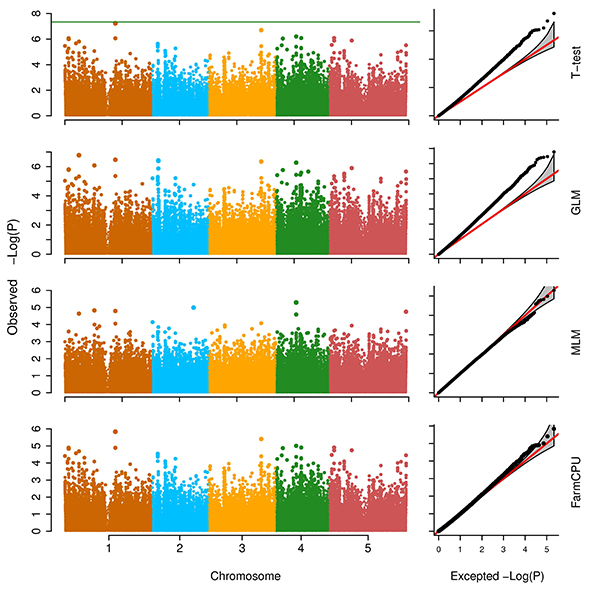
**

**S114 Fig. GWAS results of Presence of Leaf Serration at 16◦C (PLS16) using four models (naïve model (t-test), GLM, MLM and FarmCPU).**

**
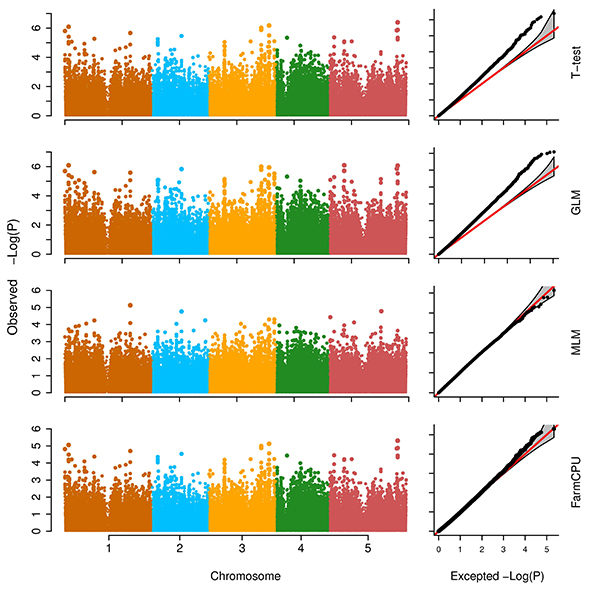
**

**S115 Fig. GWAS results of Presence of Leaf Serration at 22◦C (PLS22) using four models (naïve model (t-test), GLM, MLM and FarmCPU).**

**
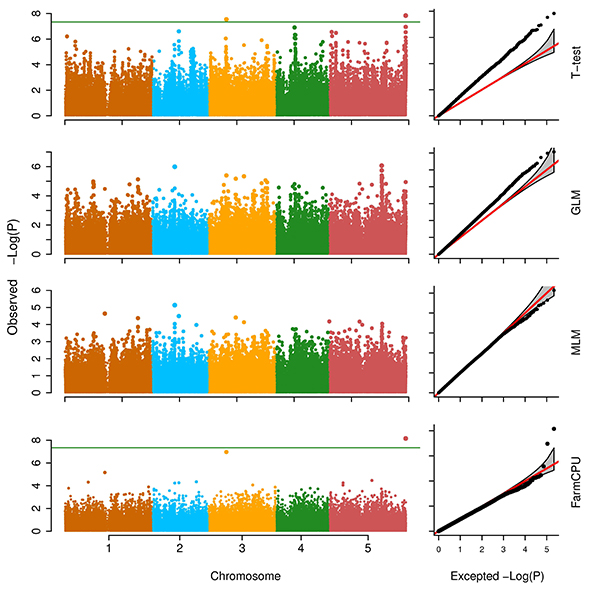
**

**S116 Fig. GWAS results of Presence of Rolled Leaves at 10◦C (PRL10) using four models (naïve model (t-test), GLM, MLM and FarmCPU).**

**
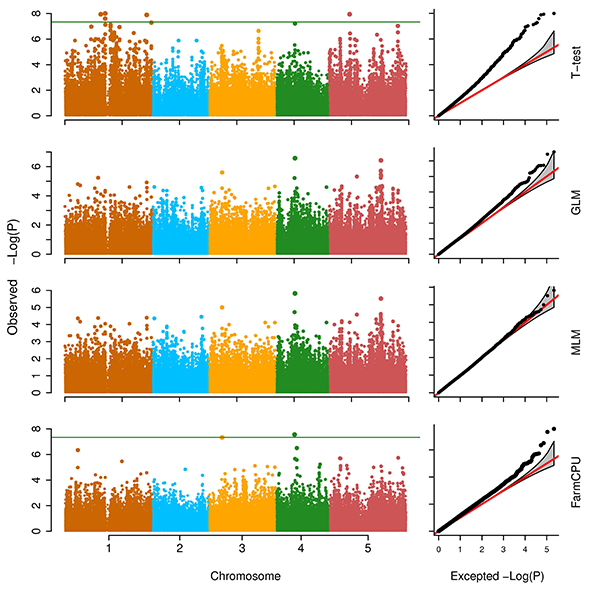
**

**S117 Fig. GWAS results of Presence of Rolled Leaves at 16◦C (PRL16) using four models (naïve model (t-test), GLM, MLM and FarmCPU).**

**
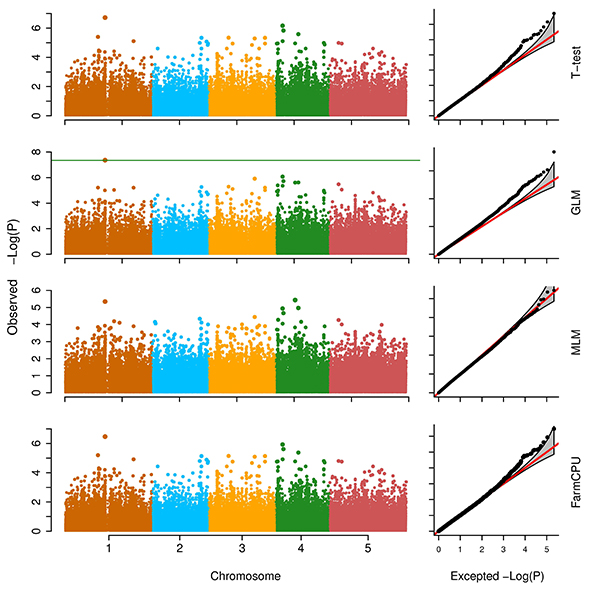
**

**S118 Fig. GWAS results of Presence of Rolled Leaves at 22◦C (PRL22) using four models (naïve model (t-test), GLM, MLM and FarmCPU).**

**
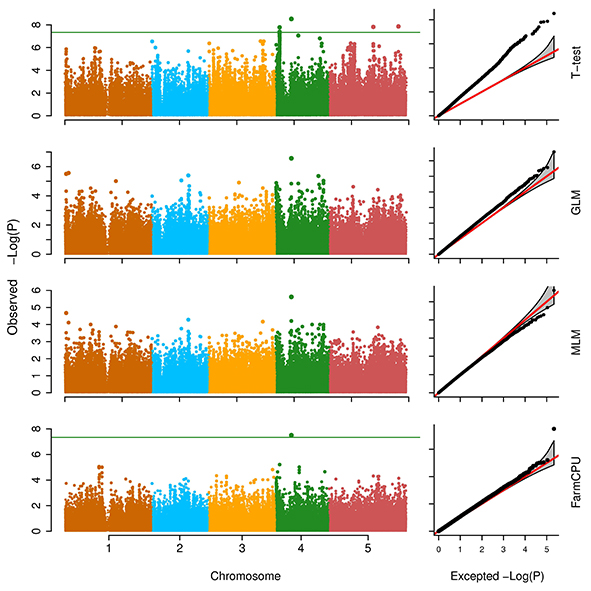
**

**S119 Fig. GWAS results of Presence of Erect Rosette at 22◦C (PER22) using four models (naïve model (t-test), GLM, MLM and FarmCPU).**

**
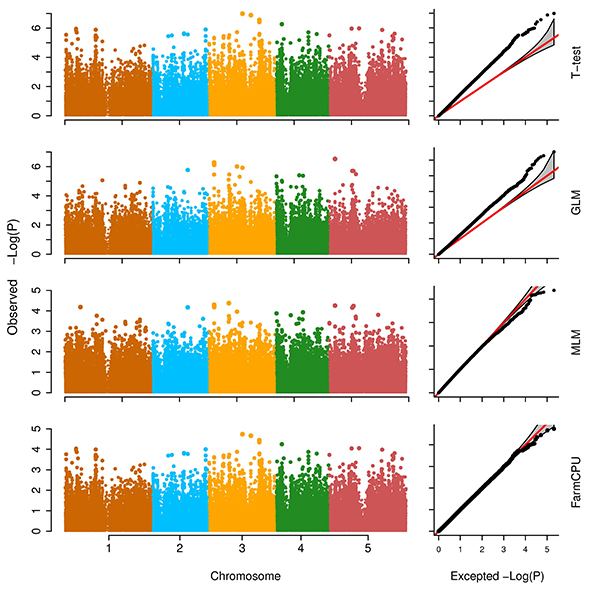
**

**S120 Fig. GWAS results of Hypocotyl Length (HL) using four models (naïve model (t-test), GLM, MLM and FarmCPU).**

**
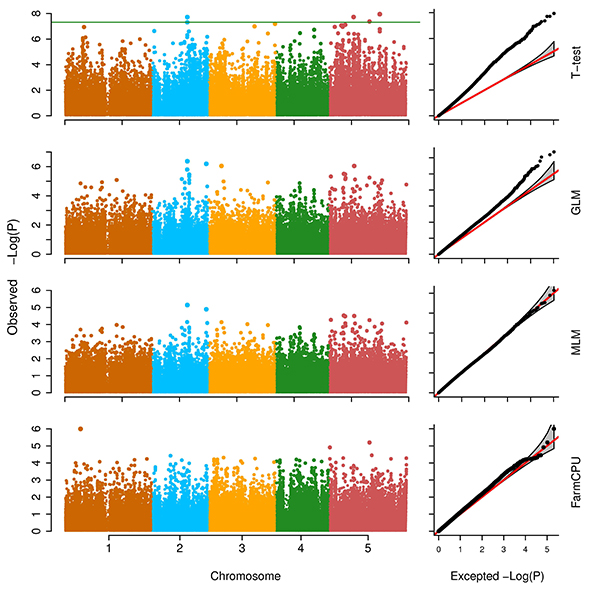
**

**S121 Fig. GWAS results of Trichome Number C using four models (naïve model (t-test), GLM, MLM and FarmCPU).**

**
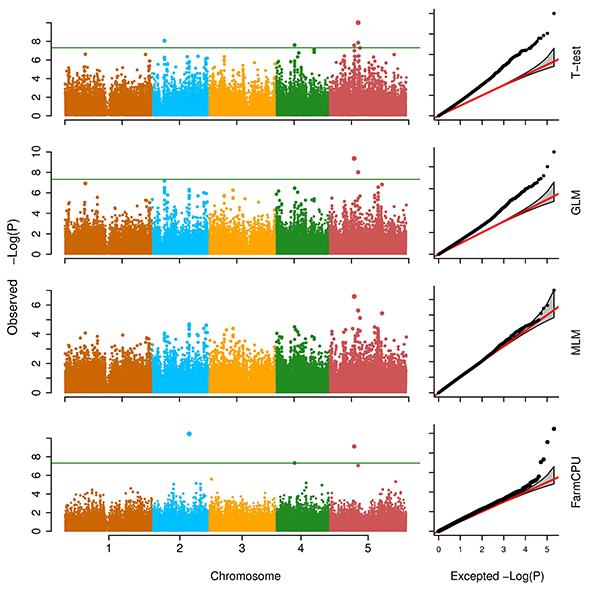
**

**S122 Fig. GWAS results of Trichome Number JA using four models (naïve model (t-test), GLM, MLM and FarmCPU).**

**
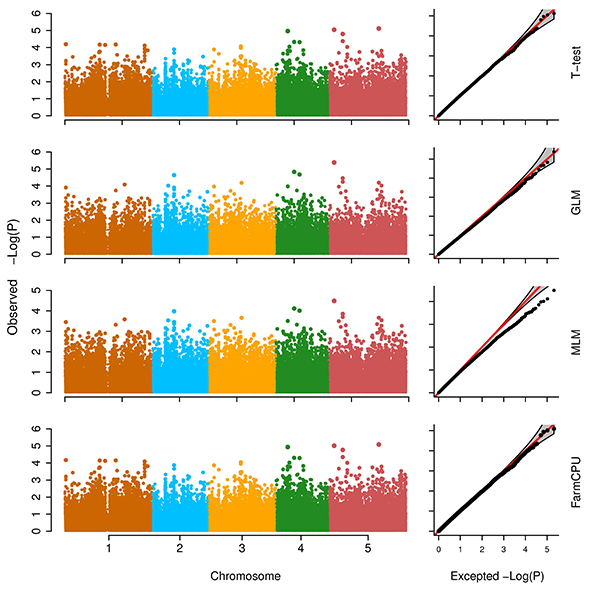
**

**S123 Fig. GWAS results of Aphid Number (AN) using four models (naïve model (t-test), GLM, MLM and FarmCPU).**

**
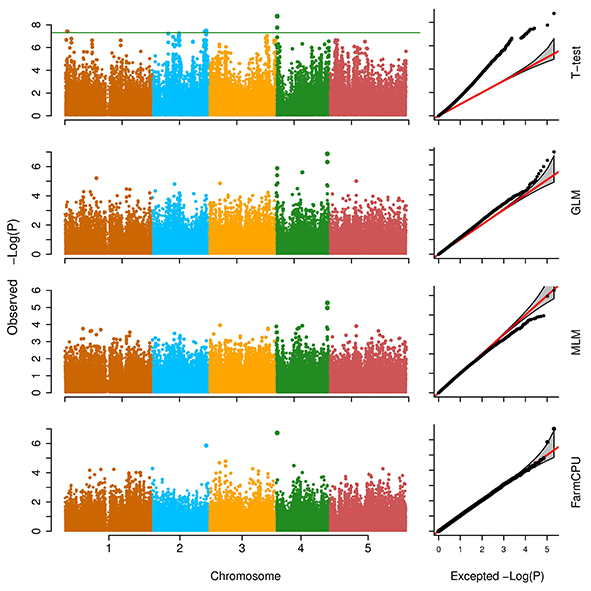
**

**S124 Fig. GWAS results of *Pseudomonas syringae pv. tomato* DC3000 using four models (naïve model (t-test), GLM, MLM and FarmCPU).**

**
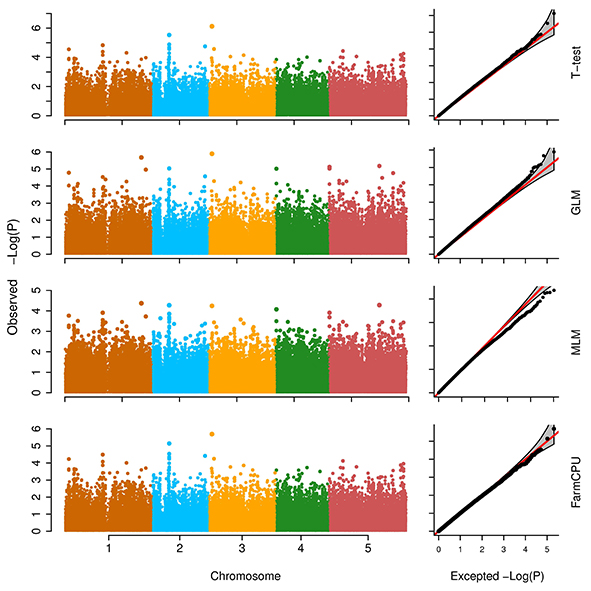
**

**S125 Fig. GWAS results of Seedling Growth (SG) using four models (naïve model (t-test), GLM, MLM and FarmCPU).**

**
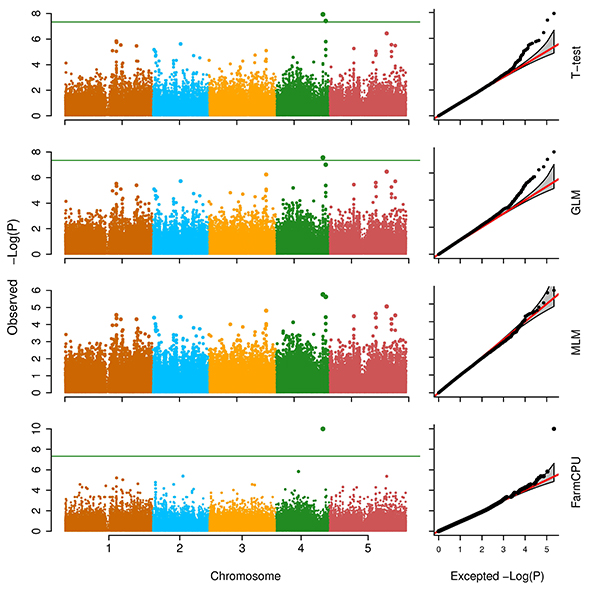
**

**S126 Fig. GWAS results of Vegetative Growth Rate during Vernalization (Vern growth) using four models (naïve model (t-test), GLM, MLM and FarmCPU).**

**
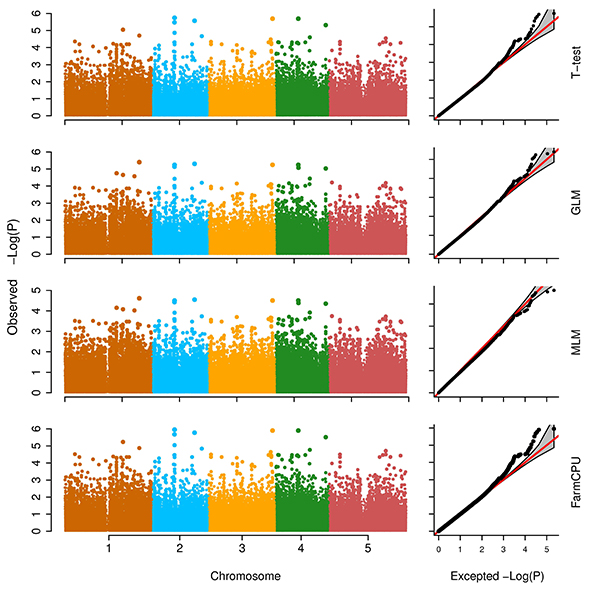
**

**S127 Fig. GWAS results of Vegetative Growth Rate after Vernalization (After vern growth) using four models (naïve model (t-test), GLM, MLM and FarmCPU).**

**
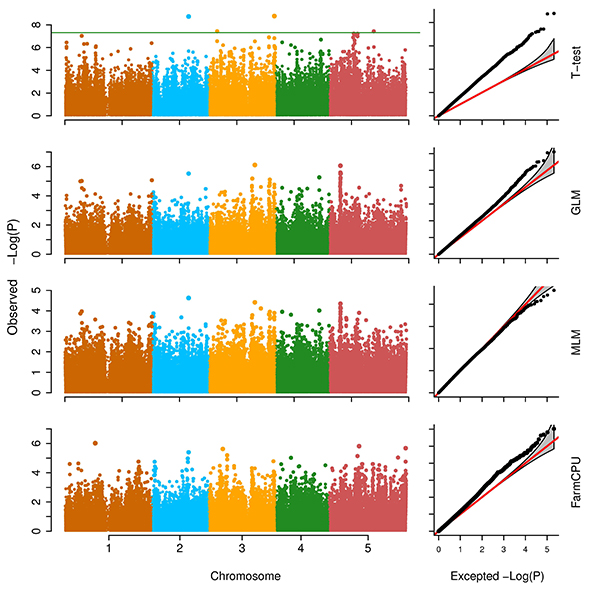
**

**S128 Fig. GWAS results of Secondary Dormancy (SD) using four models (naïve model (t-test), GLM, MLM and FarmCPU).**

**S129 Fig. GWAS results of Germination in the Dark (Germ 4C 7d) using four models (naïve model (t-test), GLM, MLM and FarmCPU).**

**S130 Fig. GWAS results of Seed Dormancy Level (DSDS50) using four models (naïve model (t-test), GLM, MLM and FarmCPU).**

**S131 Fig. GWAS results of Reduction in Germination Rate (Seed bank 133-91) using four models (naïve model (t-test), GLM, MLM and FarmCPU).**

**S132 Fig. GWAS results of Primary Dormancy with 7 Days Dry Storage (Storage 7 days) using four models (naïve model (t-test), GLM, MLM and FarmCPU).**

**S133 Fig. GWAS results of Primary Dormancy with 28 Days Dry Storage (Storage 28 days) using four models (naïve model (t-test), GLM, MLM and FarmCPU).**

**S134 Fig. GWAS results of Primary Dormancy with 56 Days Dry Storage (Storage 56 days) using four models (naïve model (t-test), GLM, MLM and FarmCPU).**
